# Evaluation and Forecasting Analysis of the Association of Conditional Cash Transfer With Child Mortality in Latin America, 2000-2030

**DOI:** 10.1001/jamanetworkopen.2023.23489

**Published:** 2023-07-14

**Authors:** Daniella Medeiros Cavalcanti, José Alejandro Ordoñez, Temidayo Aransiola, Cristina Almeida, Juan Felipe Perdomo Díaz, Daniela Zuluaga Mayorga, Alejandro Zamudio Sosa, Renato Tasca, Tereza Campello, Luis Eugenio de Souza, Philipp Hessel, Carlos Chivardi, Ana L. Moncayo, Davide Rasella

**Affiliations:** 1 Institute of Collective Health at the Federal University of Bahia, Bahia, Brazil; 2 Centro de Investigación para la Salud en América Latina, Pontificia Universidad Católica del Ecuador, Quito, Ecuador; 3 Alberto Lleras Camargo School of Government, Universidad de los Andes, Bogotá, Colombia; 4 Health Research Consortium, Cuernavaca, Mexico; 5 Institute of Studies for Health Policies, Rio de Janeiro, Brazil; 6 Center for Epidemiological Research in Nutrition and Health at the University of São Paulo, São Paulo, Brazil; 7 Swiss Tropical and Public Health Institute, Department of Public Health and Epidemiology, Basel, Switzerland; 8 Center for Health Economics, University of York, York, England; 9 Institute of Global Health (ISGlobal), Hospital Clínic—Universitat de Barcelona, Barcelona, Spain

## Abstract

**Importance:**

Latin America has implemented the world’s largest and most consolidated conditional cash transfer (CCT) programs during the last 2 decades. As a consequence of the COVID-19 pandemic, poverty rates have markedly increased, and a large number of newly low-income individuals, especially children, have been left unprotected.

**Objective:**

To evaluate the association of CCT programs with child health in Latin American countries during the last 2 decades and forecast child mortality trends up to 2030 according to CCT alternative implementation options.

**Design, Setting, and Participants:**

This cohort study used a multicountry, longitudinal, ecological design with multivariable negative binomial regression models, which were adjusted for all relevant demographic, socioeconomic, and health care variables, integrating the retrospective impact evaluations from January 1, 2000, to December 31, 2019, with dynamic microsimulation models to forecast potential child mortality scenarios up to 2030. The study cohort included 4882 municipalities from Brazil, Ecuador, and Mexico with adequate quality of civil registration and vital statistics according to a validated multidimensional criterion. Data analysis was performed from September 2022 to February 2023.

**Exposure:**

Conditional cash transfer coverage of the target (lowest-income) population categorized into 4 levels: low (0%-29.9%), intermediate (30.0%-69.9%), high (70.0%-99.9%), and consolidated (≥100%).

**Main Outcomes and Measures:**

The main outcomes were mortality rates for those younger than 5 years and hospitalization rates (per 1000 live births), overall and by poverty-related causes (diarrheal, malnutrition, tuberculosis, malaria, lower respiratory tract infections, and HIV/AIDS), and the mortality rates for those younger than 5 years by age groups, namely, neonatal (0-28 days), postneonatal (28 days to 1 year), infant (<1 year), and toddler (1-4 years).

**Results:**

The retrospective analysis included 4882 municipalities. During the study period of January 1, 2000, to December 31, 2019, mortality in Brazil, Ecuador, and Mexico decreased by 7.8% in children and 6.5% in infants, and an increase in coverage of CCT programs of 76.8% was observed in these Latin American countries. Conditional cash transfer programs were associated with significant reductions of mortality rates in those younger than 5 years (rate ratio [RR], 0.76; 95% CI, 0.75-0.76), having prevented 738 919 (95% CI, 695 641-782 104) child deaths during this period. The association of highest coverage of CCT programs was stronger with poverty-related diseases, such as malnutrition (RR, 0.33; 95% CI, 0.31-0.35), diarrhea (RR, 0.41; 95% CI, 0.40-0.43), lower respiratory tract infections (RR, 0.66, 95% CI, 0.65-0.68), malaria (RR, 0.76; 95% CI, 0.63-0.93), tuberculosis (RR, 0.62; 95% CI, 0.48-0.79), and HIV/AIDS (RR, 0.32; 95% CI, 0.28-0.37). Several sensitivity and triangulation analyses confirmed the robustness of the results. Considering a scenario of moderate economic crisis, a mitigation strategy that will increase the coverage of CCTs to protect those newly in poverty could reduce the mortality rate for those younger than 5 years by up to 17% (RR, 0.83; 95% CI, 0.80-0.85) and prevent 153 601 (95% CI, 127 441-180 600) child deaths by 2030 in Brazil, Ecuador, and Mexico.

**Conclusions and Relevance:**

The results of this cohort study suggest that the expansion of CCT programs could strongly reduce childhood hospitalization and mortality in Latin America and should be considered an effective strategy to mitigate the health impact of the current global economic crisis in low- and middle-income countries.

## Key Points

**Question:**

Are conditional cash transfer (CCT) programs associated with lower child mortality in Latin American and Caribbean countries?

**Findings:**

In this cohort study of 4882 municipalities in Brazil, Ecuador, and Mexico for 20 years (2000-2019), CCT coverages were associated with reductions of 24% in overall mortality in those younger than 5 years (with an even stronger association with poverty-related diseases). Forecast modeling suggested that CCT expansion during economic crises could prevent more than 150 000 child deaths in Brazil, Ecuador, and Mexico by 2030.

**Meaning:**

These findings suggest that the expansion of CCT programs could be considered an effective strategy to mitigate the health impact of the current global economic crisis in low- to middle-income countries.

## Teaser

This cohort study of municipalities from Brazil, Ecuador, and Mexico evaluates the association of conditional cash transfer programs with child morbidity and mortality.

## Introduction

The COVID-19 pandemic caused the most significant setback to global poverty in decades, exacerbated by climate shocks and conflicts among the world’s biggest food producers.^1,2^ These 3 C’s (COVID-19, climate change, and conflict) have strongly contributed to slowing progress in the achievement of the sustainable development goals, reversing years of progress in poverty reduction.^3^ There have been repeated calls to expand poverty reduction interventions to the those newly in poverty as a potential mitigation policy, as such interventions may have a larger impact on income growth compared with other interventions and are a far more effective mechanism for supporting vulnerable groups.^1^ The most consolidated poverty reduction policies worldwide are arguably conditional cash transfers (CCTs), which aim to mitigate poverty and break the intergenerational cycle of poverty.^4^ Conditional cash transfer programs transfer cash to low-income households with the requirement that parents comply with specific conditions (or conditionalities), usually focused on health and education for their children. Conditional cash transfer programs have been first implemented in countries in Latin America,^5^ which are among the world’s most unequal countries in terms of socioeconomic and health inequalities.^4^ The first nationwide CCT program was the Progresa Program, which was launched in Mexico in 1997 and became a benchmark program for the region.^6^ Currently, after 25 years, there are 30 active CCT programs in Latin American countries,^5^ and among them are the world’s largest CCT program—the Bolsa Família Program in Brazil^7^—and the CCT program that costs the highest percentage of gross domestic product—the Bono de Desarrollo Humano Program in Ecuador^8^ (eAppendix in Supplement 1). Country-specific studies^9,10,11,12,13,14,15^ have shown the positive association of CCTs with the use of preventive services, the promotion of healthy behavior, and the improvement of a wide range of health outcomes, including child and maternal health and poverty-related diseases. However, to date, no studies have comprehensively evaluated the effectiveness of CCTs on a wide range of child health outcomes in multiple countries. Moreover, none to our knowledge have ever combined retrospective and forecasting analyses to project the impact of CCTs on future child mortality scenarios. The objectives of this study were to evaluate the association of the CCT programs in Brazil, Ecuador, and Mexico with child morbidity and mortality during the last 2 decades and forecast their potential mitigation effects.

## Methods

### Study Design

This multicountry cohort study had a longitudinal, ecological design with municipalities as the unit of analysis, integrating a retrospective impact evaluation in Brazil, Ecuador, and Mexico from January 1, 2000, to December 31, 2019, with dynamic microsimulation models to forecast potential child mortality scenarios up to 2030. According to the internal review board rules of the Institute of Collective Health at the Federal University of Bahia, due to the exclusive use of aggregate secondary data, no ethical approval was necessary. This study followed the Strengthening the Reporting of Observational Studies in Epidemiology (STROBE) reporting guideline and the International Society for Pharmacoeconomics and Outcomes Research and Society for Medical Decision Making (ISPOR-SMDM) international model reporting guideline.^16^

We created a longitudinal data set combining aggregated CCT coverage, demographic, socioeconomic, and health data from Brazil, Ecuador, and Mexico for the years 2000 to 2019. All data used in this study are publicly available from the sources listed in the eAppendix in Supplement 1. From the 8103 municipalities in Brazil, Ecuador, and Mexico, we selected a subset of 4882 municipalities, as in previous studies^9,12^ with similar methods, with adequate quality of civil registration and vital statistics (CRVS) according to a validated multidimensional criterion that considered the value of the age-standardized mortality rate of the municipality, the ratio between registered and estimated birth rates, the percentage of poorly defined deaths, and the mean deviation of the previous parameters (eAppendix in Supplement 1).^17^ We also estimate models considering all municipalities (eAppendix in Supplement 1), and models with a weighting of each observation based on the municipal population have been fitted and are presented in the eAppendix in Supplement 1.

Child mortality (and morbidity) rates were calculated by the number of deaths (and hospitalization) of children younger than 5 years per 1000 live births. We also calculated the mortality rates by other age groups younger than 5 years, namely, neonatal (0- 28 days), postneonatal (28 days to 1 year), infant (<1 year), and toddler (1-4 years), each per 1000 live births. We also selected groups of poverty-related causes of death based on the literature and previous studies,^9,11,18^ using the definitions from the *International Statistical Classification of Diseases and Related Health Problems, Tenth Revision (ICD-10)*: diarrheal diseases (A00, A01, A03, A04, A06-09), tuberculosis (A15-19), HIV/AIDS (B20-24), malaria (B50-54), malnutrition (E40–46), lower respiratory tract infections (J10-18, J20-22), and external causes (W01-Y99).

As a CCT coverage measure, we used the most consolidated indicator^13,15^: the coverage of the target population, calculated as the number of families enrolled in the Progresa Program, Bolsa Família Program, and Bono de Desarrollo Humano Program in each municipality divided by the number of low-income families in the same municipality and year according to each program’s eligibility criteria. Furthermore, to evaluate the dose-response association with increasing degrees of implementation of the interventions, the CCT target population coverage was categorized into 4 levels using previously established reference thresholds^13,15^: low (0%-29.9%), intermediate (30.0%-69.9%), high (70.0%-99.9%), and consolidated (≥100%) (eMethods in Supplement 1).

The models were adjusted for all relevant confounding variables of the association between CCT programs and child health outcomes according to the literature^9,11,18^: Gini index, poverty rate, percentage of illiteracy among individuals older than 15 years, percentage of households with adequate sewers and piped water, number of beds per 1000 population, and number of physicians per 1000 population. As in previous studies,^9,13,15,18^ when information was not available in specific years, consolidated data interpolation techniques were used (eAppendix in Supplement 1). All independent variables were dichotomized by the median value for each country, as in similar evaluations,^9,13,15,18^ and a wide range of other specifications and covariates were also tested in the sensitivity analyses (eAppendix in Supplement 1). In addition, we included a broad set of time dummy variables with 2 specific goals: first, to control changes in CCT programs (new implementation rules and eligibility criteria); and second, to adjust for major economic shocks that occurred in Latin America in the last 2 decades (including the 2007-2008 global subprime crisis and local economic crises).^19,20^ Each methodologic choice, together with the corresponding sensitivity analysis, is presented in the eAppendix in Supplement 1.

### Statistical Analysis

#### Retrospective Analysis

We evaluated the association of CCT target coverage with child mortality and hospitalization during the years 2000 to 2019 using negative binomial multivariable regression models with fixed-effects specifications, which are consolidated methods for impact evaluations with aggregate-level panel data of hospitalization and mortality rates.^9,12,13,15,21,22^ The fixed-effects models include a term to control for characteristics of the unit of analysis that are approximately constant during the study period and that have not been included in the model as confounding variables, such as some geographic, historical, or sociocultural aspects of each municipality, whereas the negative binomial distribution is used to deal with the overdispersion of mortality data in the municipalities.^23^ We performed several sensitivity analyses (eAppendix in Supplement 1), including changing the categorization thresholds, adjusting model specifications, testing the models with municipalities with inadequate quality of CRVS, applying falsification/placebo test, performing a triangulation analysis^24^ using difference-in-difference with propensity score matching,^21^ and evaluating the municipalities with low CCT coverage vs medium and high coverage in the years 2004 and 2019. The findings withstood all the sensitivity and triangulation analyses, showing robustness and high confidence in the results.^21,24^ We used Stata software, version 17.0 (StataCorp) for database processing and analysis. Data analysis was performed from September 2022 to February 2023.

#### Forecast Analyses and Future Scenarios

To forecast the impact of the economic crisis and the mitigation association of CCT coverage, we used validated municipal-level microsimulation models.^20,25,26,27^ This approach was performed in 2 stages. First, we created a synthetic cohort of all municipalities in Brazil, Ecuador, and Mexico for 2020 to 2030 by extrapolating and modeling the independent variables from the 2000 to 2019 data set. Second, we forecast the mortality rates from 2020 to 2030, introducing these independent variables in the same multivariate regression models used in the retrospective analyses and including the estimates of their association.

In the first stage, we simulated 3 scenarios of economic crisis (short, medium, and long) characterized by changes in poverty rates that were calculated using household surveys in Brazil, Ecuador, and Mexico and, thereafter, extrapolated as municipal-level poverty trends as in previous studies.^20,25^ These changes in poverty were compatible with the increase in poverty rates measured by household surveys starting from 2020.^28^ The policy response scenarios to address these crisis scenarios were changes in the CCT coverage, namely, mitigation, baseline, and fiscal austerity, and were developed based on functions and parameters from validated models of previous studies^20,25,26^ focused in Brazil (eAppendix in Supplement 1). Brazil has been used as reference for 3 main reason: (1) because of the existence of previous forecasting studies^20,25,26^; (2) because it represented most of the municipalities in Brazil, Ecuador, and Mexico; and (3) because it is the largest economy in Latin America and has profound political influence on the countries of the region. In the mitigation scenario, the CCT target coverage increases proportionally to the growth of individuals in poverty during the economic crisis, followed by a reduction after the estimated end of the crisis. The baseline scenario represents the situation whereby the current trends are maintained, gradually reducing the budget for all welfare states according to constitutional rules and published estimates^20,25,26^ and reducing the CCT coverage accordingly. In the fiscal austerity scenario, CCT coverage decreases proportionally to the reduction observed in government expenditure on specific sectors of the social protection during the previous years.^16^ We performed 10 000 Monte Carlo simulations for each outcome and scenario, allowing parameter values to vary in each simulation cycle according to their assumed underlying distribution. We made substantial efforts to ensure and enhance internal and external validity for all microsimulation models, and all modeling procedures are presented in the eAppendix in Supplement 1. R software, version 4.1.2 (R Foundation for Statistical Computing) was used for the forecasting analysis.

## Results

### Retrospective Analyses

Our analyses were based on 4882 municipalities in Brazil, Ecuador, and Mexico that met CRVS criteria (mean [SD] poverty rate, 22.9 [16.6]; mean [SD] Gini Index, 53.0 [7.7]; mean [SD] number of hospital beds per 1000 population, 2.2 [2.1]; and mean [SD] number of physicians per 1000 population, 1.0 [1.0]). From 2000 to 2019, mortality in Brazil, Ecuador, and Mexico decreased by 7.8% in children and 6.5% in infants, with the greatest reduction observed in Brazil, followed by Ecuador and Mexico. The hospitalization rates decreased by 3% in the same period, and the CCT target coverage increased by 76.8% in these Latin American countries (Table 1).

**Table 1.** Municipal Mortality Rates, CCT Coverage, and Demographic, Socioeconomic, and Health Care–Related Variables for 4882 Selected Municipalities of Brazil, Ecuador, and Mexico

Variable	Mean (SD)		Change from 2000 to 2019, %
	2000	2019	
Mortality rate for children aged <5 years (per 1000 live births)			
Overall	22.2 (190.8)	14.4 (6.1)	−7.83
Neonatal mortality	12.2 (6.6)	8.3 (4.3)	−3.90
Postneonatal mortality	6.8 (5.3)	4.0 (2.9)	−2.79
Infant mortality	17.6 (11.1)	11.1 (5.5)	−6.46
Toddler mortality	3.2 (2.8)	2.1 (2.1)	−1.08
Hospitalization rate for children aged <5 y (per 1000 live births)	446.2 (1161.0)	443.5 (1088.0)	−2.76
CCT coverage of the target population, %	9.3 (23.4)	86.0 (25.2)	76.75
Other covariates			
Poverty rate, %	22.9 (16.6)	12.4 (12.3)	−10.59
Individuals >15 years with illiteracy, %	10.2 (8.8)	4.8 (5.1)	−5.40
Gini Index	53.0 (7.7)	47.9 (10.3)	−5.10
Piped water, %	81.3 (19.6)	89.1 (15.1)	7.80
Individuals living in households with adequate sanitation, %	33.4 (34.8)	48.2 (36.1)	14.77
Hospital bed rate per 1000 population, %	2.2 (2.1)	1.7 (1.4)	−0.47
Rate of physicians per 1000 population, %	1.0 (1.0)	1.6 (1.5)	0.58

Abbreviation: CCT, conditional cash transfer.

The age-stratified models (Table 2) show the associations between the CCT coverage levels and mortality rates in children, adjusted by the full set of covariates. The CCT program presented a significant dose-response association with decreases in mortality for all age groups in children younger than 5 years and the strongest association with postneonatal mortality. In the adjusted model of child mortality rates, a statistically significant reduction of 12% (rate ratio [RR], 0.88; 95% CI, 0.87-0.89) was associated with an intermediate level of coverage, 16% (RR, 0.84; 95% CI, 0.83-0.85) with a high level of coverage, and 24% (RR, 0.76; 95% CI, 0.75-0.76) with a consolidated level of coverage. Comparing the association of the CCT program with neonatal, postneonatal, infant, and toddler mortality, the greatest reductions were found in postneonatal mortality rates, with decreases of 15% (RR, 0.85; 95% CI, 0.84-0.86) associated with an intermediate level of coverage, 21% (RR, 0.79; 95% CI, 0.78-0.80) with a high level of coverage, and 31% (RR, 0.69; 95% CI, 0.68-0.70) with a consolidated level of coverage. The smallest association was observed with infant and neonatal mortality, reaching a maximum reduction of 7% and 10%, respectively. On the basis of these models, we estimated how many deaths in children have been avoided in Brazil, Ecuador, and Mexico during the last 2 decades (2000-2019) because of the implementation of CCT programs, comparing the real scenario with an alternative scenario in which all independent variables had real trends and values, except for the coverage of all CCT programs that was kept null during the study period. According to this scenario’s comparison, the number of avoided child deaths attributable to CCT program implementation during the last 2 decades had been 738 919 (95% CI, 695 641-782 104) (eAppendix in Supplement 1).

**Table 2.** Rate Ratios From the Fixed-Effects Negative Binomial Models by Age Groups for the Association Between Mortality Rates and CCT Coverage^a^

Variable	Rate ratio (95% CI)				
	Child (aged <5 y) (4882 municipalities and 95 281 observations)	Toddler (aged 1-4 y) (4662 municipalities and 87 090 observations)	Infant (aged <1 y) (4881 municipalities and 95 242 observations)	Postneonatal (28 d to 1 y) (4835 municipalities and 94 452 observations)	Neonatal (0-28 d) (4879 municipalities and 95 130 observations)
CCT target population coverage					
Low (0%-29.9%)	1 [Reference]	1 [Reference]	1 [Reference]	1 [Reference]	1 [Reference]
Intermediate (30.0%-69.9%)	0.88^b^ (0.87-0.89)	0.89^b^ (0.87-0.90)	0.93^b^ (0.92-0.93)	0.85^b^ (0.84-0.86)	0.90^b^ (0.89-0.91)
High (70.0%-99.9%)	0.84^b^ (0.83-0.85)	0.83^b^ (0.81-0.84)	0.88^b^ (0.87-0.89)	0.79^b^ (0.78-0.80)	0.87^b^ (0.86-0.88)
Consolidated (100%)	0.76^b^ (0.75-0.76)	0.74^b^ (0.73-0.76)	0.73^b^ (0.72-0.73)	0.69^b^ (0.68-0.70)	0.79^b^ (0.79-0.80)
Control variables					
Poverty rate	1.07^b^ (1.06-1.08)	1.11^b^ (1.09-1.14)	1.11^b^ (1.10-1.12)	1.01 (0.99-1.02)	1.10^b^ (1.09-1.12)
Individuals aged >15 y with illiteracy	1.05^b^ (1.04-1.06)	1.07^b^ (1.05-1.09)	1.07^b^ (1.06-1.08)	1.06^b^ (1.04-1.08)	1.03^b^ (1.02-1.05)
Gini Index	1.07^b^ (1.06-1.08)	1.03^b^ (1.01-1.05)	1.00^c^ (0.98-1.00)	1.09^b^ (1.07-1.10)	1.07^b^ (1.06-1.08)
Piped water	0.98^b^ (0.97-0.99)	0.97^c^ (0.95-1.00)	0.97^b^ (0.96-0.99)	0.93^b^ (0.91-0.94)	1.01^c^ (1.00-1.03)
Individuals living in households with adequate sanitation	0.91^b^ (0.90-0.92)	0.91^b^ (0.89-0.93)	0.97^b^ (0.96-0.98)	0.88^b^ (0.87-0.90)	0.93^b^ (0.92-0.94)
Hospital bed rate per 1000 population	1.01 (1.00-1.02)	1.00 (0.98-1.03)	0.96^b^ (0.95-0.97)	1.01 (0.99-1.03)	1.00 (0.99-1.02)
Rate of physicians per 1000 population	0.99^c^ (0.98-1.00)	0.95^b^ (0.94-0.97)	0.95^b^ (0.94-0.96)	0.97^b^ (0.96-0.99)	1.09 (1.00-1.02)

Abbreviation: CCT, conditional cash transfer.

^a^ Time shocks are controls for specific years of economic crisis (2008, 2013, and 2015) and for specific years of changes in CCT programs (2003, 2004, and 2014). Other time controls, including continuous-time and binary variables for other years, are in the eAppendix in Supplement 1.

^b^ Significant at *P* = .01.

^c^ Significant at *P* = .05.

Table 3 shows the adjusted associations between the CCT program coverage, hospitalization rates in those younger than 5 years, and mortality rates in those younger than 5 years for relevant causes of death. All poverty-related causes of child death had strong and statistically significant associations with CCT coverage, with a clear dose-response association. Consolidated levels of CCT coverage were associated with a 9% (RR, 0.91; 95% CI, 0.91-0.91) reduction in hospitalizations. Regarding the specific causes of death, we found the strongest association of the CCT coverage with mortality among those younger than 5 years resulting from malnutrition (67%; RR, 0.33; 95% CI, 0.31-0.35) and HIV/AIDS (68%; RR, 0.32; 95% CI, 0.28-0.37), whereas a smaller association was observed with lower respiratory tract infections (34%; RR, 0.66; 95% CI, 0.65-0.68), tuberculosis (38%; RR, 0.62; 95% CI, 0.48-0.79), and malaria (24%; RR, 0.76; 95% CI, 0.63-0.93). No statistically significant associations were found in mortality rates for external causes, used as control (eAppendix in Supplement 1). Models weighted for the municipal population have also shown very similar results (eAppendix in Supplement 1). All sensitivity analyses confirmed the robustness of the findings, and all triangulation analyses showed a high degree of confidence in the impact estimation (eAppendix in Supplement 1). A comparative analysis of the 3 different countries showed a stronger CCT association with child mortality outcomes in Brazil and Ecuador and lower associations in Mexico (eAppendix in Supplement 1).

**Table 3.** Fixed-Effect Negative Binomial Models for the Association Between Mortality Rate in Those Younger Than 5 Years by Specific Causes of Deaths Related to Poverty and CCT Coverage^a^

CCT target population coverage	Morbidity in children aged <5 y (4882 municipalities and 94 309 observations)	Rate ratio (95% CI)					
		Diarrheal diseases (3528 municipalities and 65 746 observations)	Malnutrition (3011 municipalities and 56 830 observations)	Tuberculosis (380 municipalities and 7453 observations)	Lower respiratory tract infection (3947 municipalities and 71 250 observations)	Malaria (526 municipalities and 10 290 observations)	HIV/AIDS (807 municipalities and 15 861 observations)
Low (0%-29.9%)	1 [Reference]	1 [Reference]	1 [Reference]	1 [Reference]	1 [Reference]	1 [Reference]	1 [Reference]
Intermediate (30.0%-69.9%)	0.94^b^ (0.94-0.94)	0.74^b^ (0.71-0.77)	0.64^b^ (0.61-0.68)	0.92 (0.70-1.21)	0.85^b^ (0.82-0.88)	0.91 (0.69-1.21)	0.69^b^ (0.59-0.81)
High (70.0%-99.9%)	0.93^b^ (0.93-0.93)	0.64^b^ (0.61-0.66)	0.50^b^ (0.48-0.53)	0.66^b^ (0.49-0.90)	0.79^b^ (0.76-0.82)	0.81^c^ (0.64-1.03)	0.60^b^ (0.50-0.71)
Consolidated (100%)	0.91^b^ (0.91-0.91)	0.41^b^ (0.40-0.43)	0.33^b^ (0.31-0.35)	0.62^b^ (0.48-0.79)	0.66^b^ (0.65-0.68)	0.76^b^ (0.63-0.93)	0.32^b^ (0.28-0.37)

Abbreviation: CCT, conditional cash transfer.

^a^ Time shocks are controls for specific years of economic crisis (2008, 2013, and 2015) and for specific years of changes in CCT programs (2003, 2004, and 2014). Other time controls, including continuous-time and binary variables for other years, are in the eAppendix in Supplement 1.

^b^ Significant at *P* = .01.

^c^ Significant at *P* = .10.

### Forecast Analyses

The Figure shows the trends in mortality rates for those younger than 5 years until 2030 according to the 3 potential economic crisis scenarios and the 3 alternative policy responses in terms of CCT coverage: mitigation, baseline, and fiscal austerity. In all economic crisis scenarios, the maintenance of social protection would mitigate the association with poverty increases and would cause decreasing trends in mortality rates for those younger than 5 years. On the contrary, the baseline and fiscal austerity scenarios would cause an initial increase in mortality rates for those younger than 5 years, followed by reductions occurring after the poverty peak and the end of the crisis. In all economic crisis scenarios, there would be an important difference between the trends in mortality rates for those younger than 5 years in the mitigation scenario vs the baseline and fiscal austerity scenarios. Table 4 gives the comparison analyses between scenarios in case of medium economic crisis: in 2030, the RR for mortality in those younger than 5 years between the maintenance and baseline scenarios would be 0.88 (95% CI, 0.86-0.91); that is, this mortality rate would be 12% lower if the policy of mitigation were implemented compared with a baseline implementation, avoiding 114 513 (95% CI, 93 846-135 896) deaths in those younger than 5 years in the period 2020 to 2030. The RR between the mitigation and the fiscal austerity scenarios would be 0.83 (95% CI, 0.80-0.85), and in this comparison, the policy of maintenance would avert 153 601 (95% CI, 127 441-180 600) deaths. The comparisons in case of other economic crisis scenarios are reported in the eAppendix in Supplement 1.

**Figure.**
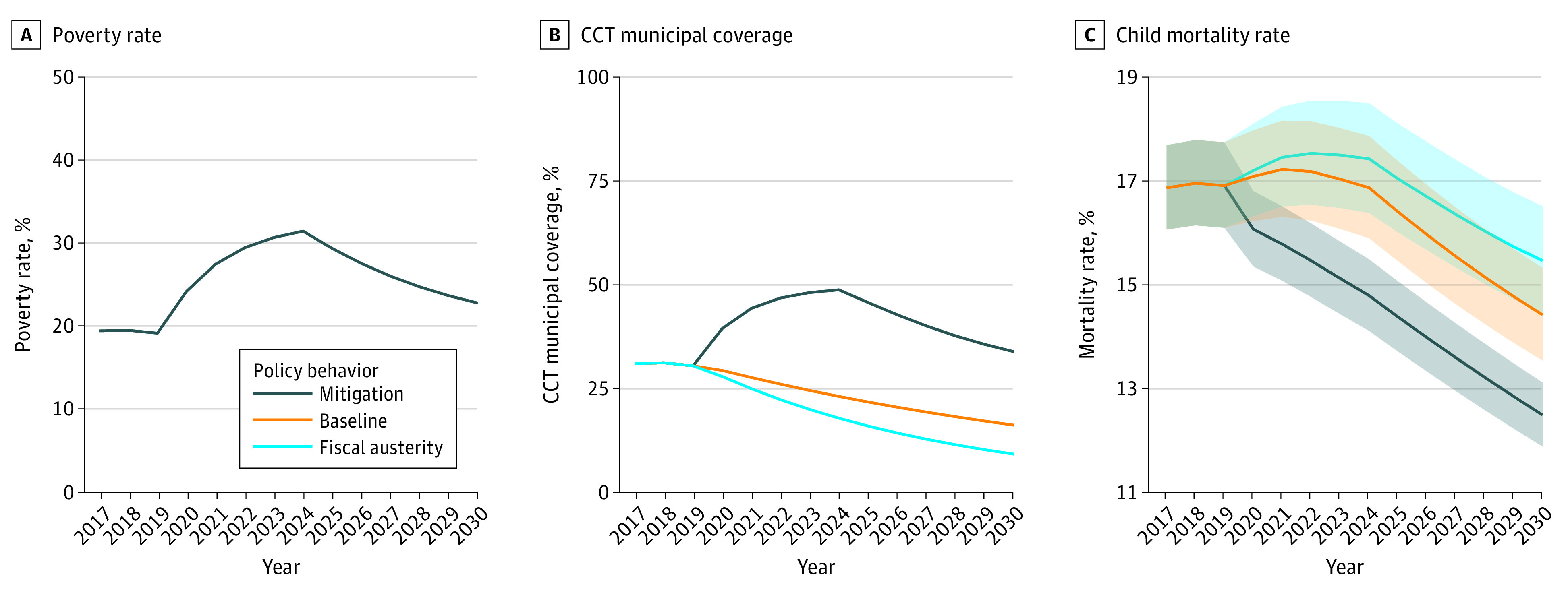
Potential Scenarios of Economic Crisis and Poverty, Alternative Responses of Conditional Cash Transfer (CCT) Program Coverage, and Related Child Mortality Rate Predictions for Brazil, Ecuador, and Mexico Up to 2030

**Table 4.** Rate Ratios From a Comparison of the Mitigation, Baseline, and Austerity Forecast Scenarios, 2020 to 2030

Year	Mortality, rate ratio (95% CI)	
	Mitigation vs baseline	Mitigation vs fiscal austerity
Child mortality		
2020	0.95 (0.94-0.96)	0.94 (0.93-0.99)
2025	0.89 (0.86-0.92)	0.86 (0.83-0.86)
2030	0.88 (0.86-0.91)	0.83 (0.80-0.85)
Avoidable deaths, No. (95% CI)	114 513 (93 846-135 896)	153 601 (127 441-180 600)

## Discussion

Our study shows that CCT programs were significantly associated with reduction in child hospitalization and mortality rates in Latin America during the last 2 decades, with stronger associations with postneonatal mortality and poverty-related causes. We also found that the implementation of these CCT programs may have prevented 738 919 child deaths between 2000 and 2019 in Brazil, Ecuador, and Mexico. Moreover, we showed that increased coverage of CCT programs—as a mitigation strategy for the current economic crisis—could avert 153 601 deaths in those younger than 5 years by 2030 compared with a response based on fiscal austerity measures. To the best of our knowledge, this is the largest and most comprehensive CCT evaluation study to perform a multicountry evaluation of the impact of CCT programs in Latin America to cover 2 decades and to use the resulting estimates to forecast the programs’ impact as mitigation policies during the current global economic crisis.

A comprehensive description of the design of the mechanisms that could explain the large impact of each CCT program in Brazil, Ecuador, and Mexico is provided in the eAppendix in Supplement 1. Conditional cash transfer programs can affect child mortality and morbidity through the income effect and conditionality effect, that is, by transferring direct income to the beneficiary families, improving families’ nutrition and living conditions, and conditioning the income transfer to the use of basic health services for child and maternal health.^10,11^

Previous studies focused on specific countries have shown that CCTs could reduce child and maternal mortality^9,10,11,12^ and, in adults, the burden of poverty-related diseases, such as leprosy, tuberculosis, and HIV/AIDS.^13,14,15^ In our study, we found a stronger association of CCTs with postneonatal and toddler mortality, which are more related to socioeconomic conditions,^11,18^ compared with the neonatal period, during which mortality depends more on health care infrastructure and assistance during and after delivery. However, the important association of CCTs with neonatal mortality can be explained by CCT health-related conditionalities, in particular the attendance of prenatal visits.^9,10,11^

We also found particularly strong associations with causes of death related to poverty, such as malnutrition and diarrhea. Although this type of CCT impact was expected, the magnitude was greater than previously reported,^9^ probably because of the ability of the CCT coverage indicator and the long study period to fully appreciate CCT effects. A strong association was also encountered with child mortality from tuberculosis and lower respiratory tract infections, whereas the unexpectedly strong association with mortality from HIV/AIDS in those younger than 5 years could be explained by the reduction of vertical transmission due to the detection of the virus in the mother‘s blood during prenatal visits.^10,29^ The influence on child mortality from malaria could also be explained by the association of this parasitic disease with poverty and socioeconomic vulnerability.^30^

### Strengths and Limitations

The main strengths of our study are the large range of sensitivity analyses performed, which confirmed the robustness of the findings, and the inclusion of triangulation analyses, which demonstrate a high degree of confidence in the results of the impact evaluation, conferring robustness also to the forecasted scenarios that have been based on validated models.

This study also has several limitations, which have been extensively addressed and potentially mitigated. The first is the choice to work only with municipalities with adequate quality of CRVS, which has strengthened the internal validity of the study but could also have reduced the generalizability of the results. Although the exclusion of municipalities with an inadequate level of vital information could have reduced the external validity of the findings, it was an essential factor for strengthening the internal validity of the study and reducing any possible bias caused by changes in the quality of the death notification system, mainly reduction of subnotifications, during the study period. However, these quality criteria are commonly used in similar studies in Latin American countries,^9,12,15^ and our sensitivity analyses showed that the main results were maintained also when all municipalities were considered. The second limitation is the uncertainty of the forecasted scenarios and future mortality rates in those younger than 5 years attributable to the high socioeconomic and health volatility in Latin American countries in the middle and long term. Although unpredictable events can always occur, the focus of our evaluation is on the comparison of alternative policy implementation scenarios rather than on forecasting exact morality rates up to 2030, and the mortality rate ratios in those younger than 5 years between these policy scenarios remain consistent independent from unexpected events that could occur. A limitation may be the uncertainty regarding the forecast of the economic crisis: even if all current trends are indicating a global long-term increase in poverty rates,^1,2,3^ similar to what happened in Brazil in the 2015 to 2016 economic crisis,^28^ the economic and political situation is still uncertain. For that reason, we forecasted different economic crisis scenarios, which continued to display coherent and consistent outcomes, despite differences in the scenario magnitude.

## Conclusions

This cohort study found that CCT programs are associated with significant reductions in child morbidity and mortality in Latin America, in particular for poverty-related diseases, such as malnutrition, diarrhea, tuberculosis, and HIV/AIDS. Moreover, all our forecasted scenarios suggest that a prompt coverage expansion of CCT programs to protect those newly in poverty could represent an effective policy to mitigate the adverse health impact of the current economic crises in low- and middle-income countries.

## Notes

Supplement 1. **eAppendix.** Supplementary Information

Supplement 2.
Data Sharing Statement
