# Supplementary material for: Evaluation and Forecasting Analysis of the Association of Conditional Cash Transfer With Child Mortality in Latin America, 2000-2030
Source: JAMA Netw Open. 2023 Jul 14;6(7):e2323489. doi: 10.1001/jamanetworkopen.2023.23489 (PMC10349336; doi:10.1001/jamanetworkopen.2023.23489)
Supplement: Supplement 1. — eAppendix. Supplementary Information [file jamanetwopen-e2323489-s001.pdf]

Supplementary Online Content

Cavalcanti DM, Ordoñez JA, Aransiola T, et al. Evaluation and forecasting analysis of the association of conditional cash transfer with child mortality in Latin America, 2000-2030. *JAMA Netw Open*. 2023;6(7):e2323489. doi:10.1001/jamanetworkopen.2023.23489

eAppendix. Supplementary Information

This supplementary material has been provided by the authors to give readers additional information about their work.

© 2023 Cavalcanti DM et al. *JAMA Network Open.*

TABLE OF CONTENTS

PART I – SUMMARY OF SOCIAL PROGRAMS, DATA SOURCES AND GENERAL METHODOLOGY 1
1. Background – Cash Transference Programs in BEM countries 1
  1.1. Bolsa Familia Program (BFP) and Auxílio Brasil Program (ABP) 1
  1.2. Bono de Desarrollo Humano Program (BDHP) 1
  1.3. Prospera Program 1
  1.4. Mechanism of the effect of CCT programs on health outcomes 2
2. Dataset 3
  2.1. Data sources 3
  2.2. Interpolation and extrapolation method 3
  2.3. Some details about the poverty rate 5
  2.4. Quality of Vital Statistics Method 8
  2.4.1. Discussion and Results – quality of CRVS method 8

PART II – RETROSPECTIVE ANALYSIS 10
3. Empirical methods 10
  3.1. Negative binomial regression – Fixed Effects 10
4. Results 10
  4.1. Descriptive Analysis 10
  4.2. Retrospective Analysis 15
  4.3. Triangulation – Difference-in-difference with propensity score matching 15
  4.4. Fit and sensitivity tests 18
  4.5. Deaths averted by CCT programs during 2000-19 32

PART III – FORECASTING ANALYSIS 33
5. Description of the forecasting methodology 33
6. Purpose of the forecasting and its applications 33
7. Inputs, outputs, and other parameters 33
  7.1. Scenarios of poverty and coverage of CCT programs 33
8. Prediction methodology 36
  8.1. External validation of each model 36
9. Sensitivity analysis 36
10. Main Limitations 37

REFERENCES 39

© 2023 Cavalcanti DM et al. *JAMA Network Open.*

PART I – SUMMARY OF SOCIAL PROGRAMS, DATA SOURCES AND GENERAL METHODOLOGY

1. Background – Cash Transference Programs in BEM countries

1.1. Bolsa Familia Program (BFP) and Auxílio Brasil Program (ABP)

The Brazilian CCT program, branded as Bolsa Família Programme (BFP), established in 2004, is an important socio-economic intervention that aims to attenuate the effects of absolute poverty through a minimum cash transfer for beneficiary families, and to break the intergenerational cycle of poverty through investment in education and health conditionalities.[1]

This important socio-economic intervention was established in 2004 by Law № 10,836, of January 9, 2004, with the last monetary restatement modified by Decree № 9,396, of May 30, 2018, in which households are eligible for the program if their per capita income is equal to or less than R$89.00 (approximately US$19 at current 2022 prices) or if they are poor families with income up to R$ 178.00 (approximately US$ 38 at current 2022 prices) and one member is a child up to 17 years old or a pregnant woman (or a woman who just gave birth).[2]

In the health area, conditionalities concern the monitoring of vaccination and nutritional surveillance of children, as well as prenatal care of pregnant women and the puerperium, and should be attended at the Family Health Strategy (*Estratégia de Saúde da Família*, ESF, in Portuguese) units. Some studies have shown the effect of BFP on child morbidity and mortality,[3] and on other health outcomes associated with poverty, such as tuberculosis, leprosy, [1] mortality from suicides, and homicides.[4]

The BFP is one of the largest CCT in the world with more than 13.9 million families benefiting throughout Brazil, and was recently remodeled and called Axílio Brasil Program (ABP) by Law № 14,284, of December 29, 2021. Basically, this new program maintains the conditionalities and general structure of the BFP, but it increases the coverage and the value transferred through changes in the criteria of poverty and extreme poverty; where families earning up to R$105.00 per capita (approximately US$22 at current 2022 prices) and up to R$210.00 (almost US$45 at current 2022 prices) are considered extremely poor and poor, respectively. No studies were found that directly evaluate the ABP, however studies that simulated scenarios of increased coverage of BFP show improvements in health outcomes.[3]

1.2. Bono de Desarrollo Humano Program (BDHP)

The largest CCT programme in Ecuador is the Bono de Desarrollo Humano (BDH) that provided a cash transfer of US$50 per month to low income mothers below the poverty line according to the Social Registry. The required behaviours included both attendance by both the mother and children at preventive health check-ups and requiring a minimum percentage of attendance at school for school-age children. The aim of the programme is to guarantee a minimum level of consumption for families and to contribute to the reduction of chronic malnutrition and preventable diseases for children under the age of five. A study performed by the proponents of the project showed that the implementation of the BDH from 2009- 2014 was associated with a reduction in under-5 mortality rate from poverty-related causes such as malnutrition and lower respiratory infections at the county level.[5] Processes of social mobility promoted by the government in the last decade and the modification of the target population to people living in extreme poverty (due to economic crises caused by the lower oil prices), caused the number of beneficiaries to decrease by 56.7% between 2013 and 2014 without an impact evaluation of these changes.[5]

1.3. Prospera Program

One of the oldest and important CCT policies worldwide is the Mexico's programme known as Prospera (formerly Oportunidades or Progresa),[6] seeks to improve the provision and quality of basic social services (health, nutrition, and education), largely among the most economically and socially disadvantaged populations.[7]

© 2023 Cavalcanti DM et al. *JAMA Network Open.*

Once the families have been accepted in the program based on a multidimensional eligibility criterion of the households; they must comply with their health and education - related co - responsibilities, which compliance is checked once every two months, and this initiative involved a coordinated effort between the social and development division and the health sector.[8]

1.4. Mechanism of the effect of CCT programs on health outcomes

There are several mechanisms through which the CCT program affect health outcomes. First, the CCT conditions beneficiaries to a minimum usage of health services for child and maternal health, i.e., the conditionality effect.[3,9] Second, the income transferred to poor and extremely poor families improves the nutrition and living conditions of these families, i.e, the income effect.[1] Third, long-term exposure to the health conditionality of the CCT promote behavior changes and adherence of beneficiary families towards health care.[10]

eFigure 1. Mechanisms linking the Conditional Cash Transference Program to health outcomes.

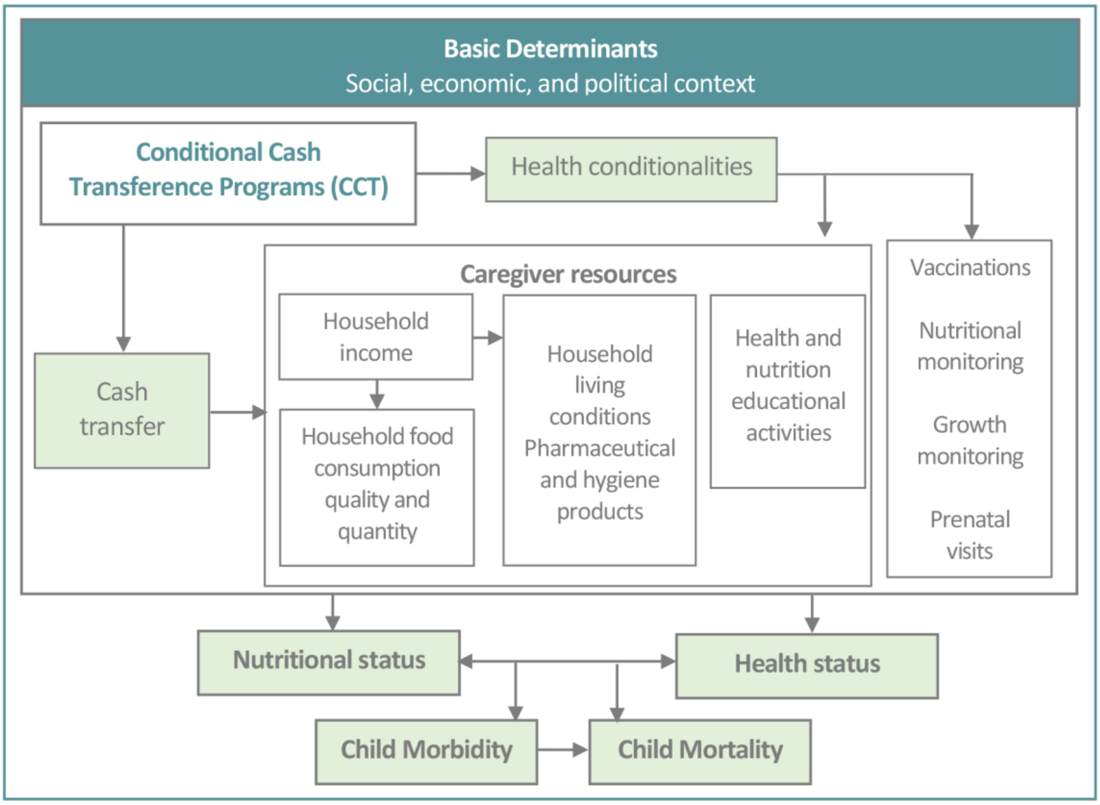

© 2023 Cavalcanti DM et al. *JAMA Network Open.*

2. Dataset

2.1. Data sources

The data used in this study were obtained from various governmental platforms detailed in eTable 1. All the variables used in this study are aggregated to the municipal level. However, the data for some variables were not available for specific years and municipalities, therefore we performed a exponential decay method for interpolation, as detailed in Section 2.2. of this supplementary document.

eTable 1. Data sources and description of variables

VARIABLE	BRAZIL				ECUADOR				MEXICO			
	Years	Units of analysis	Source	Link	Years	Units of analysis	Source	Link	Years	Units of analysis	Source	Link
Mortality and Morbidity	2000 to 2019	Individual	DATASUS - SIM	LINK 1	2000 to 2019	Individual	INEC	LINK 14	2000 to 2020	Individual	INEGI	LINK 15
Population estimates	2000 to 2019	Municipal	IBGE – Census	LINK 2	2000 to 2019	Municipal	INEC	LINK 14	2000 to 2020	Municipal	CONAPO	LINK 16
Livebirth	2000 to 2019	Municipal	DATASUS - SINASC	LINK 3	2000 to 2019	Municipal	INEC	LINK 14	2000 to 2019	Municipal	DGIS	LINK 17
Socioeconomic variables	2000 and 2010, 2001-2019	State and Municipal	Census; -PNAD; and – PNADC (IBGE)	LINK 4	2001 to 2010	Municipal	INEC	LINK 14	2000, 2005, 2010, and 2020	Municipal	CONE-VAL	LINK 18
CCT coverage	2004 to 2019	Municipal	MDS	LINK 5	2009 to 2020	Municipal	NSI, MIES	LINK 14.1	2000 to 2019	Municipal	DAGM	LINK 18.1
Hospital bed rate (beds per 1,000 inhabitants)	2000 to 2019	Municipal	DATASUS - CNES	LINK 6	2004 to 2020	Municipal	INEC	LINK 14	2001 to 2020	Municipal	DGIS	LINK 19
Doctor rate (Physicians per 1,000 inhabitants)	2000 to 2019	Municipal	DATASUS - CNES	LINK 7	2000 to 2017	Municipal	INEC	LINK 14	2001 to 2020	Municipal	DGIS	LINK 19

Note: | **BRAZIL SOURCES**: DATASUS - Department of Informatics of the Unified Health System (Departamento de Informática do Sistema Único de Saúde); SIM - Mortality Information System (Sistema de Informações sobre Mortalidade); DAB - Department of Primary Care (Departamento de Atenção Básica); CNES - National Register of Health Establishments (Cadastro Nacional de Estabelecimentos de Saúde; SINACS - Live Birth Information System (Sistema de Informações sobre Nascidos Vivos); IBGE - The Brazilian Institute of Geography and Statistics (Instituto Brasileiro de Geografia e Estatística); MDS - The Ministry of Social Development and Fight against Hunger (Ministério do Desenvolvimento Social e Combate à Fome); PNAD - (Pesquisa Nacional por Amostra de Domicílios); PNADC - Continuous PNAD Quarterly (Pesquisa Nacional por Amostra de Domicílios Contínua).| **ECUADOR SOURCES:** INEC - =National Statistics Institute (Instituto Nacional de Estadística); NSI - National System of Information; MIES – Ministry of Economic and Social Inclusion | **MEXICO SOURCES:** INEGI – National Institute of Statistic and Geography (Instituto Nacional de Estadística y Geografía); CONAPO – National Population Council (Consejo nACional de Población); CONEVAL – National Council for the Evaluation of Social Development Policy (Consejo Nacional de Evaluación de la Política de Desarrollo Social); DGIS – General Directorate of Health Information (Dirección General de Información en Salud); DAGM – Open Data - Government of Mexico (Datos Abiertos - Gobierno de México)

2.2. Interpolation and extrapolation method

Some of control variables are not available in total period of 2000-19. In these cases, we use the exponential decay method to extrapolate the variables available at least two points of time, and we drop municipalities with only one information.

A total of 893,214 values were generated for 7 control variables, in 8,103 municipalities over 20 years with this method. The interpolated variables were Illiteracy rate, household infrastructure (sewage and piped water), hospital bed rate, doctor rate and inequality and income variables (Gini index and poverty rate). Some of these variables were used as control variables for the models, none of the outcome

variables (mortality and morbidities) or exposure variables (CCT programs) were interpolated. In the end, it was observed that the interpolated/extrapolated variables improved the control and precision of the retrospective and predictive models.

eFigure 2. Gini index boxplot for selected municipalities in Brazil, Ecuador and Mexico, BEM, from the period 2000-19.

EACH COUNTRY – GINI INDEX

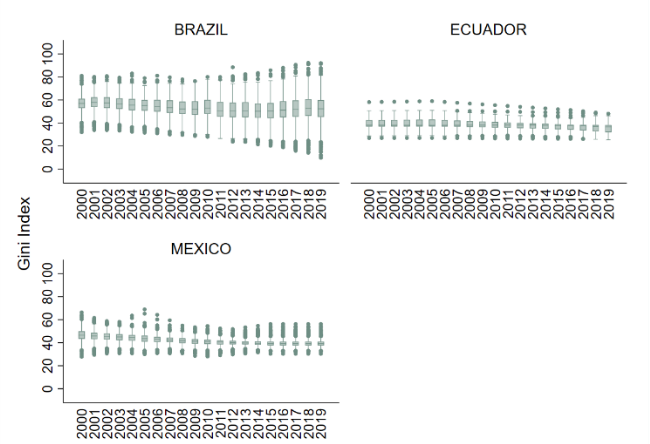

ALL COUNTRIES (BEM) – GINI INDEX

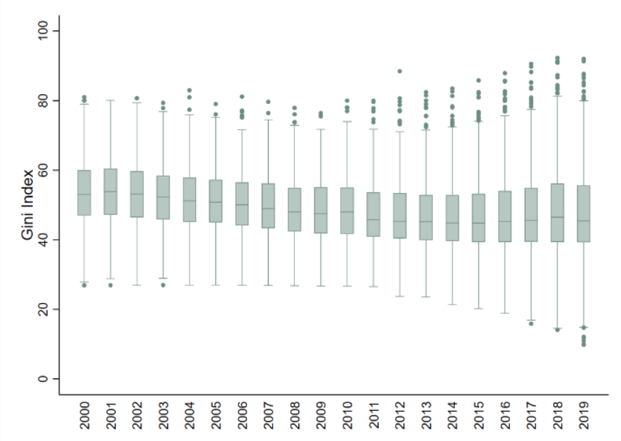

Source: Author's plot for 4,884 selected municipalities in Brazil, Ecuador, and Mexico, over 20 years (from 2000 to 2019).
Note: We selected municipalities with adequate quality of civil registration and vital statistics (CRVS). In Brazil, the gini index was available at the municipal level for the years 2000 and 2010, and the period 2001-09 and 2011-119 were extrapolated. Ecuador has the gini index available at the municipal level for the years 2005 and 2014, and we extrapolate the period 2000-04, and 2015-19. In Mexico, the gini index at the municipal level is available for the years 2000, 2002, and 2004-19, and we only extrapolate the years 2001 and 2003.

eFigure 3. Illiteracy rate boxplot for selected municipalities in Brazil, Ecuador and Mexico, BEM, from the period 2000-19.

EACH COUNTRY – ILLITERACY RATE

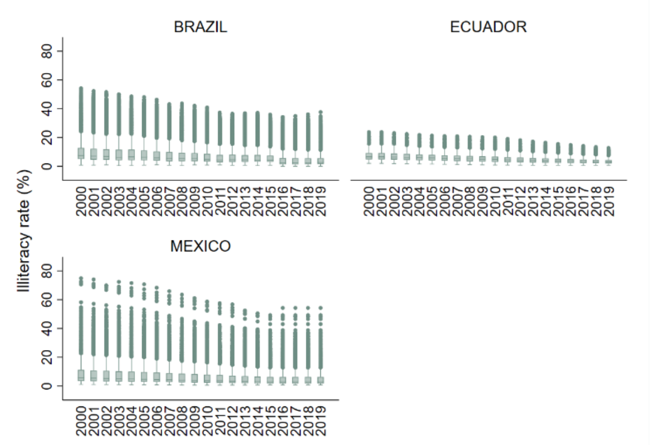

ALL COUNTRIES (BEM) – ILLITERACY RATE

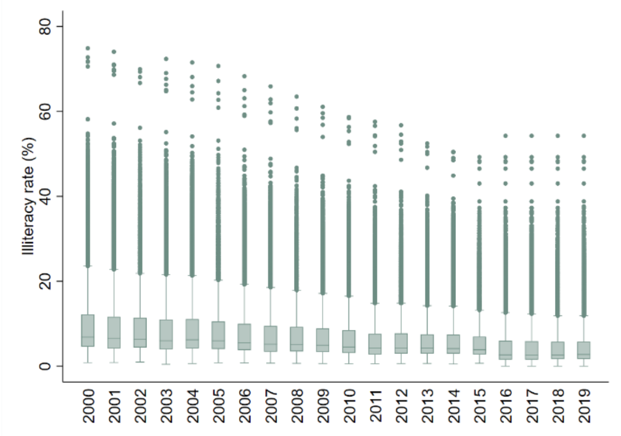

Source: Author's plot for 4,884 selected municipalities in Brazil, Ecuador, and Mexico, over 20 years (from 2000 to 2019).
Note: Illiteracy rate is the proportion of individuals older than 15 years who are illiterate. We selected municipalities with adequate quality of civil registration and vital statistics (CRVS). In Brazil, the illiteracy rate was available at the municipal level for the years 2000 and 2010, and the period 2001-09 and 2011-119 were extrapolated. Ecuador has the illiteracy rate available at the municipal level for the years 2001 and 2010, and we extrapolate the period 2002-09, 2011-19, and the year 2000. In Mexico, the gini index at the municipal level is available for the years 2000, 2005, 2010, 2015 and 2020, so we extrapolate the years 2001-04, 2006-09, 2011-14, and 2015-19.

© 2023 Cavalcanti DM et al. *JAMA Network Open.*

eFigure 4. Poverty rate boxplot for selected municipalities in Brazil, Ecuador and Mexico, BEM, from the period 2000-19.

EACH COUNTRY – POVERTY RATE

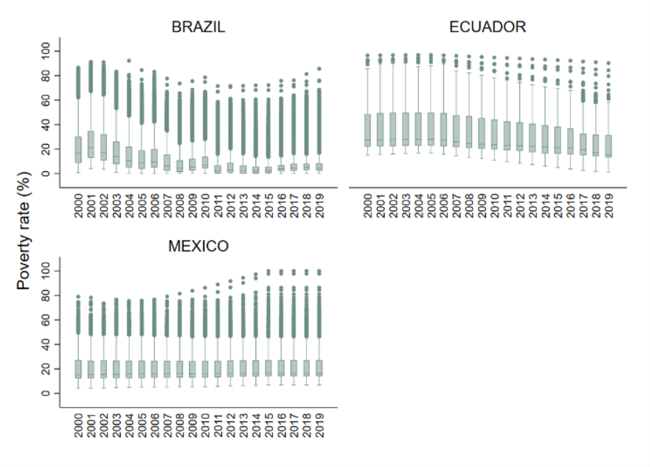

ALL COUNTRIES (BEM) – POVERTY RATE

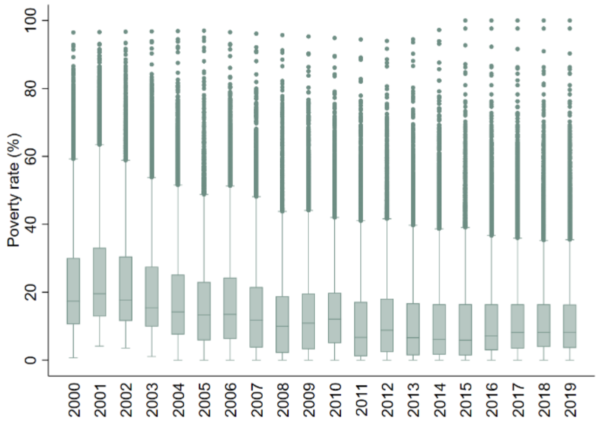

Source: Author's plot for 4,884 selected municipalities in Brazil, Ecuador, and Mexico, over 20 years (from 2000 to 2019).
Note: We selected municipalities with adequate quality of civil registration and vital statistics (CRVS). In Brazil, the poverty rate was available at the municipal level for the years 2000 and 2010, and the period 2001-09 and 2011-119 were extrapolated with microdata at state level. Ecuador has the poverty rate available at the municipal level for the years 2001 and 2010, and we extrapolate the period 2002-09, 2011-19, and the year 2000. In Mexico, the poverty rate at the municipal level is available for the years 2000, 2005, 2010, 2015 and 2020, so we extrapolate the years 2001-04, 2006-09, 2011-14, and 2015-19.

2.3. Some details about the poverty rate

We use poverty concepts according to the eligibility criteria of each CCT program in each BEM country. In the case of Mexico, since 1999 the CCT program (Progresa/ Oportunidad/ Prospera) use a multidimensional poverty criteria (called "*indice de marginación*") like a targeting mechanism for identify the eligibility of a family/household,[11] provided by CONAPO (National Population Council, "*Consejo Nacional de Población*"). We collected this information and used it as a poverty measure for the Mexico CCT program.

In Ecuador, the CCT program (*Bono de Desarrollo Humano*) also use a multidimensional poverty line, called Unsatisfied Basic Needs Index ("*Necesidades Básicas Insatisfechas*" – NBI),[12] provided by INEC (National Statistics Institute, "*Instituto Nacional de Estadística*"). We collected this information and used it as a poverty measure for the CCT program in Ecuador.

In Brazil a monetary poverty line is used to identify eligible families, originally being ½ of the minimum wage in 2003, and is updated by official decrees.[13] We use PNAD (National Household Sample Survey, "*Pesquisa Nacional por Amostra de Domicílios*") microdata to calculate how many people are below this *Bolsa Família* Program (BFP) poverty line, and thus obtain the BFP poverty rate.

Thus, we have different concepts of poverty, with different measures by country. However, the main objective of the poverty rate variable is to calculate the target coverage of the CCT programs (see session 4.1). We also used it as a control variable, and for that purpose we transformed this rate into a dummy variable, assuming a value equal to zero if the municipality is below the poverty median, and equal to 1 if the municipality is above the poverty median; where this median was calculated separately by country.

© 2023 Cavalcanti DM et al. *JAMA Network Open.*

eFigure 5. Piped water boxplot for selected municipalities in Brazil, Ecuador and Mexico, BEM, from the period 2000-19.

EACH COUNTRY – PIPED WATER

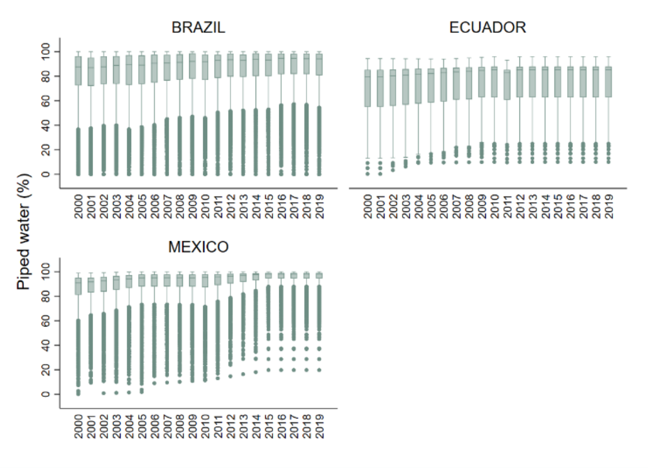

ALL COUNTRIES (BEM) – PIPED WATER

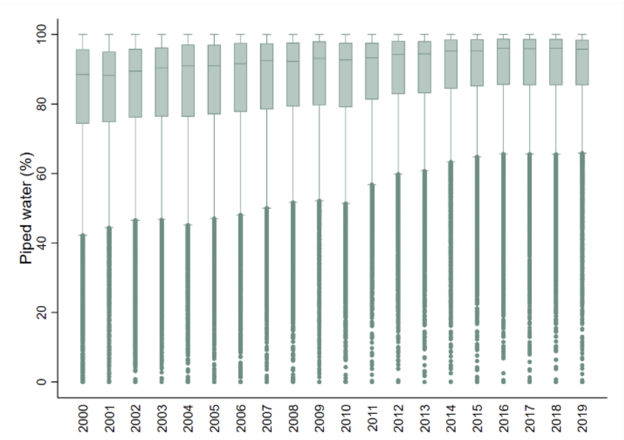

Source: Author's plot for 4,884 selected municipalities in Brazil, Ecuador, and Mexico, over 20 years (from 2000 to 2019).
Note: This variable refers is the proportion of individuals living in households with piped water. We selected municipalities with adequate quality of civil registration and vital statistics (CRVS). In Brazil, the piped water was available at the municipal level for the years 2000 and 2010, and the period 2001-09 and 2011-119 were extrapolated. Ecuador has the piped water coverage available at the municipal level for the years 2001 and 2010, and we extrapolate the period 2002-09, 2011-19, and the year 2000. In Mexico, the piped water at the municipal level is available for the years 2000, 2005, 2010, 2015 and 2020, so we extrapolate the years 2001-04, 2006-09, 2011-14, and 2015-19.

eFigure 6. Sewage boxplot for selected municipalities in Brazil, Ecuador and Mexico, BEM, from the period 2000-19.

EACH COUNTRY – SEWAGE

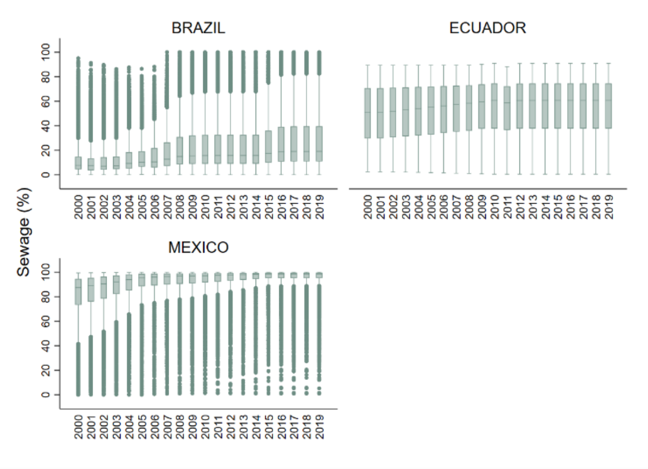

ALL COUNTRIES (BEM) – SEWAGE

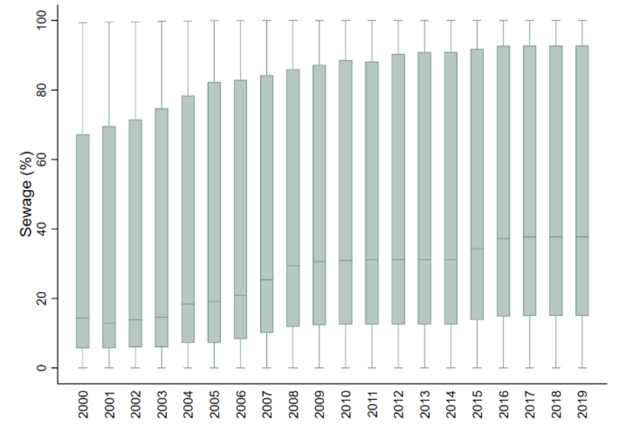

Source: Author's plot for 4,884 selected municipalities in Brazil, Ecuador, and Mexico, over 20 years (from 2000 to 2019).
Note: This variable refers is the proportion of individuals living in households with adequate sanitation. We selected municipalities with adequate quality of civil registration and vital statistics (CRVS). In Brazil, the sewage coverage was available at the municipal level for the years 2000 and 2010, and the period 2001-09 and 2011-119 were extrapolated. Ecuador has the sewage coverage available at the municipal level for the years 2001 and 2010, and we extrapolate the period 2002-09, 2011-19, and the year 2000. In Mexico, the sewage at the municipal level is available for the years 2000, 2005, 2010, 2015 and 2020, so we extrapolate the years 2001-04, 2006-09, 2011-14, and 2015-19.

eFigure 7. Physicians rate boxplot for selected municipalities in Brazil, Ecuador and Mexico, BEM, from the period 2000-19.

EACH COUNTRY – PHYSICIANS RATE

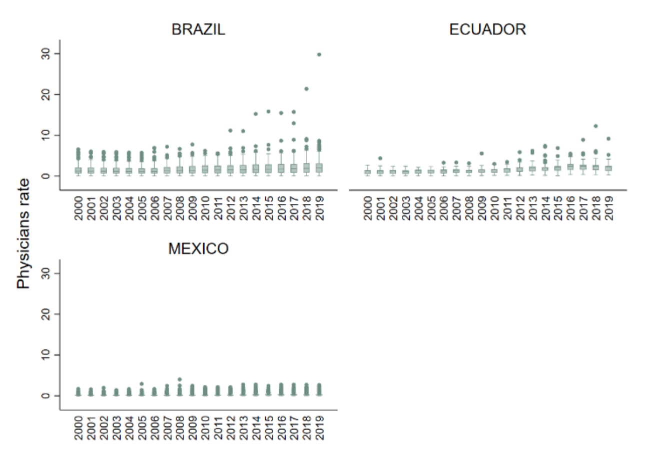

ALL COUNTRIES (BEM) – PHYSICIANS RATE

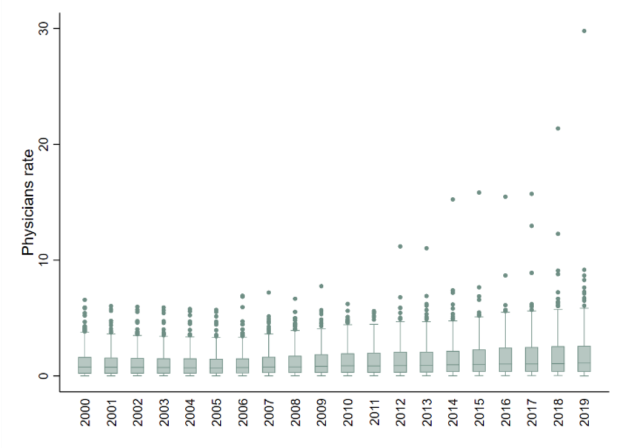

Source: Author's plot for 4,884 selected municipalities in Brazil, Ecuador, and Mexico, over 20 years (from 2000 to 2019).
Note: This variable refers to number of physicians per 1,000 inhabitants. We selected municipalities with adequate quality of civil registration and vital statistics (CRVS). In Brazil and Mexico, the physicians rate was available at the municipal level for the years 2000-19 (no need extrapolations). Ecuador has the physicians rate available at the municipal level for the years 2000-17, and we extrapolate the period 2018-19.

eFigure 8. Hospital beds rate boxplot for selected municipalities in Brazil, Ecuador and Mexico, BEM, from the period 2000-19.

EACH COUNTRY – HOSPITAL BEDS RATE

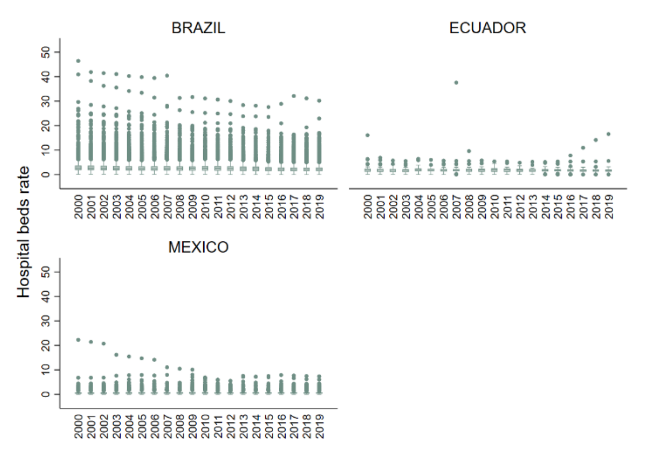

ALL COUNTRIES (BEM) – HOSPITAL BEDS RATE

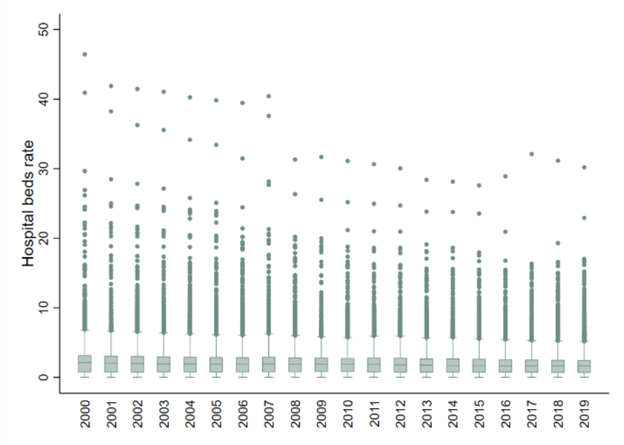

Source: Author's plot for 4,884 selected municipalities in Brazil, Ecuador, and Mexico, over 20 years (from 2000 to 2019).
Note: This variable refers to number of hospital beds per 1,000 inhabitants. We selected municipalities with adequate quality of civil registration and vital statistics (CRVS). In Brazil and Mexico, the hospital bed rate was available at the municipal level for the years 2000-19 (no need extrapolations). Ecuador has the hospital beds rate available at the municipal level for the years 2004-19, and we extrapolate the period 2000-03.

© 2023 Cavalcanti DM et al. *JAMA Network Open.*

2.4. Quality of Vital Statistics Method

The civil registration and vital statistics (CRVS), despite of being an important public health instrument for planning and evaluation, in most of the developing countries have still low quality and coverage.[14] In that regard, a study[15] involving 148 countries shows that the most of places with medium to low quality of CRVS during the period 1980-2012 were in the African, Asian Latin America regions, which reinforces the necessary care and attention of studies that work with CRVS in these regions. However, countries have been encouraged to stimulated by the need to monitor progress and accountability of CRVS, especially since 2015 for the Sustainable Development Goals (SDG).[14]

From a methodological point of view, the good quality of these CRVS is also crucial to guarantee the veracity of the results, especially in impact evaluation studies. Since our study uses municipal-level child death and livebirth data in LACs countries, it is essential to separate those municipalities with high and low quality from these CRVS.

To mitigate this, we followed a methodology[16] to calculate the level of quality of CRVS of municipalities, which has been widely used in previous studies in Latin America and Caribbean (LAC) countries.[5,17] This methodology used a validated multidimensional criterion based on five indicators: [1] relative mean deviation of the birth-rate; [2] ratio of reported-to-estimated livebirths; [3] age-standardized mortality rate; [4] relative mean deviation of the mortality rate; and [5] proportion of deaths with undetermined causes (Chapter XVIII, ICD-10).[16]

After calculating these indicators, we made a weighted average of them to obtain a final indicator, followed by the division by terciles of the distribution of each country separately, so that the municipalities within the last two terciles were considered to have good quality of CRVS, and the first tercile refer to municipalities with low quality of CRVS.

As the quality CRVS tends to improve in more recent periods, we chose to apply this methodology in the period from 2000 to 2012, as it is the beginning of the historical series worked on in this paper and, at the same time, captures the beginning of the implementation of CCT programs.

2.4.1. Discussion and Results – quality of CRVS method

After applying this method to all 8,103 municipalities in BEM with data available in 2000-19 period, we selected a subset of 4,884 municipalities that had adequate quality of CRVS, which coverage 60% of them, but representing 86% of the more than 340 million inhabitants of these 3 countries in 2002.

Additionally, the application of this method showed important socio-spatial inequalities: In Brazil, the proportion of adequate vital statistics was higher in the Center-South of the country and in the larger municipalities, and lower in many municipalities in the North of the country (Amazon region) and some municipalities in the Northeast. In Ecuador, the lowest results are located in the eastern region of these countries, especially in the Amazon region, while the municipalities with the highest QVI are from Andean regions. In Mexico, the best results are in the northern region of the country and the worst are in the southern region. The Web-Table 2 and Web-Figure 9 show these results.

eTable 2. Number of municipalities and population before and after filter by adequate CRVS.

COUNTRY	NUMBER OF MUNICIPALITIES			BY POPULATION		
	WITH DATA AVALIABLE DURIN 2000-19 PERIOD	FILTER BY ADEQUATE QUALITY OF CRVS (2000-02)	%	TOTAL	FILTER BY ADEQUATE QUALITY OF CRVS (2000-02)	%
Brazil	5,507	3,634	67%	178,135,381	155,200,666	87%
Ecuador	221	146	66%	13,232,884	11,202,804	85%
Mexico	2,375	1,104	47%	110,576,814	95,918,270	87%
**BEM**	**8,103**	**4,884**	**60%**	**301,945,079**	**262,321,740**	**86%**

Source: Author’s analysis of data from 2000-02 from SIM (DATASUS – Brazil), INEC (Ecuador), and INEGI (Mexico).

© 2023 Cavalcanti DM et al. *JAMA Network Open.*

eFigure 9. Municipalities according to the quality of vital information

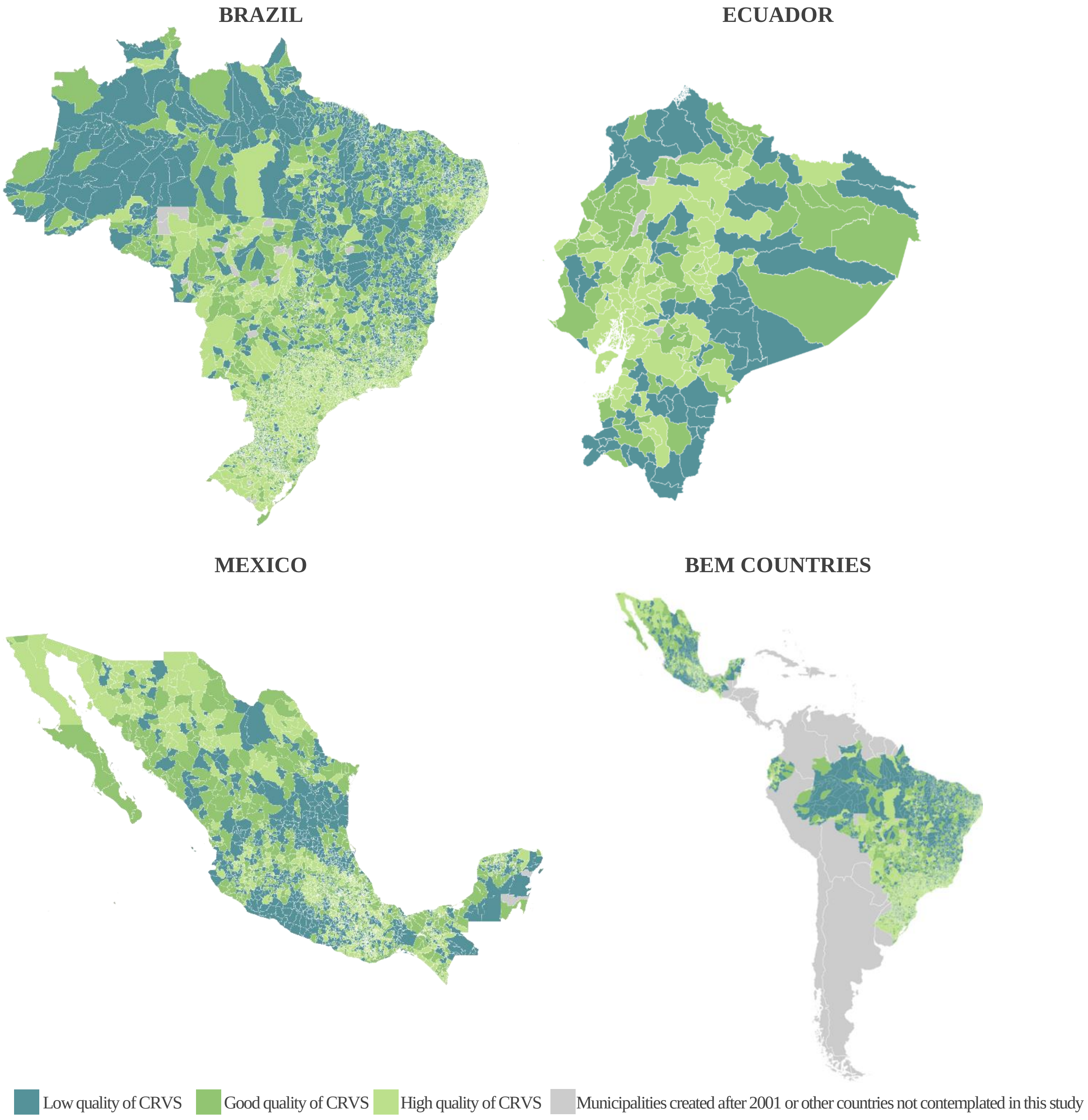

Source: Author's analysis of data from 2000-02 from SIM (DATASUS – Brazil), INEC (Ecuador), and INEGI (Mexico).

© 2023 Cavalcanti DM et al. *JAMA Network Open.*

PART II – RETROSPECTIVE ANALYSIS

3. Empirical methods

3.1. Negative binomial regression – Fixed Effects

We estimate Fixed Effect models using the negative binomial method to retrospectively evaluate and forecast the impact of each welfare social policy on health outcomes. The equation which describes the linear relationship between the health outcomes (mortality and hospitalization rates) and covariates is given by:

$$Log\, Y_{it} \;=\; \alpha_i \;+ \sum_{q=1}^{3} \beta_q CCT_{qit} + \sum_{s=4}^{9} \beta_s T_y + \sum_{k=10}^{16} \beta_k X_{kit} + u_{it}$$

where:

- t refers to the year, i refers to an individual municipality, and q are indexes representing each categories of CCT programs coverage.
- Y_{it} are the different welfare state variables (mortality and hospitalization rates for under-five age group) observed at the municipality i in year t.
- CCT_{qit} are the dummies representing the CCT coverage categories (4 groups) observed at the municipality i in year t with a coefficient of β_q; with q=0 for low coverage (0–29.9%), q=1 for intermediate (30–69.9%), q=2 to high (70–99.9%), and q=3 for consolidated coverage (>=100%).
- $T_y,$ are dummy variables representing previous crisis events with coefficients $\beta_4, \beta_9, \beta_{10}$ from the specific years y , with y=2003, 2004, 2008, 2013, 2014, 2015, respectively.
- X_{kit} represents different control covariates, each one with a coefficient of β_k (Poverty, Gini Index, illiteracy, doctors rates, hospital bed rate, Proportion of individuals living in households with inadequate sanitation, and Piped water).
- α_i is the fixed effect (time-invariant) term for each municipality, and u_{it} was the error term.

4. Results

4.1. Descriptive Analysis

In this section (from eFigures 10 – 18), we present figures that describe the temporal dynamics of the CCT programs, alongside the health outcomes (Under 5 mortality rates by age group and overall) from the year 2000 to 2019. During this period, the overall rate of U5 mortality reduced expressively, except for after the 2015 crisis, especially for the under-5 subgroups.

Regarding social welfare programs, eFigures 4-5, showed that the coverages of the CCT programs increased steadily from 2004 to 2018, although at a diminishing rate due to the long-term fiscal policy measure adopted since the 2013 economic crisis. In the year 2019, the reduction of the coverages of these programs was clear. eFigure 6 shows the average rate of mortality and hospitalization rates over the CCT coverage levels (in increasing order). In most cases, an inverse or negative correlation is perceptible between coverage levels and health outcomes.

We emphasize the difference between CCT crude coverage and CCT target coverage, in which the crude coverage is in relation to the total population of the municipality, while the target is in relation to the eligible poor population of the municipality. We know that the eligibility of each of the CCT programs depends on other factors besides the poverty criterion, however poverty is one of the main criteria. Thus, the term "target" is relative, being more focused on the population possibly benefited by these CCT programs compared to the total population of the municipality.

© 2023 Cavalcanti DM et al. *JAMA Network Open.*

eFigure 10. CCT crude coverage boxplot for selected municipalities in Brazil, Ecuador and Mexico, BEM, from the period 2000-19.

EACH COUNTRY – CCT CRUDE COVERAGE ALL COUNTRIES (BEM) – CCT CRUDE COVERAGE

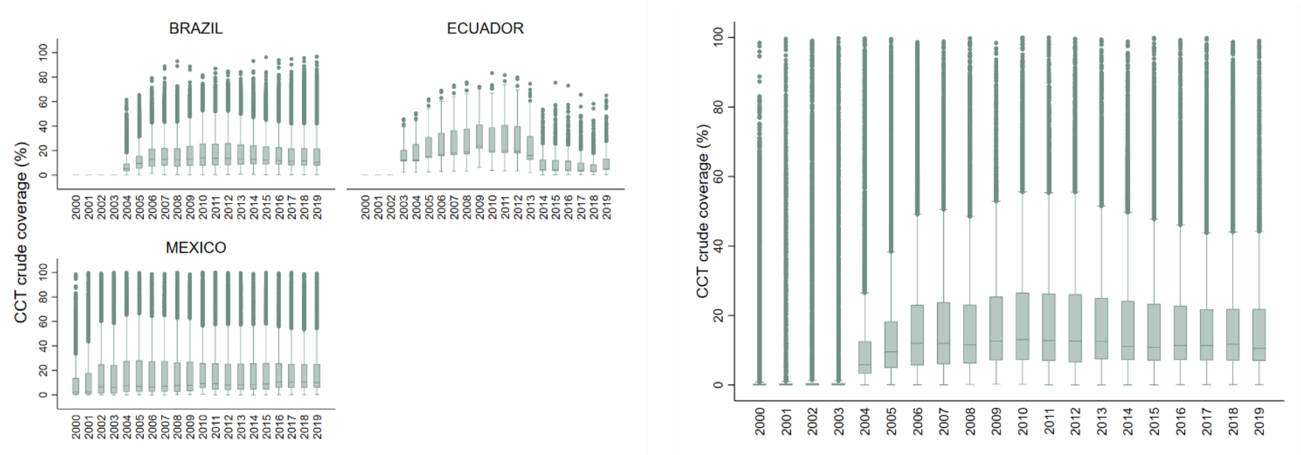

Source: Author's plot for 4,884 selected municipalities in Brazil, Ecuador, and Mexico, over 20 years (from 2000 to 2019).
Note: This variable refers to the conditional cash transference (CCT) programs coverage in relation to the total population of the municipality. We selected municipalities with adequate quality of civil registration and vital statistics (CRVS). In Brazil, Ecuador and Mexico, the number of beneficiaries families from CCT programs at municipal level are available for the years 2000-19 (no need extrapolations).

eFigure 11. CCT target coverage boxplot for selected municipalities in Brazil, Ecuador and Mexico, BEM, from the period 2000-19.

EACH COUNTRY – CCT CRUDE COVERAGE ALL COUNTRIES (BEM) – CCT CRUDE COVERAGE

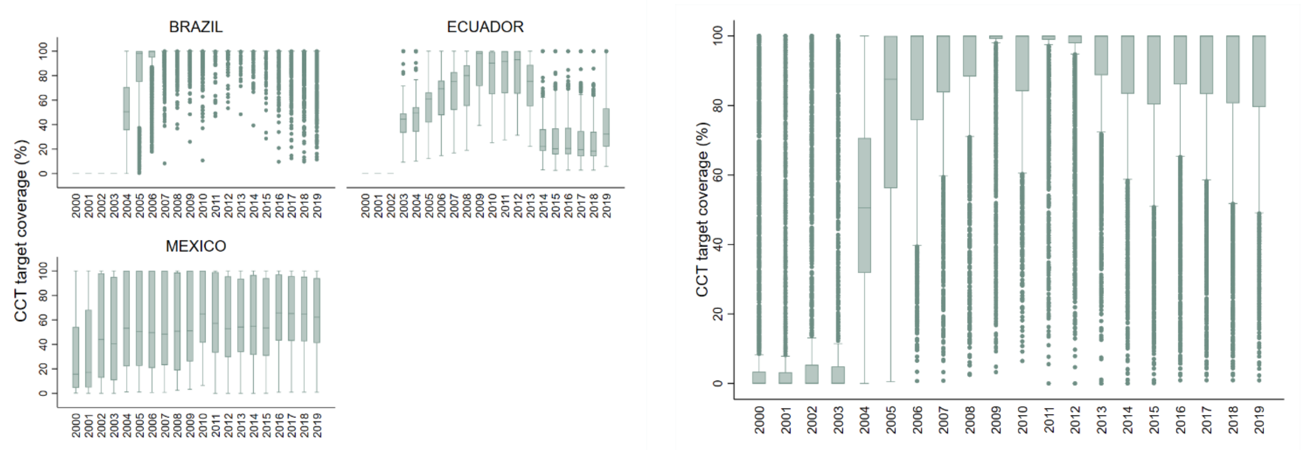

Source: Author's plot for 4,884 selected municipalities in Brazil, Ecuador, and Mexico, over 20 years (from 2000 to 2019).
Note: This variable refers to the conditional cash transference (CCT) programs coverage in relation to the poor population. We selected municipalities with adequate quality of civil registration and vital statistics (CRVS). In Brazil, Ecuador and Mexico, the number of beneficiaries families from CCT programs at municipal level are available for the years 2000-19 (no need extrapolations).

eFigure 12. CCT target and crude coverage for selected municipalities in Brazil, Ecuador and Mexico, BEM, from the period 2000-19.

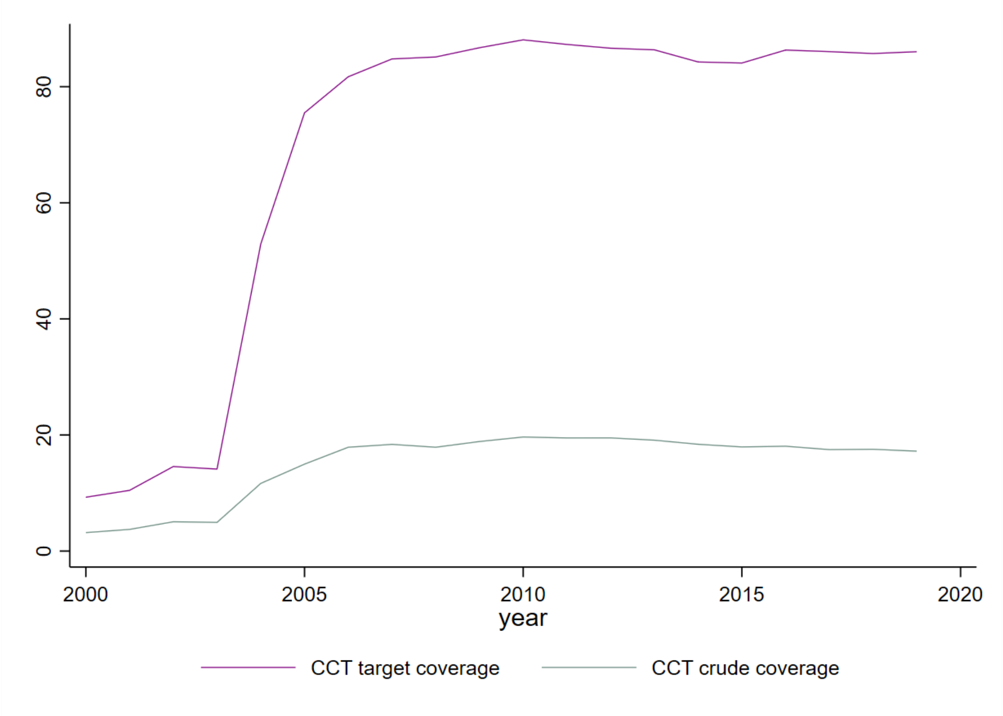

Source: Author's plot for 4,884 selected municipalities in Brazil, Ecuador, and Mexico, over 20 years (from 2000 to 2019).

eFigure 13. Child (under 5 years) mortality rate boxplot for selected municipalities in Brazil, Ecuador and Mexico, BEM, from the period 2000-19.

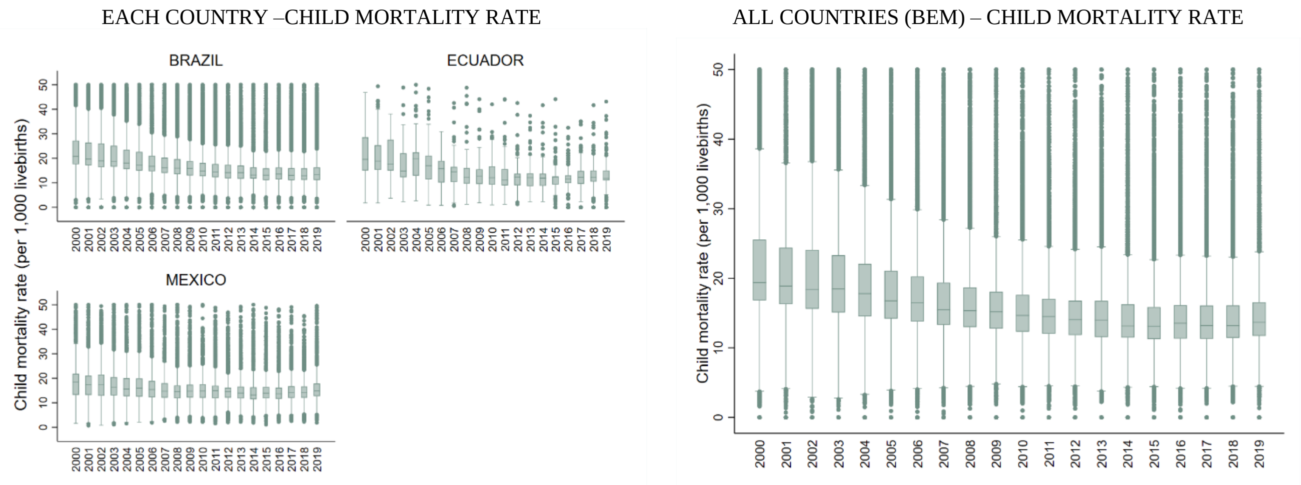

Source: Author's plot for 4,884 selected municipalities in Brazil, Ecuador, and Mexico, over 20 years (from 2000 to 2019).
Note: This variable refers to the child mortality (under-five years) per 1,000 livebirths. We selected municipalities with adequate quality of civil registration and vital statistics (CRVS).

© 2023 Cavalcanti DM et al. *JAMA Network Open.*

eFigure 14. Toddler (1 to 4 years) mortality boxplot for selected municipalities in Brazil, Ecuador and Mexico, BEM, from the period 2000-19.

EACH COUNTRY – TODDLER MORTALITY RATE

ALL COUNTRIES (BEM) – TODDLER MORTALITY RATE

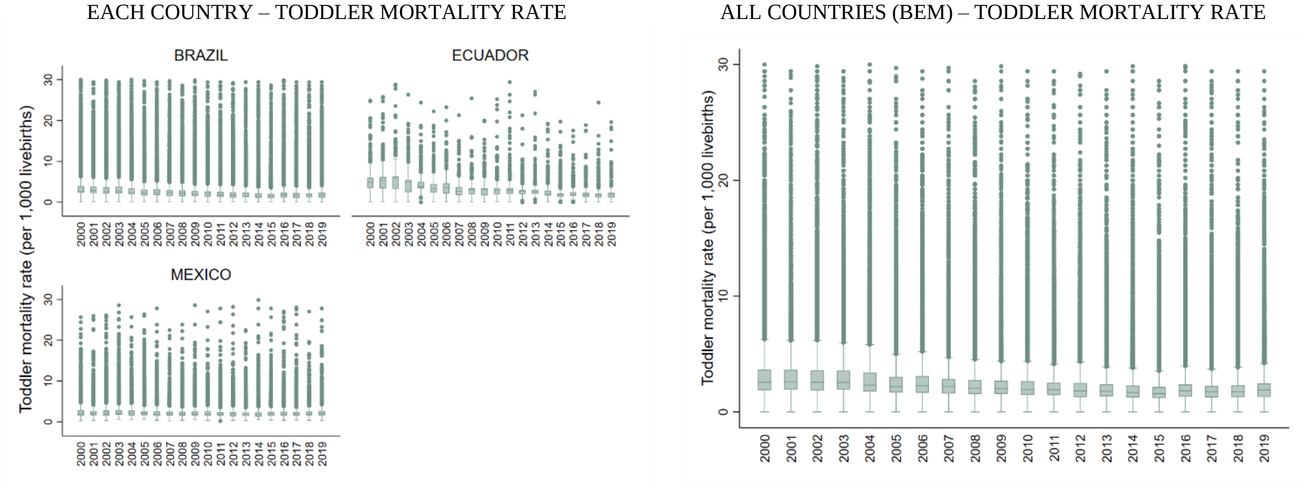

Source: Author's plot for 4,884 selected municipalities in Brazil, Ecuador, and Mexico, over 20 years (from 2000 to 2019).
Note: This variable refers to the toddler mortality (1 to 4 years) per 1,000 livebirths. We selected municipalities with adequate quality of civil registration and vital statistics (CRVS).

eFigure 15. Infant (under 1 year) mortality boxplot for selected municipalities in Brazil, Ecuador and Mexico, BEM, from the period 2000-19.

EACH COUNTRY –INFANT MORTALITY RATE

ALL COUNTRIES (BEM) – INFANT MORTALITY RATE

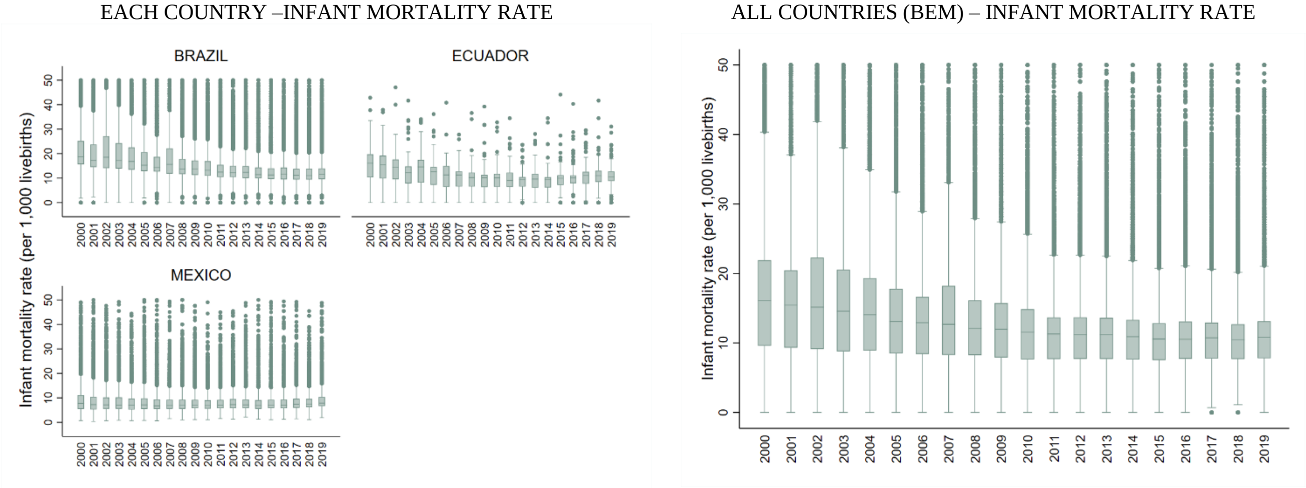

Source: Author's plot for 4,884 selected municipalities in Brazil, Ecuador, and Mexico, over 20 years (from 2000 to 2019).
Note: This variable refers to the infant mortality (under-one year) per 1,000 livebirths. We selected municipalities with adequate quality of civil registration and vital statistics (CRVS).

© 2023 Cavalcanti DM et al. *JAMA Network Open.*

eFigure 16. Post-neonatal (28 days to 1 year) mortality boxplot for selected municipalities in Brazil, Ecuador and Mexico, BEM, from the period 2000-19.

EACH COUNTRY –POST-NEONATAL MORTALITY RATE ALL COUNTRIES (BEM) – POST-NEONATAL MORTALITY RATE

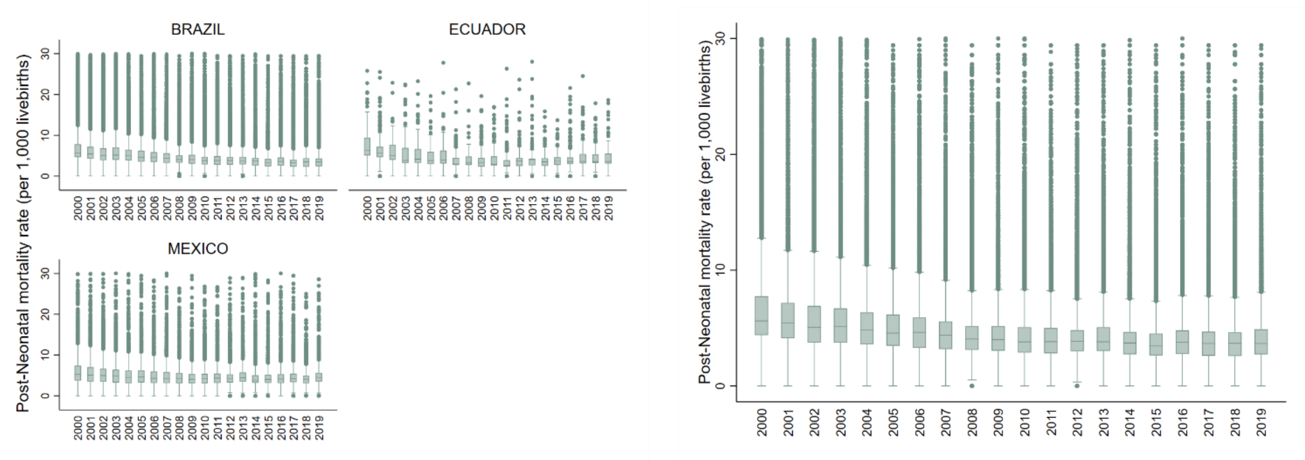

Source: Author's plot for 4,884 selected municipalities in Brazil, Ecuador, and Mexico, over 20 years (from 2000 to 2019).
Note: This variable refers to the post-neonatal mortality (28 days to 1 year) per 1,000 livebirths. We selected municipalities with adequate quality of civil registration and vital statistics (CRVS).

eFigure 17. Neonatal (0 to 28 days) mortality boxplot for selected municipalities in Brazil, Ecuador and Mexico, BEM, from the period 2000-19.

EACH COUNTRY – NEONATAL MORTALITY RATE ALL COUNTRIES (BEM) – NEONATAL MORTALITY RATE

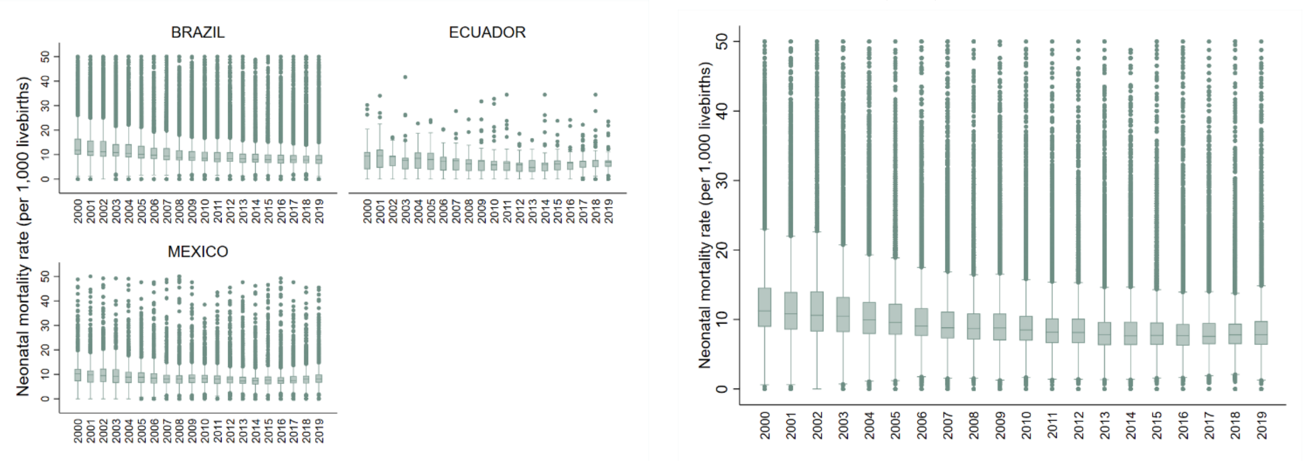

Source: Author's plot for 4,884 selected municipalities in Brazil, Ecuador, and Mexico, over 20 years (from 2000 to 2019).
Note: This variable refers to the neonatal mortality (0 to 28 days) per 1,000 livebirths. We selected municipalities with adequate quality of civil registration and vital statistics (CRVS).

© 2023 Cavalcanti DM et al. *JAMA Network Open.*

eFigure 18. All under-five mortality for selected municipalities in Brazil, Ecuador and Mexico, BEM, from the period 2000-19.

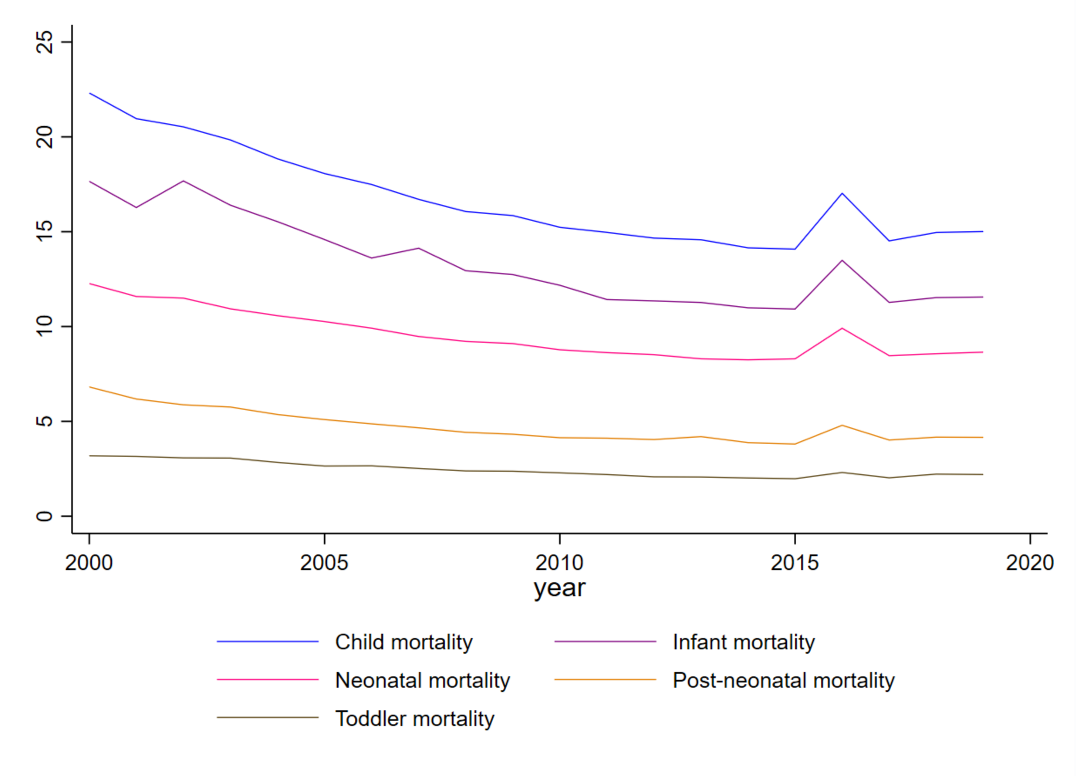

Source: Author's plot for 4,884 selected municipalities in Brazil, Ecuador, and Mexico, over 20 years (from 2000 to 2019).

4.2. Retrospective Analysis

4.3. Triangulation – Difference-in-difference with propensity score matching

We analysed the effect of CCT on U5MR by difference-in-difference (DID) with propensity score matching (PSM) as a triangulation approach,[18] using the municipalities with no or low CCT coverage since 2004 (n=1,175), versus medium and high coverage municipalities since 2004 (2,216), totaling 3,391 municipalities analyzed in the years 2004 and 2019.

We chose 2004 as the initial year of this analysis because this is the first year of the entire historical series worked on in which the 3 CCT programs started, being the first year of implementation of the *Bolsa Família* Program (Brazil).

The separation between low and high CCT coverage municipalities is the same used in the main regressions aggregating the highest categories, that is: low (0–29.9%), intermediate to high and consolidated (30–100%). Thus, municipalities with coverage less than 30% receive a value equal to 0 (control) and municipalities with coverage above 30% receive a value equal to 1 (treated). So that treatment status does not change between treated municipalities, we add a treatment duration effect, i.e., we filter municipalities that have no or low CCT program target coverage during the first 4 years (control) and municipalities with medium or high coverage of the CCT program in at least 4 years (treated). We tested different duration periods, such as 8 years or more, as well as testing different cutoffs to separate treated municipalities from control municipalities. We also tested different ways of estimating the DID, with the "diff" command in STATA and the step-by-step procedure described in the World Bank handbook, which allows estimating DID with a negative binomial panel of fixed effects and with coefficients in rate ratio (RR). We prefer this second one because it allows a more direct comparison with the results reported in the manuscript. The table below further separates these analysis strategies.

eTable 3. Different strategies for difference in difference models

Analysis strategies	Control (dummy=0)	Treated (dummy=1)	Time	Table
1	Low CCT coverage (<30%) at least 4 years	Intermediate to high CCT coverage (>=30%) at least 4 years	2004 and 2019	Table 4 ("diff" command)
2	Low CCT coverage (<30%) at least 4 years	Intermediate to high CCT coverage (>=30%) at least 4 years	2004 and 2019	Table 5 and 7
3	Low CCT coverage (<30%) at least 4 years	Intermediate to high CCT coverage (>=30%) at least 4 years	2004 to 2019	Table 6
4	Low CCT coverage (<30%) at least 8 years	Intermediate to high CCT coverage (>=30%) at least 8 years	2004 and 2019	Table 7
5	No CCT coverage (0%)	Some CCT coverage (>0%)	2004 and 2019	Table 7

Web-Tables 4 to 7 show the result of DID with PSM, coverages of CCT programs were associated with a statistically significant reduction in child mortality rates, with rate ratios(RR) of 0.990(95%CI:0.987-0.992). Even after approaching the municipalities by characteristics observed by the kernel matching method, the first difference in 2004 shows that municipalities with some coverage of the CCT program had a higher infant mortality rate compared to municipalities without coverage. The second difference, in 2019, shows that both municipalities with 0% CCT coverage and those with some level of CCT coverage reduced their child mortality. However, municipalities with coverage of CCT programs had an even greater reduction, so that the difference in difference was statistically significant. Thus, the CCT programs contributed to the reduction of infant mortality, being compatible with the results already found and described in the main manuscript, so that these results by DID with PSM reinforce the results found by the fixed effect panel with negative binomial, being a form of triangulation of results.

eTable 4. **Difference-in-difference with Propensity Score Matching for the association between child mortality rates and intermediate to high Conditional Cash Transference (CCT) coverage, in 2004 and 2019.**

	**Child**<br>Under 5 years	**Toddler**<br>1 to 4 years	**Infant**<br>Under 1 year	**Post Neonatal**<br>28 days to 1 year	**Neonatal**<br>0 to 28 days
Before (2004)					
Control	19.175	2.841	16.535	5.219	11.193
Treated	21.198	3.061	18.281	5.786	12.290
1st Difference (T-C)	2.023***	0.220	1.746***	0.567*	1.097***
	(0.708)	(0.211)	(0.004)	(0.098)	(0.390)
After (2019)					
Control	15.473	2.483	12.860	4.178	8.827
Treated	14.211	2.095	12.103	3.652	8.461
2nd Difference (T-C)	-1.262*	-0.388*	-0.757	-0.527	-0.366
	(0.717)	(0.214)	(0.221)	(0.128)	(0.354)
**Diff-in-Diff**	**-3.284***<br>(1.008)**	**-0.608**<br>(0.300)**	**-2.503***<br>(0.004)**	**-1.094**<br>(0.025)**	**-1.464***<br>(0.556)**

Source: Author's data analysis for 6,558 observations – 3,391 municipalities in Brazil, Ecuador and Mexico, with 1,175 municipalities like control (dummy = 0) and 2,216 municipalities like treated (dummy = 1) in in the years 2004 and 2019.
Note: Data are in mortality rate per 1,000 livebirths, with standard errors in parentheses. The symbols '***' , '**' and '*' denote significance at 1%, 5%, and 10% respectively. We use the STATA "diff" command, with kernel matching (PSM) to approximate the compared municipalities according to their observable characteristics.

eTable 5. Rate Ratios from the difference-in-difference fixed effect negative binomial models by under 5 age group for the association between mortality rates and intermediate to high Conditional Cash Transference (CCT) coverage, in 2004 and 2019.

	**Neonatal**<br>0 to 28 days	**Post-Neonatal**<br>28 days to 1 year	**Infamt**<br>Under 1 year	**Toddler**<br>1 to 4 years	**Child**<br>Under 5 years
**CCT target coverage (dummy)**					
CCT_4_y	545916609.5***	132044271.8***	85320745.7***	5.71332e+09***	5.31797e+09***
	[872755-3.5e+11]	[17218.7-1.01e+12]	[158456.2-4.6e+10]	[24969-1.3e+15]	[33572055-8.4e+11]
Intermediate to high (>=30%) for at least 4 years	0.991***<br>[0.987-0.994]	0.992***<br>[0.987-0.996]	0.991***<br>[0.988-0.994]	0.989***<br>[0.983-0.994]	0.990***<br>[0.987-0.992]
**Control Variables**				1.013***	
Poverty rate (%)	1.011***	1.012***	1.014***	[1.007-1.018]	1.009***
	[1.008-1.015]	[1.008-1.017]	[1.011-1.017]	1.008	[1.007-1.012]
Proportion of individuals older than 15	0.987***	1.001	1.003	[0.996-1.019]	0.994*
years who are illiterate (%)	[0.980-0.994]	[0.992-1.011]	[0.997-1.009]	1.001	[0.989-1.000]
Gini Index	1.003	1.009***	0.993***	[0.995-1.008]	1.004***
	[0.999-1.007]	[1.004-1.014]	[0.990-0.996]	1.005**	[1.001-1.007]

© 2023 Cavalcanti DM et al. *JAMA Network Open.*

Piped water	1.004***	1.004***	1.003***	[1.001-1.009]	1.003***
	[1.002-1.006]	[1.001-1.007]	[1.001-1.006]	0.996*	[1.001-1.005]
Proportion of individuals living in	0.993***	0.994***	0.997***	[0.992-1.000]	0.993***
households with inadequate sanitation	[0.991-0.996]	[0.991-0.997]	[0.995-0.999]	0.99	[0.992-0.995]
Hospital bed rate per 1,000 population	1.012	1.000	1.012	[0.957-1.024]	1.006
(%)	[0.995-1.030]	[0.978-1.023]	[0.996-1.028]	0.954**	[0.992-1.019]
Rate of physicians per 1,000 population	0.935***	0.914***	0.869***	[0.917-0.992]	0.938***
(%)	[0.915-0.956]	[0.886-0.942]	[0.850-0.889]	0.0511**	[0.922-0.954]
**Number of observations**	5,916	5,106	6,188	4,012	6,266
**Number of municipalities**	2,958	2,553	3,094	2,006	3,133

Source: Author's data analysis for 6,188 observations – 3,094 municipalities in Brazil, Ecuador and Mexico, in 2004 and 2019 period.
Note: Data are in Rate Ratio (RR) coefficients (95% CI) unless otherwise specified. The confidence intervals are in brackets. The symbols '***' , '**' and '*' denote significance at 1%, 5%, and 10% respectively.

eTable 6. Rate Ratios from the difference-in-difference fixed effect negative binomial models by under 5 age group for the association between mortality rates and intermediate to high Conditional Cash Transference (CCT) coverage, in all periods between 2000-19.

	**Neonatal** 0 to 28 days	**Post-Neonatal** 28 days to 1 year	**Infant** Under 1 year	**Toddler** 1 to 4 years	**Child** Under 5 years
**CCT target coverage (dummy)**					
CCT_4_y	2.22538e+11***	47286207.8***	737632377.6***	1042.792***	8.33179e+10***
	[2.1e+10 – 2.5e+12]	[1773790 – 1.3e+09]	[79187081 – 6.9e+09]	[13.58 – 80053.96]	[1.4e+10 – 5.1e+11]
Intermediate to high (>=30%) for at	0.987***	0.991***	0.990***	0.986***	0.988***
least 4 years	[0.986 – 0.988]	[0.990 – 0.993]	[0.989 – 0.991]	[0.984 – 988]	[0.987 – 0.989]
**Control variables**					
Poverty rate (%)	1.006***	1.012***	1.013***	1.010***	1.008***
	[1.006 – 1.007]	[1.011 – 1.013]	[1.012 – 1.013]	[1.009 – 1.012]	[1.007 – 1.008]
Proportion of individuals older than 15	0.998	1.017***	1.012***	1.009***	1.005***
years who are illiterate (%)	[0.996 – 1.000]	[1.014 – 1.020]	[1.010 – 1.014]	[1.005 – 1.013]	[1.003 – 1.007]
Gini Index	1.005***	1.006***	0.993***	1.006***	1.005***
	[1.004 – 1.006]	[1.004 – 1.007]	[0.992 – 0.994]	[1.004 – 1.008]	[1.004 – 1.006]
Piped water	1.004***	1.002***	1.001**	1.003***	1.003***
	[1.003 – 1.005]	[1.001 – 1.003]	[1.000 – 1.001]	[1.002 – 1.004]	[1.002 – 1.003]
Proportion of individuals living in	0.995***	0.996***	0.999***	0.999	0.996***
households with inadequate sanitation	[0.994 – 0.995]	[0.995 – 0.996]	[0.998 – 0.999]	[0.998 – 1.000]	[0.995 – 0.996]
Hospital bed rate per 1,000 population	1.009***	1.009**	1.005**	1.005	1.008***
(%)	[1.004 – 1.015]	[1.000 – 1.018]	[1.000 – 1.011]	[0.993 – 1.016]	[1.004 – 1.013]
Rate of physicians per 1,000 population	0.944***	0.936***	0.855***	0.910***	0.941***
(%)	[0.937 – 0.952]	[0.925 – 0.947]	[0.847 – 0.862]	[0.897 – 0.925]	[0.935 – 0.947]
**Year control**	Yes	Yes	Yes	Yes	Yes
**Number of observations**	69,587	68,850	69,594	69,599	69,599
**Number of municipalities**	3,688	3,637	3,390	3,691	3,691

Source: Author's data analysis for 69,850 observations – 3,446 municipalities in Brazil, Ecuador and Mexico, over 20 years (from 2000 to 2019).
Note: Data are in Rate Ratio (RR) coefficients (95% CI) unless otherwise specified. The confidence intervals are in brackets. Time shocks are controls for specific years of economic crisis (2008, 2013, and 2015) and for specific years of changes in CCT programs (2003, 2004, and 2014). The symbols '***' , '**' and '*' denote significance at 1%, 5%, and 10% respectively.

© 2023 Cavalcanti DM et al. *JAMA Network Open.*

eTable 7. Rate Ratios from the difference-in-difference fixed effect negative binomial models for the association between U5MR and different specification for Conditional Cash Transference (CCT) coverage, in 2004 and 2019.

	Low CCT coverage (<30%) *versus* Intermediate or more (>=30%), by duration		No coverage (0%) *versus* some coverage (>0%)
	4 years	8 years	
**CCT target coverage (dummy)**			
	5.31797e+09***	-	-
	[33572055-8.4e+11]	-	-
	-	5.62864e+12***	-
	-	[2.060e+08-1.538e+17]	-
	-	-	144373.2***
	-	-	[2816.3-7401182.3]
Intermediate to high (>=30%) versus low (<30%) for at least 4 years	0.990***	-	-
	[0.987-0.992]	-	-
Intermediate to high (>=30%) versus low (<30%) for at least 8 years	-	0.986***	-
	-	[0.982-0.991]	-
Some coverage (>0%) versus no coverage (0%)	-	-	0.994***
	-	-	[0.992-0.996]
**Control variables**			
Poverty rate (%)	1.009***	1.010***	1.011***
	[1.007-1.012]	[1.004-1.016]	[1.009-1.013]
Proportion of individuals older than 15 years who are illiterate (%)	0.994*	0.994	0.997
	[0.989-1.000]	[0.978-1.010]	[0.992-1.002]
Gini Index	1.004***	0.998	1.002
	[1.001-1.007]	[0.993-1.003]	[0.999-1.004]
Piped water	1.003***	1.004**	1.004***
	[1.001-1.005]	[1.000-1.009]	[1.003-1.006]
Proportion of individuals living in households with inadequate sanitation	0.993***	0.991***	0.993***
	[0.992-0.995]	[0.987-0.996]	[0.992-0.995]
Hospital bed rate per 1,000 population (%)	1.006	1.035**	1.013**
	[0.992-1.019]	[1.003-1.068]	[1.001-1.025]
Rate of physicians per 1,000 population (%)	0.938***	1.008	0.943***
	[0.922-0.954]	[0.975-1.041]	[0.926-0.960]
**Number of observations**	6,266	1,306	8,888
**Number of municipalities**	3,808	653	4,444

Source: Author's data analysis for 6,188 observations – 3,094 municipalities in Brazil, Ecuador and Mexico, in 2004 and 2019 period.

Note: Data are in Rate Ratio (RR) coefficients (95% CI) unless otherwise specified. The confidence intervals are in brackets. The symbols '***' , '**' and '*' denote significance at 1%, 5%, and 10% respectively.

4.4. Fit and sensitivity tests

Concerning our study about the effect of social protection on child mortality, we developed several sensitivity tests in order to guarantee the robustness of our results.

As a first test, for choosing between a random and a fixed effect, we performed the Hausmann test for the child mortality data. By following different goodness of fit criteria including AIC, BIC and the Log-likelihood, we conclude that the fixed effects model were more adequate to analyze the effects of cash transfer and social pension policies on this kind of mortality. eTable 8 shows this test.

Second, we change the model specification, measure the pure regression (without covariates), and omit some variables (eTable 9).

Third, to evaluate the influence of the categorization, we estimated the models by changing the thresholds by quartile approach (eTable 10) and using continuous variables (eTable 11). We also tested the behavior of models when switching from CCT target coverage to CCT municipal (crude) coverage, applying both to continuous variables (eTable 12) and to categorical variables (eTable 13).

Forth, to evaluate the external validity of our estimates, we fit the models using all BEM 8,103 municipalities (i.e., including municipalities with vital information of lower quality, in eTable 14).

Fifth, to test the relevance of the time dummies, we tested different sets of time variables (eTable 15).

Sixth, to evaluate the stability of the results with alternative regression models, we fitter Poisson regression models and compared their Akaike Information Criterion (AIC) and Bayesian Information Criterion (BIC) with negative binomial models (see eTable 16). This results showed that the Negative Binomial method yields better estimates. This is reasonable since the negative binomial regression takes the data overdispersion into account, compared to the Poisson which does not.

Seventh, we performed a series of heterogeneity of effects analyses, dividing the model by poverty quartiles (see eTable 17), and estimating the model separately by countries (see eTable 18).

Eight, we estimated a Falsification/ placebo tests, all aiming to verify unexpected effects on the exposure variable or the dependent variable, using as outcome child mortality due to external causes (ICD10 codes W01-Y99), which CCT are not expected to influence this group of causes and do not include actions for its prevention (see eTable 19).

In other words, given that our results withstood the vast number of sensitivity tests performed, we conclude that the results and conclusions drawn in this study are robust and stable.

eTable 8. Hausman test between fixed effect and random effect negative binomial models

	**Mortality**		**Hospitalization**	
	**Fixed Effect**	**Random effect**	**Fixed Effect**	**Random effect**
Number of observations	95,130	95,132	94,309	94,317
Number of municipalities	4,882	4,884	4,882	4,885
Log likelihood	-199402.41	-222357.1	-3666598.5	-3717208.3
Akaike information criterion (AIC)	398836.8	444750.2	7333227	7434451
Bayesian information criterion (BIC)	398988.2	444920.5	7333369	7434611
**Hausman test**	χ^2**=1029.47;** p**-value= 0.000**		χ^2**=1660.12;** p**-value= 0.000**	

Source: Author's data analysis for 95,132 observations - 4,884 municipalities in Brazil, Ecuador and Mexico, over 20 years (from 2000 to 2019).

eTable 9. Rate Ratios from the fixed effect negative binomial models for the association between under five mortality rates and Conditional Cash Transference (CCT) target coverage program, for Omitted variable bias test, from the period 2000-19, in Brazil, Ecuador and Mexico

	**UNDER FIVE MORTALITY RATE**				
	**Pure regression**	**Whitou time control**	**Without selected variables**	**Without selected variables**	**All variables**
**CCT target population coverage**					
Low (0 – 29.9%)	1	1	1	1	1
	[1.000-1.000]	[1.000-1.000]	[1.000-1.000]	[1.000-1.000]	[1.000-1.000]
Intermediate (30 – 69.9%)	0.871***	0.884***	0.883***	0.878***	0.880***
	[0.865-0.878]	[0.878-0.891]	[0.877-0.890]	[0.871-0.884]	[0.873-0.887]
High (70 – 99.9%)	0.815***	0.839***	0.832***	0.832***	0.840***
	[0.808-0.822]	[0.831-0.846]	[0.825-0.839]	[0.825-0.839]	[0.832-0.847]
Consolidated (100%)	0.695***	0.748***	0.722***	0.738***	0.757***
	[0.691-0.699]	[0.744-0.753]	[0.717-0.726]	[0.733-0.743]	[0.752-0.762]
**Control variables**					
Proportion of individuals older than 15 years who are illiterate (%)		1.052***	1.092***	1.064***	1.047***
		[1.041-1.063]	[1.081-1.103]	[1.053-1.075]	[1.036-1.058]
Gini Index		1.084***	1.080***		1.072***
		[1.076-1.093]	[1.072-1.089]		[1.064-1.081]
Piped water		0.973***	0.964***	0.963***	0.979***
		[0.964-0.983]	[0.955-0.974]	[0.954-0.973]	[0.969-0.988]
Rate of physicians per 1,000 population (%)		0.985***	0.982***		0.989**

© 2023 Cavalcanti DM et al. *JAMA Network Open.*

		[0.976-0.994]	[0.973-0.991]		[0.980-0.998]
Poverty rate (%)		1.074***		1.075***	1.072***
		[1.064-1.084]		[1.065-1.085]	[1.063-1.082]
Proportion of individuals living in households with inadequate sanitation (%)		0.902***		0.903***	0.907***
		[0.893-0.910]		[0.895-0.912]	[0.898-0.915]
Hospital bed rate per 1,000 population (%)		1.007		1.015**	1.006
		[0.995-1.019]		[1.003-1.027]	[0.995-1.018]
**Time trend control**					
2003			1.033***		1.032***
			[1.024-1.042]		[1.023-1.041]
2004					1.050***
					[1.040-1.059]
2008					1.022***
					[1.013-1.031]
2013					0.967***
					[0.957-0.976]
2015					0.916***
					[0.907-0.925]
**Number of observations**	**95,281**	**95,130**	**95,130**	**95,130**	**95,130**
**Number of municipalities**	**4,891**	**4,882**	**4,882**	**4,882**	**4,882**

Source: Author's data analysis for 95,281 observations - 4,881 municipalities in Brazil, Ecuador and Mexico, over 20 years (from 2000 to 2019).
Note: Data are in Rate Ratio (RR) coefficients (95% CI) unless otherwise specified. The confidence intervals are in parentheses. Time shocks are controls for specific years of economic crisis (2008, 2013, and 2015) and for specific years of changes in CCT programs (2003, 2004, and 2014). The symbols '***' , '**' and '*' denote significance at 1%, 5%, and 10% respectively.

eTable 10. Rate Ratios from the fixed effect negative binomial models for the association between under five mortality rates and Conditional Cash Transference (CCT) target coverage program categorization changing from fixed category to quartile, from the period 2000-19, in Brazil, Ecuador and Mexico

	MORTALITY				
	**Neonatal**	**Post Neonatal**	**Infant**	**Toddler**	**Child**
	0 to 28 days	28 days to 1 year	Under 1 year	1 to 4 years	Under 5 years
**CCT target population coverage (quartile)**					
Low	1	1	1	1	1
	[1.000-1.000]	[1.000-1.000]	[1.000-1.000]	[1.000-1.000]	[1.000-1.000]
Intermediate	0.850***	0.749***	0.788***	0.778***	0.810***
	[0.843-0.856]	[0.741-0.757]	[0.782-0.793]	[0.768-0.789]	[0.805-0.815]
High	0.887***	0.726***	0.806***	0.768***	0.813***
	[0.853-0.922]	[0.693-0.761]	[0.780-0.833]	[0.733-0.804]	[0.792-0.836]
Consolidated	0.743***	0.655***	0.696***	0.756***	0.727***
	[0.718-0.770]	[0.627-0.685]	[0.676-0.718]	[0.722-0.791]	[0.709-0.746]
**Control Variables**					
Poverty rate (%)	1.131***	1.035***	1.150***	1.127***	1.097***
	[1.118-1.145]	[1.019-1.052]	[1.138-1.162]	[1.105-1.151]	[1.087-1.108]
Proportion of individuals older than 15 years who are illiterate (%)	1.039***	1.063***	1.079***	1.067***	1.051***
	[1.025-1.053]	[1.044-1.083]	[1.065-1.092]	[1.043-1.092]	[1.040-1.063]
Gini Index	1.091***	1.113***	1.005	1.047***	1.094***
	[1.080-1.102]	[1.098-1.128]	[0.996-1.015]	[1.028-1.065]	[1.085-1.102]
Piped water	1.006	0.919***	0.955***	0.970***	0.972***
	[0.994-1.019]	[0.904-0.935]	[0.944-0.966]	[0.950-0.990]	[0.963-0.982]
Proportion of individuals living in households with inadequate sanitation (%)	0.909***	0.854***	0.940***	0.898***	0.886***
	[0.898-0.920]	[0.840-0.869]	[0.929-0.950]	[0.879-0.916]	[0.878-0.895]
Hospital bed rate per 1,000 population (%)	1.009	1.018*	0.962***	1.011	1.014**

© 2023 Cavalcanti DM et al. *JAMA Network Open.*

	[0.994-1.024]	[0.997-1.038]	[0.949-0.975]	[0.985-1.037]	[1.002-1.026]
Rate of physicians per 1,000 population (%)	1.008	0.975***	0.943***	0.957***	0.990**
	[0.997-1.021]	[0.959-0.990]	[0.933-0.954]	[0.939-0.977]	[0.981-1.000]
Year binaries	Yes	Yes	Yes	Yes	Yes
**Number of observations**	**95,312**	**94,523**	**95,352**	**87,155**	**95,201**
**Number of municipalities**	**4,879**	**4,835**	**4,881**	**4,662**	**4,882**

Source: Author's data analysis for 95,352 observations - 4,881 municipalities in Brazil, Ecuador and Mexico, over 20 years (from 2000 to 2019).
Note: Data are in Rate Ratio (RR) coefficients (95% CI) unless otherwise specified. The confidence intervals are in parentheses. Time shocks are controls for specific years of economic crisis (2008, 2013, and 2015) and for specific years of changes in CCT programs (2003, 2004, and 2014). The symbols '***' , '**' and '*' denote significance at 1%, 5%, and 10% respectively.

eTable 11. Rate Ratios from the fixed effect negative binomial models for the association between under five mortality rates and Conditional Cash Transference (CCT) target coverage using continuous variables; from the period 2000-19, in Brazil, Ecuador and Mexico.

	UNDER-FIVE MORTALITY RATE BY AGE GROUPS				
	**Neonatal**<br>0 to 28 days	**Post Neonatal**<br>28 days to 1 year	**Infant**<br>Under 1 year	**Toddler**<br>1 to 4 years	**Child**<br>Under 5 years
**CCT target coverage**	0.999***	0.999***	0.998***	0.999***	0.999***
	[0.999-0.999]	[0.998-0.999]	[0.998-0.999]	[0.999-1.000]	[0.999-0.999]
**Control variables**					
Poverty rate (%)	1.004***	1.008***	1.009***	1.013***	1.006***
	[1.004-1.005]	[1.008-1.009]	[1.008-1.010]	[1.012-1.014]	[1.006-1.007]
Proportion of individuals older than 15 years who are illiterate (%)	0.997***	1.021***	1.017***	1.005***	1.004***
	[0.995-0.999]	[1.018-1.023]	[1.015-1.018]	[1.001-1.008]	[1.003-1.006]
Gini Index	1.002***	1.001*	0.992***	0.998*	1.001***
	[1.001-1.003]	[1.000-1.003]	[0.991-0.993]	[0.997-1.000]	[1.001-1.002]
Piped water	1.004***	1.001***	1	1.003***	1.003***
	[1.003-1.004]	[1.001-1.002]	[1.000-1.001]	[1.002-1.004]	[1.002-1.003]
Proportion of individuals living in households with inadequate sanitation (%)	0.997***	0.998***	1.001***	1.001***	0.997***
	[0.997-0.998]	[0.997-0.998]	[1.000-1.001]	[1.001-1.002]	[0.997-0.998]
Hospital bed rate per 1,000 population (%)	1.007***	1.004	1.006***	0.997	1.006***
	[1.002-1.011]	[0.998-1.011]	[1.002-1.011]	[0.988-1.006]	[1.002-1.009]
Rate of physicians per 1,000 population (%)	0.983***	0.984***	0.866***	0.928***	0.976***
	[0.975-0.991]	[0.973-0.995]	[0.859-0.873]	[0.914-0.942]	[0.970-0.983]
Time trend	0.988***	0.993***	0.997***	0.985***	0.989***
	[0.987-0.989]	[0.992-0.994]	[0.996-0.998]	[0.984-0.987]	[0.988-0.990]
**Number of observations**	**95,312**	**94,523**	**95,352**	**87,155**	**95,201**
**Number of municipalities**	**4,879**	**4,835**	**4,881**	**4,662**	**4,882**

Source: Author's data analysis for 95,352 observations - 4,881 municipalities in Brazil, Ecuador and Mexico, over 20 years (from 2000 to 2019).
Note: Data are in Rate Ratio (RR) coefficients (95% CI) unless otherwise specified. The confidence intervals are in parentheses. The symbols '***' , '**' and '*' denote significance at 1%, 5%, and 10% respectively.

eTable 12. Rate Ratios from the fixed effect negative binomial models for the association between under five mortality rates and Conditional Cash Transference (CCT) crude (municipal) coverage using continuous variables; from the period 2000-19, in Brazil, Ecuador and Mexico.

	UNDER-FIVE MORTALITY RATE BY AGE GROUPS				
	**Neonatal**<br>0 to 28 days	**Post Neonatal**<br>28 days to 1 year	**Infant**<br>Under 1 year	**Toddler**<br>1 to 4 years	**Child**<br>Under 5 years

© 2023 Cavalcanti DM et al. *JAMA Network Open.*

**CCT cude (municipal) coverage**	0.920***	0.570***	0.800***	0.828***	0.761***
	[0.886-0.956]	[0.544-0.597]	[0.775-0.826]	[0.781-0.877]	[0.740-0.783]
**Control variables**					
Poverty rate (%)	1.007***	1.008***	1.012***	1.013***	1.007***
	[1.006-1.007]	[1.007-1.009]	[1.011-1.012]	[1.012-1.014]	[1.007-1.008]
Proportion of individuals older than 15 years who are illiterate (%)	0.993***	1.012***	1.011***	1.002	0.999
	[0.991-0.995]	[1.010-1.015]	[1.009-1.012]	[0.998-1.005]	[0.998-1.001]
Gini Index	1.002***	1.002***	0.992***	0.999	1.002***
	[1.001-1.003]	[1.001-1.003]	[0.991-0.993]	[0.997-1.000]	[1.001-1.002]
Piped water	1.004***	1.002***	1.001***	1.003***	1.003***
	[1.003-1.005]	[1.001-1.002]	[1.000-1.001]	[1.002-1.004]	[1.003-1.003]
Proportion of individuals living in households with inadequate sanitation (%)	0.997***	0.999***	1.001***	1.002***	0.998***
	[0.997-0.998]	[0.998-0.999]	[1.000-1.001]	[1.001-1.002]	[0.998-0.998]
Hospital bed rate per 1,000 population (%)	1.007***	1.007*	1.007***	0.998	1.007***
	[1.003-1.012]	[1.000-1.013]	[1.003-1.012]	[0.989-1.008]	[1.003-1.010]
Rate of physicians per 1,000 population (%)	0.983***	0.972***	0.864***	0.926***	0.972***
	[0.975-0.991]	[0.961-0.983]	[0.857-0.871]	[0.912-0.940]	[0.966-0.979]
Time trend	0.984***	0.989***	0.992***	0.984***	0.986***
	[0.984-0.985]	[0.988-0.990]	[0.991-0.993]	[0.982-0.985]	[0.985-0.986]
**Number of observations**	**95,312**	**94,523**	**95,352**	**87,155**	**95,201**
**Number of municipalities**	**4,879**	**4,835**	**4,881**	**4,662**	**4,882**

Source: Author's data analysis for 95,352 observations - 4,881 municipalities in Brazil, Ecuador and Mexico, over 20 years (from 2000 to 2019).
Note: Data are in Rate Ratio (RR) coefficients (95% CI) unless otherwise specified. The confidence intervals are in parentheses. The symbols '***' , '**' and '*' denote significance at 1%, 5%, and 10% respectively.

eTable 13. Rate Ratios from the fixed effect negative binomial models for the association between under five mortality rates and Conditional Cash Transference (CCT) crude (municipal) coverage using categorical variables; from the period 2000-19, in Brazil, Ecuador and Mexico.

	UNDER-FIVE MORTALITY RATE BY AGE GROUPS				
	**Neonatal**	**Post Neonatal**	**Infant**	**Toddler**	**Child**
	0 to 28 days	28 days to 1 year	Under 1 year	1 to 4 years	Under 5 years
**CCT cude (municipal) coverage**					
Low (0 – 5.9%)	1	1	1	1	1
	[1.000-1.000]	[1.000-1.000]	[1.000-1.000]	[1.000-1.000]	[1.000-1.000]
Intermediate (6 – 14.7%)	0.870***	0.825***	0.836***	0.854***	0.855***
	[0.863-0.877]	[0.816-0.834]	[0.830-0.843]	[0.842-0.866]	[0.849-0.860]
High (14.8 – 33.3%)	0.839***	0.757***	0.792***	0.808***	0.811***
	[0.830-0.848]	[0.746-0.768]	[0.784-0.800]	[0.794-0.822]	[0.804-0.818]
Consolidated (33.4 - 100%)	0.818***	0.573***	0.724***	0.713***	0.720***
	[0.806-0.829]	[0.563-0.584]	[0.714-0.733]	[0.697-0.729]	[0.712-0.728]
**Control Variables**					
Poverty rate (%)	1.147***	1.072***	1.190***	1.170***	1.125***
	[1.134-1.161]	[1.055-1.090]	[1.177-1.203]	[1.146-1.194]	[1.115-1.136]
Proportion of individuals older than 15 years who are illiterate (%)	1.041***	1.086***	1.091***	1.077***	1.061***
	[1.027-1.055]	[1.066-1.106]	[1.077-1.105]	[1.053-1.102]	[1.050-1.072]
Gini Index	1.097***	1.121***	1.020***	1.058***	1.101***
	[1.086-1.108]	[1.106-1.136]	[1.010-1.029]	[1.039-1.076]	[1.092-1.109]
Piped water	1	0.940***	0.950***	0.972***	0.975***
	[0.988-1.013]	[0.924-0.956]	[0.939-0.961]	[0.952-0.993]	[0.966-0.985]
Proportion of individuals living in households with inadequate sanitation (%)	0.911***	0.899***	0.953***	0.905***	0.904***
	[0.900-0.922]	[0.884-0.914]	[0.943-0.964]	[0.886-0.924]	[0.895-0.913]
Hospital bed rate per 1,000 population (%)	1.008	1.005	0.956***	1.009	1.008
	[0.993-1.024]	[0.985-1.025]	[0.943-0.969]	[0.983-1.035]	[0.996-1.020]
Rate of physicians per 1,000 population (%)	1.003	0.972***	0.936***	0.956***	0.985***
	[0.991-1.015]	[0.956-0.987]	[0.926-0.946]	[0.937-0.975]	[0.976-0.994]
**Time trend control - specific years**					
2003	1.010*	1.01	1.026***	1.066***	1.022***
	[0.998-1.021]	[0.996-1.025]	[1.015-1.037]	[1.046-1.086]	[1.014-1.031]
2004	1.054***	1.057***	1.084***	1.093***	1.061***
	[1.042-1.066]	[1.042-1.073]	[1.073-1.096]	[1.072-1.113]	[1.052-1.071]
2008	1.01	0.996	1.023***	1.026**	1.008*

© 2023 Cavalcanti DM et al. *JAMA Network Open.*

	[0.998-1.022]	[0.981-1.012]	[1.012-1.035]	[1.005-1.047]	[0.999-1.017]
2013	0.945***	1.006	0.954***	0.931***	0.960***
	[0.934-0.957]	[0.990-1.023]	[0.943-0.965]	[0.910-0.951]	[0.950-0.969]
2015	0.935***	0.894***	0.910***	0.847***	0.909***
	[0.923-0.946]	[0.879-0.910]	[0.899-0.921]	[0.827-0.866]	[0.900-0.918]
**Number of observations**	**95,312**	**94,523**	**95,352**	**87,155**	**95,201**
**Number of municipalities**	**4,879**	**4,835**	**4,881**	**4,662**	**4,882**

Source: Author's data analysis for 95,352 observations - 4,881 municipalities in Brazil, Ecuador and Mexico, over 20 years (from 2000 to 2019).
Note: Data are in Rate Ratio (RR) coefficients (95% CI) unless otherwise specified. The confidence intervals are in parentheses. Time shocks are controls for specific years of economic crisis (2008, 2013, and 2015) and for specific years of changes in CCT programs (2003, 2004, and 2014). The symbols '***' , '**' and '*' denote significance at 1%, 5%, and 10% respectively.

eTable 14. Rate Ratios from the fixed effect negative binomial models for the association between under five mortality rates and Conditional Cash Transference (CCT) target coverage using categorical variables, and without filter for adequate information (QVI); from the period 2000-19, in all municipalities in Brazil, Ecuador and Mexico.

	**UNDER-FIVE MORTALITY RATE BY AGE GROUPS**				
	**Neonatal**<br>0 to 28 days	**Post Neonatal**<br>28 days to 1 year	**Infant**<br>Under 1 year	**Toddler**<br>1 to 4 years	**Child**<br>Under 5 years
**CCT target population coverage**					
Low (0 – 29.9%)	1	1	1	1	1
	[1.000-1.000]	[1.000-1.000]	[1.000-1.000]	[1.000-1.000]	[1.000-1.000]
Intermediate (30 – 69.9%)	0.899***	0.856***	0.916***	0.887***	0.877***
	[0.892-0.907]	[0.846-0.865]	[0.909-0.923]	[0.875-0.900]	[0.871-0.882]
High (70 – 99.9%)	0.874***	0.794***	0.869***	0.838***	0.839***
	[0.865-0.882]	[0.784-0.804]	[0.862-0.877]	[0.824-0.852]	[0.832-0.845]
Consolidated (100%)	0.805***	0.702***	0.737***	0.757***	0.761***
	[0.799-0.811]	[0.696-0.709]	[0.733-0.742]	[0.747-0.767]	[0.757-0.766]
**Control Variables**					
Poverty rate (%)	1.119***	1.152***	1.165***	1.181***	1.085***
	[1.107-1.130]	[1.136-1.168]	[1.154-1.175]	[1.160-1.203]	[1.076-1.095]
Proportion of individuals older than 15 years who are illiterate (%)	1.063***	1.105***	1.096***	1.123***	1.083***
	[1.051-1.075]	[1.089-1.121]	[1.085-1.107]	[1.102-1.144]	[1.073-1.093]
Gini Index	1.076***	1.074***	1.003	1.031***	1.073***
	[1.067-1.085]	[1.062-1.087]	[0.995-1.010]	[1.016-1.047]	[1.066-1.081]
Piped water	1.001	0.941***	0.969***	1.014	0.986***
	[0.990-1.012]	[0.928-0.955]	[0.960-0.978]	[0.996-1.033]	[0.978-0.995]
Proportion of individuals living in households with inadequate sanitation (%)	0.958***	0.881***	0.988**	0.953***	0.911***
	[0.948-0.969]	[0.868-0.893]	[0.979-0.998]	[0.936-0.970]	[0.903-0.919]
Hospital bed rate per 1,000 population (%)	0.979***	0.989	0.934***	0.999	0.999
	[0.967-0.992]	[0.973-1.006]	[0.924-0.945]	[0.978-1.021]	[0.989-1.010]
Rate of physicians per 1,000 population (%)	0.997	0.947***	0.952***	0.958***	0.984***
	[0.988-1.006]	[0.936-0.959]	[0.944-0.960]	[0.943-0.973]	[0.976-0.991]
Year binaries	Yes	Yes	Yes	Yes	Yes
**Number of observations**	**154,118**	**154,342**	**156,143**	**143,943**	**150,260**
**Number of municipalities**	**8,103**	**8,011**	**8,114**	**7,730**	**8,058**

Source: Author's data analysis for 154,118 observations – 8,103 municipalities in Brazil, Ecuador and Mexico, over 20 years (from 2000 to 2019).
Note: Data are in Rate Ratio (RR) coefficients (95% CI) unless otherwise specified. The confidence intervals are in parentheses. Time shocks are controls for specific years of economic crisis (2008, 2013, and 2015) and for specific years of changes in CCT programs (2003, 2004, and 2014). The symbols '***' , '**' and '*' denote significance at 1%, 5%, and 10% respectively.

eTable 15. Rate Ratios from the fixed effect negative binomial models for the association between under five mortality rates and Conditional Cash Transference (CCT) target coverage using categorical variables, with different time shocks control; from the period 2000-19, in all municipalities in Brazil, Ecuador and Mexico.

	**UNDER-FIVE MORTALITY RATE BY DIFFERENT TIME SHOCK CONTROL**					
	**No time Control**	**Fist 5 years**<br>2000 to 2004	**Second 5 years**<br>2005 to 2009	**Third 5 years**<br>2010 to 2014	**Last 5 years**<br>2015 to 2019	**All pediod***<br>2001 to 2019
**CCT target population coverage**						
Low (0 – 29.9%)	1	1	1	1	1	1

© 2023 Cavalcanti DM et al. *JAMA Network Open.*

	UNDER-FIVE MORTALITY RATE BY DIFFERENT TIME SHOCK CONTROL					
	**No time Control**	**Fist 5 years** 2000 to 2004	**Second 5 years** 2005 to 2009	**Third 5 years** 2010 to 2014	**Last 5 years** 2015 to 2019	**All pediod*** 2001 to 2019
	[1.000-1.000]	[1.000-1.000]	[1.000-1.000]	[1.000-1.000]	[1.000-1.000]	[1.000-1.000]
Intermediate (30 – 69.9%)	0.901***	0.928***	0.907***	0.897***	0.903***	0.954***
	[0.892-0.910]	[0.914-0.942]	[0.899-0.916]	[0.882-0.912]	[0.889-0.918]	[0.946-0.962]
High (70 – 99.9%)	0.868***	0.893***	0.833***	0.828***	0.827***	0.915***
	[0.858-0.878]	[0.877-0.909]	[0.824-0.842]	[0.812-0.844]	[0.811-0.842]	[0.906-0.925]
Consolidated (100%)	0.785***	0.790***	0.689***	0.743***	0.747***	0.869***
	[0.779-0.792]	[0.777-0.803]	[0.684-0.694]	[0.732-0.755]	[0.736-0.757]	[0.861-0.878]
**Control Variables**						
Poverty rate (%)	1.106***	1.003	1.105***	1.110***	1.108***	1.058***
	[1.094-1.119]	[0.987-1.019]	[1.094-1.117]	[1.088-1.133]	[1.085-1.130]	[1.049-1.068]
Proportion of individuals older than 15 years who are illiterate (%)	1.036***	1.039***	1.057***	1.077***	1.043***	0.994
	[1.022-1.050]	[1.020-1.058]	[1.045-1.070]	[1.052-1.101]	[1.019-1.067]	[0.984-1.005]
Gini Index	1.082***	1.047***	0.991*	1.033***	1.024***	1.003
	[1.072-1.093]	[1.033-1.061]	[0.982-1.000]	[1.015-1.052]	[1.006-1.042]	[0.995-1.011]
Piped water	1.009	0.939***	0.976***	0.971***	0.989	1.017***
	[0.996-1.022]	[0.923-0.954]	[0.965-0.987]	[0.951-0.992]	[0.968-1.010]	[1.008-1.027]
Proportion of individuals living in households with adequate sanitation (%)	0.924***	0.884***	0.977***	0.905***	0.930***	0.943***
	[0.913-0.935]	[0.869-0.899]	[0.966-0.987]	[0.887-0.924]	[0.911-0.950]	[0.934-0.952]
Hospital bed rate per 1,000 population (%)	1.002	1.011	0.954***	1.005	0.997	1.002
	[0.987-1.018]	[0.991-1.032]	[0.941-0.967]	[0.979-1.031]	[0.972-1.023]	[0.991-1.014]
Rate of physicians per 1,000 population (%)	1.006	0.976***	0.957***	0.948***	0.968***	1.023***
	[0.994-1.018]	[0.961-0.992]	[0.947-0.968]	[0.930-0.967]	[0.949-0.988]	[1.013-1.032]
**Time Control**						
2000		1.275***				
		[1.253-1.298]				
2001		1.179***				0.947***
		[1.158-1.200]				[0.937-0.957]
2002		1.149***				0.943***
		[1.128-1.169]				[0.933-0.953]
2003		1.121***				0.911***
		[1.101-1.141]				[0.901-0.921]
2004		1.112***				0.905***
		[1.094-1.130]				[0.894-0.916]
2005			1.088***			0.901***
			[1.077-1.100]			[0.890-0.912]
2006			1.068***			0.877***
			[1.056-1.080]			[0.866-0.888]
2007			1.154***			0.855***
			[1.142-1.167]			[0.844-0.866]
2008			1.082***			0.827***
			[1.070-1.094]			[0.817-0.838]
2009			1.075***			0.820***
			[1.063-1.087]			[0.809-0.831]
2010				0.999		0.795***
				[0.978-1.021]		[0.784-0.806]
2011				0.978**		0.785***
				[0.957-0.999]		[0.774-0.796]
2012				0.928***		0.771***
				[0.908-0.949]		[0.761-0.782]
2013				0.928***		0.768***
				[0.908-0.949]		[0.757-0.778]
2014				0.888***		0.737***
				[0.868-0.908]		[0.727-0.747]
2015					0.823***	0.725***
					[0.804-0.842]	[0.715-0.735]
2016					0.914***	0.744***
					[0.894-0.935]	[0.734-0.755]
2017					0.868***	0.739***
					[0.848-0.888]	[0.729-0.750]
2018					0.869***	0.729***

© 2023 Cavalcanti DM et al. *JAMA Network Open.*

	UNDER-FIVE MORTALITY RATE BY DIFFERENT TIME SHOCK CONTROL					
	No time Control	Fist 5 years 2000 to 2004	Second 5 years 2005 to 2009	Third 5 years 2010 to 2014	Last 5 years 2015 to 2019	All pediod* 2001 to 2019
					[0.849-0.889]	[0.718-0.739]
2019					0.911***	0.755***
					[0.890-0.932]	[0.744-0.766]
**Number of observations**	**95,241**	**94,452**	**95,281**	**87,090**	**87,090**	**95,130**
**Number of municipalities**	**4,879**	**4,835**	**4,881**	**4,662**	**4,662**	**4,882**

Source: Author's data analysis for 95,352 observations - 4,881 municipalities in Brazil, Ecuador and Mexico, over 20 years (from 2000 to 2019).
Note: Data are in Rate Ratio (RR) coefficients (95% CI) unless otherwise specified. The confidence intervals are in parentheses. The symbols '***' , '**' and '*' denote significance at 1%, 5%, and 10% respectively.

eTable 16. Rate Ratios for the association between under five mortality rates and Conditional Cash Transference (CCT) target coverage, using fixed effect negative binomial and Poisson models; from the period 2000-19, in all municipalities in Brazil, Ecuador and Mexico.

	UNDER-FIVE MORTALITY RATE BY AGE GROUPS AND MODELS TYPE					
	Child Under 5 years		Infant Under 1 year		Neonatal 0 to 28 days	
	Negative Binomial	Poisson	Negative Binomial	Poisson	Negative Binomial	Poisson
**CCT target population coverage**						
Low (0 – 29.9%)	1	1	1	1	1	1
	[1.000-1.000]	[1.000-1.000]	[1.000-1.000]	[1.000-1.000]	[1.000-1.000]	[1.000-1.000]
Intermediate (30 – 69.9%)	0.876***	0.874***	0.916***	0.907***	0.846***	0.841***
	[0.869-0.883]	[0.869-0.880]	[0.907-0.925]	[0.900-0.915]	[0.835-0.857]	[0.831-0.851]
High (70 – 99.9%)	0.834***	0.830***	0.867***	0.863***	0.785***	0.776***
	[0.827-0.842]	[0.824-0.836]	[0.858-0.876]	[0.856-0.870]	[0.773-0.797]	[0.766-0.787]
Consolidated (100%)	0.752***	0.742***	0.716***	0.700***	0.683***	0.672***
	[0.747-0.757]	[0.738-0.746]	[0.710-0.721]	[0.696-0.704]	[0.675-0.691]	[0.664-0.679]
**Control Variables**						
Poverty rate (%)	1.073***	1.075***	1.112***	1.084***	1.004	1
	[1.063-1.083]	[1.066-1.083]	[1.100-1.124]	[1.075-1.093]	[0.988-1.020]	[0.985-1.015]
Proportion of individuals older than 15 years who are illiterate (%)	1.046***	1.042***	1.069***	1.017***	1.062***	1.064***
	[1.035-1.057]	[1.033-1.052]	[1.056-1.083]	[1.007-1.027]	[1.042-1.081]	[1.047-1.082]
Gini Index	1.075***	1.077***	0.992*	1.028***	1.086***	1.095***
	[1.066-1.083]	[1.070-1.084]	[0.982-1.001]	[1.020-1.036]	[1.071-1.101]	[1.082-1.109]
Piped water	0.978***	0.978***	0.968***	0.995	0.926***	0.913***
	[0.969-0.988]	[0.970-0.986]	[0.957-0.979]	[0.986-1.004]	[0.911-0.942]	[0.899-0.927]
Proportion of individuals living in households with inadequate sanitation (%)	0.906***	0.918***	0.966***	0.923***	0.881***	0.891***
	[0.897-0.915]	[0.910-0.925]	[0.955-0.977]	[0.915-0.931]	[0.866-0.896]	[0.877-0.904]
Hospital bed rate per 1,000 population (%)	1.006	1.004	0.957***	1.015***	1.008	1.015
	[0.994-1.018]	[0.994-1.014]	[0.944-0.970]	[1.004-1.026]	[0.987-1.028]	[0.996-1.034]
Rate of physicians per 1,000 population (%)	0.988**	0.986***	0.947***	0.987***	0.970***	0.970***
	[0.979-0.997]	[0.978-0.993]	[0.937-0.957]	[0.979-0.996]	[0.955-0.986]	[0.957-0.984]
Year binaries	Yes	Yes	Yes	Yes	Yes	Yes
**Number of observations**	**95,130**	**95,130**	**95,281**	**95,281**	**94,452**	**94,452**
**Number of municipalities**	**4,882**	**4,882**	**4,881**	**4,881**	**4,835**	**4,835**
**Akaike's information criterion (AIC)**	**398,886**	**406,098**	**385,777**	**399,098**	**260,821**	**262,863**
**Bayesian information criterion (BIC)**	**399,037**	**406,240**	**385,928**	**399,240**	**260,972**	**263,005**

Source: Author's data analysis for 95,352 observations - 4,881 municipalities in Brazil, Ecuador and Mexico, over 20 years (from 2000 to 2019).
Note: Data are in Rate Ratio (RR) coefficients (95% CI) unless otherwise specified. The confidence intervals are in parentheses. Time shocks are controls for specific years of economic crisis (2008, 2013, and 2015) and for specific years of changes in CCT programs (2003, 2004, and 2014). The symbols '***' , '**' and '*' denote significance at 1%, 5%, and 10% respectively.

eTable 17. Rate Ratios from the fixed effect negative binomial models for the association between under five mortality rates and Conditional Cash Transference (CCT) target coverage using categorical variables, dividing the models by poverty quartiles (heterogeneity analyses); from the period 2000-19, in all municipalities in Brazil, Ecuador and Mexico.

	UNDER-FIVE MORTALITY RATE BY POVERTY QUARTILE			
	1st quartile (0 - 5.8%)	2nd quartile (5.9 - 19.1%)	3rd quartile (19.1% - 36%)	4th quartile (36% - 100%)
**CCT target population coverage**				
Low (0 – 29.9%)	1	1	1	1

	[1.000-1.000]	[1.000-1.000]	[1.000-1.000]	[1.000-1.000]
Intermediate (30 – 69.9%)	0.922***	0.908***	0.891***	0.797***
	[0.879-0.968]	[0.899-0.918]	[0.872-0.911]	[0.777-0.817]
High (70 – 99.9%)	0.926***	0.920***	0.848***	0.743***
	[0.886-0.967]	[0.905-0.936]	[0.830-0.866]	[0.727-0.760]
Consolidated (100%)	0.866***	0.834***	0.832***	0.641***
	[0.831-0.903]	[0.821-0.846]	[0.812-0.852]	[0.628-0.655]
**Control Variables**				
Poverty rate (%)	1	1	1	1
	[1.000-1.000]	[1.000-1.000]	[1.000-1.000]	[1.000-1.000]
Proportion of individuals older than 15 years who are illiterate (%)	1.071**	1.136***	1.018*	1.027
	[1.014-1.131]	[1.109-1.163]	[0.998-1.039]	[0.987-1.069]
Gini Index	1.044***	1.116***	1.050***	0.962**
	[1.025-1.063]	[1.101-1.131]	[1.027-1.074]	[0.931-0.993]
Piped water	0.970*	0.938***	1.001	1.036***
	[0.935-1.005]	[0.915-0.963]	[0.980-1.022]	[1.010-1.062]
Proportion of individuals living in households with inadequate sanitation (%)	0.927***	0.867***	1.011	1.02
	[0.902-0.953]	[0.849-0.886]	[0.975-1.049]	[0.995-1.045]
Hospital bed rate per 1,000 population (%)	0.979	1.053***	0.926***	0.964**
	[0.954-1.005]	[1.028-1.079]	[0.895-0.958]	[0.934-0.994]
Rate of physicians per 1,000 population (%)	0.972**	0.999	0.927***	1.035***
	[0.946-0.999]	[0.976-1.022]	[0.907-0.948]	[1.013-1.058]
Year binaries	Yes	Yes	Yes	Yes
**Number of observations**	**23,664**	**23,597**	**23,366**	**23,570**
**Number of municipalities**	**2,194**	**3,023**	**2,981**	**2,112**

Source: Author's data analysis for 23,664 observations - 2,194 municipalities in Brazil, Ecuador and Mexico, over 20 years (from 2000 to 2019).

Note: Data are in Rate Ratio (RR) coefficients (95% CI) unless otherwise specified. The confidence intervals are in parentheses. Time shocks are controls for specific years of economic crisis (2008, 2013, and 2015) and for specific years of changes in CCT programs (2003, 2004, and 2014). The symbols '***' , '**' and '*' denote significance at 1%, 5%, and 10% respectively.

© 2023 Cavalcanti DM et al. *JAMA Network Open.*

eTable 18. Rate Ratios from the fixed effect negative binomial models for the association between under five mortality rates and Conditional Cash Transference (CCT) target coverage using categorical variables, dividing the models by countries (heterogeneity analyses); from the period 2000-19, in all municipalities in Brazil, Ecuador and Mexico.

	UNDER-FIVE MORTALITY RATE BY COUNTRY AND AGE GROUP														
	Neonatal<br>0 to 28 days			Post Neonatal<br>28 days to year			Infant<br>Under 1 year			Toddler<br>1 to 4 years			Child<br>Under 5 years		
	**Brazil**	**Ecuador**	**Mexico**	**Brazil**	**Ecuador**	**Mexico**	**Brazil**	**Ecuador**	**Mexico**	**Brazil**	**Ecuador**	**Mexico**	**Brazil**	**Ecuador**	**Mexico**
**CCT target population coverage**															
Low (0 – 29.9%)	1	1	1	1	1	1	1	1	1	1	1	1	1	1	1
	[1.000-1.000]	[1.000-1.000]	[1.000-1.000]	[1.000-1.000]	[1.000-1.000]	[1.000-1.000]	[1.000-1.000]	[1.000-1.000]	[1.000-1.000]	[1.000-1.000]	[1.000-1.000]	[1.000-1.000]	[1.000-1.000]	[1.000-1.000]	[1.000-1.000]
Intermediate (30 – 69.9%)	0.881***	0.947**	0.915***	0.839***	0.775***	0.904***	0.877***	0.870***	1.007	0.872***	0.804***	0.983	0.867***	0.855***	0.917***
	[0.865-0.898]	[0.900-0.997]	[0.900-0.931]	[0.817-0.861]	[0.739-0.813]	[0.887-0.921]	[0.862-0.892]	[0.837-0.905]	[0.992-1.022]	[0.843-0.901]	[0.756-0.854]	[0.956-1.010]	[0.854-0.880]	[0.826-0.885]	[0.906-0.929]
High (70 – 99.9%)	0.863***	0.857***	0.926***	0.770***	0.736***	0.896***	0.878***	0.805***	0.994	0.807***	0.795***	0.967*	0.826***	0.812***	0.920***
	[0.851-0.876]	[0.805-0.912]	[0.903-0.950]	[0.754-0.786]	[0.695-0.780]	[0.870-0.922]	[0.866-0.890]	[0.768-0.844]	[0.974-1.016]	[0.785-0.829]	[0.742-0.850]	[0.929-1.006]	[0.816-0.835]	[0.780-0.846]	[0.903-0.938]
Consolidated (100%)	0.753***	0.747***	0.956***	0.654***	0.676***	0.864***	0.692***	0.711***	0.988	0.709***	0.792***	0.963	0.717***	0.738***	0.926***
	[0.746-0.761]	[0.690-0.809]	[0.927-0.987]	[0.645-0.663]	[0.625-0.731]	[0.833-0.895]	[0.686-0.698]	[0.668-0.756]	[0.963-1.013]	[0.696-0.722]	[0.722-0.869]	[0.918-1.011]	[0.711-0.722]	[0.699-0.778]	[0.905-0.948]
**Control Variables**															
Poverty rate (%)	1.089***	1.225***	1.263***	0.975***	1.088**	1.085***	1.073***	1.193***	1.062***	1.051***	1.765***	1.049	1.049***	1.259***	1.181***
	[1.076-1.103]	[1.135-1.323]	[1.202-1.327]	[0.958-0.993]	[1.010-1.173]	[1.021-1.152]	[1.060-1.086]	[1.123-1.268]	[1.020-1.106]	[1.026-1.076]	[1.575-1.977]	[0.970-1.135]	[1.039-1.060]	[1.191-1.331]	[1.138-1.227]
Proportion of individuals older than 15 years who are illiterate	1.012	0.903*	1.107***	1.031***	1.101**	1.133***	1.071***	0.987	1.086***	1.040***	1.362***	1.079***	1.019***	1.093***	1.113***
	[0.996-1.028]	[0.814-1.002]	[1.076-1.138]	[1.007-1.055]	[1.001-1.211]	[1.097-1.171]	[1.055-1.087]	[0.914-1.065]	[1.063-1.110]	[1.009-1.071]	[1.231-1.507]	[1.034-1.125]	[1.006-1.031]	[1.025-1.164]	[1.090-1.136]
Gini Index	1.033***	0.832	1.110***	1.063***	1.017	1.132***	0.980***	0.935	1.060***	1.044***	1.171	1.046***	1.042***	1.029	1.108***
	[1.020-1.046]	[0.625-1.107]	[1.090-1.130]	[1.044-1.083]	[0.772-1.339]	[1.109-1.156]	[0.969-0.992]	[0.751-1.164]	[1.044-1.076]	[1.019-1.069]	[0.883-1.552]	[1.018-1.075]	[1.031-1.052]	[0.855-1.237]	[1.094-1.124]
Piped water	1	0.640***	1.078***	0.888***	0.581***	1.009	0.931***	0.614***	1.040***	0.941***	0.393***	1.035*	0.958***	0.555***	1.048***
	[0.986-1.014]	[0.550-0.745]	[1.049-1.107]	[0.871-0.906]	[0.495-0.682]	[0.979-1.041]	[0.918-0.943]	[0.543-0.694]	[1.018-1.062]	[0.916-0.966]	[0.331-0.468]	[0.997-1.075]	[0.947-0.968]	[0.499-0.618]	[1.027-1.069]
Proportion of individuals living in households with inadequate sanitation (%)	0.932***	0.901	1.051*	0.878***	0.778***	0.97	0.962***	0.845**	1.037*	0.893***	0.736***	1.036	0.909***	0.820***	1.015
	[0.920-0.943]	[0.746-1.090]	[0.996-1.109]	[0.862-0.894]	[0.653-0.926]	[0.921-1.023]	[0.950-0.973]	[0.735-0.971]	[0.999-1.077]	[0.872-0.914]	[0.619-0.874]	[0.979-1.095]	[0.900-0.918]	[0.729-0.922]	[0.980-1.052]
Hospital bed rate per 1,000 population (%)	0.995	0.814***	0.97	1.014	0.886**	0.976	0.962***	0.847***	0.968**	1.025	0.789***	0.935**	1.005	0.838***	0.967**
	[0.979-1.012]	[0.728-0.910]	[0.933-1.008]	[0.990-1.038]	[0.798-0.984]	[0.934-1.020]	[0.947-0.977]	[0.779-0.922]	[0.940-0.998]	[0.993-1.057]	[0.706-0.880]	[0.883-0.989]	[0.992-1.018]	[0.780-0.900]	[0.939-0.995]
Rate of physicians per 1,000 population (%)	0.992	0.991	1.067***	0.963***	0.863***	1.038**	0.935***	0.925**	1.027**	0.952***	0.856***	1.041*	0.978***	0.893***	1.051***
	[0.979-1.006]	[0.909-1.080]	[1.037-1.097]	[0.946-0.982]	[0.799-0.932]	[1.004-1.073]	[0.923-0.946]	[0.869-0.985]	[1.005-1.050]	[0.929-0.975]	[0.789-0.929]	[0.999-1.084]	[0.968-0.989]	[0.848-0.941]	[1.029-1.073]
Year binaries	Yes	Yes	Yes	Yes	Yes	Yes	Yes	Yes	Yes	Yes	Yes	Yes	Yes	Yes	Yes
**Number of observations**	**73,259**	**2,968**	**19,014**	**72,500**	**2,968**	**18,984**	**73,299**	**2,968**	**19,014**	**70,440**	**2,968**	**13,682**	**73,322**	**2,794**	**19,014**
**Number of municipalities**	**3,665**	**149**	**1,065**	**3,627**	**149**	**1,059**	**3,667**	**149**	**1,065**	**3,524**	**149**	**989**	**3,668**	**149**	**1,065**

Source: Author's data analysis for 73,322 observations - 3,668 municipalities in Brazil, Ecuador and Mexico, over 20 years (from 2000 to 2019).
Note: Data are in Rate Ratio (RR) coefficients (95% CI) unless otherwise specified. The confidence intervals are in parentheses. Time shocks are controls for specific years of economic crisis (2008, 2013, and 2015) and for specific years of changes in CCT programs (2003, 2004, and 2014). The symbols '***' , '**' and '*' denote significance at 1%, 5%, and 10% respectively.

eTable 19.. Rate Ratios from the fixed effect negative binomial models by age groups for the association between mortality rates and Conditional Cash Transfers (CCT) coverage, with population weight control by municipality.

	**MORTALITY**									
	**Neonatal** 0 to 28 days		**Post-Neonatal** 28 days to 1 year		**Infant** Under 1 year		**Toddler** 1 to 4 years		**Child** Under 5 years	
	With Weight Control	No Weight Control	With Weight Control	No Weight Control	With Weight Control	No Weight Control	With Weight Control	No Weight Control	With Weight Control	No Weight Control
**CCT target population coverage**										
Low (0 – 29.9%)	1	1	1	1	1	1	1		1	1
Intermediate (30 – 69.9%)	0.882***	0.897***	0.866***	0.849***	1.025***	0.926***	0.951***	0.886***	0.883***	0.880***
	[0.882 – 0.882]	[0.888 – 0.907]	[0.865 – 0.866]	[0.838 – 0.860]	[1.025 – 1.025]	[0.917 – 0.935]	[0.951 – 0.951]	[0.871 – 0.901]	[0.883 – 0.883]	[0.873 – 0.887]
High (70 – 99.9%)	0.856***	0.870***	0.834***	0.789***	1.107***	0.880***	0.866***	0.826***	0.850***	0.840***
	[0.856 – 0.856]	[0.861 – 0.880]	[0.834 – 0.834]	[0.777 – 0.801]	[1.107 – 1.107]	[0.871 – 0.889]	[0.866 – 0.866]	[0.810 – 0.842]	[0.850 – 0.850]	[0.832 – 0.847]
Consolidated (100%)	0.785***	0.795***	0.771***	0.687***	0.733***	0.727***	0.794***	0.744***	0.782***	0.757***
	[0.785 – 0.785]	[0.788 – 0.802]	[0.771 – 0.771]	[0.679 – 0.695]	[0.733 – 0.733]	[0.721 – 0.733]	[0.794 – 0.794]	[0.733 – 0.755]	[0.782 – 0.782]	[0.752 – 0.762]
**Control variables**										
Poverty rate (%)	1.179***	1.104***	1.081***	1.005	1.304***	1.110***	1.262***	1.112***	1.173***	1.072***
	[1.179 – 1.179]	[1.091 – 1.117]	[1.081 – 1.081]	[0.989 – 1.020]	[1.304 – 1.304]	[1.098 – 1.122]	[1.262 – 1.262]	[1.089 – 1.135]	[1.173 – 1.173]	[1.063 – 1.082]
Proportion of individuals older than 15 years who are illiterate (%)	0.963***	1.032***	1.055***	1.062***	1.301***	1.069***	1.013***	1.069***	0.994***	1.047***
	[0.963 – 0.963]	[1.018 – 1.046]	[1.055 – 1.055]	[1.043 – 1.081]	[1.301 – 1.301]	[1.056 – 1.082]	[1.013 – 1.013]	[1.045 – 1.093]	[0.994 – 0.994]	[1.036 – 1.058]
Gini Index	1.147***	1.071***	1.110***	1.085***	0.863***	0.998**	1.070***	1.030***	1.128***	1.072***
	[1.147 – 1.147]	[1.060 – 1.082]	[1.110 – 1.110]	[1.070 – 1.099]	[0.863 – 0.863]	[0.979 – 0.998]	[1.070 – 1.070]	[1.012 – 1.048]	[1.128 – 1.128]	[1.064 – 1.081]
Piped water	1.018***	1.013**	0.998***	0.926***	0.937***	0.970***	1.016***	0.974**	1.006***	0.979***
	[1.018 – 1.018]	[1.001 – 1.026]	[0.998 – 0.998]	[0.911 – 0.942]	[0.937 – 0.937]	[0.959 – 0.981]	[1.016 – 1.016]	[0.954 – 0.995]	[1.006 – 1.006]	[0.969 – 0.988]
Proportion of individuals living in households with inadequate sanitation (%)	0.898***	0.928***	0.898***	0.881***	1.352***	0.966***	0.907***	0.912***	0.902***	0.907***
	[0.898 – 0.898]	[0.917 – 0.939]	[0.898 – 0.898]	[0.867 – 0.896]	[1.352 – 1.352]	[0.956 – 0.977]	[0.907 – 0.907]	[0.894 – 0.932]	[0.902 – 0.902]	[0.898 – 0.915]
Hospital bed rate per 1,000 population (%)	0.971***	1.001	0.892***	1.008	0.562***	0.959***	0.826***	1.004	0.917***	1.006
	[0.971 – 0.971]	[0.986 – 1.016]	[0.892 – 0.893]	[0.988 – 1.028]	[0.562 – 0.562]	[0.946 – 0.972]	[0.826 – 0.826]	[0.979– 1.030]	[0.917 – 0.917]	[0.995 – 1.018]
Rate of physicians per 1,000 population (%)	1.049***	1.09	0.899***	0.971***	0.728***	0.949***	0.851***	0.953***	0.974***	0.989**
	[1.049 – 1.049]	[0.997 – 1.021]	[0.899 – 0.899]	[0.956 – 0.986]	[0.728 – 0.728]	[0.939 – 0.959]	[0.851 – 0.851]	[0.935 – 0.972]	[0.974 – 0.974]	[0.979 – 0.998]
Year binaries	Yes	Yes	Yes	Yes	Yes	Yes	Yes	Yes	Yes	Yes
**Number of observations**	**95,242**	**95,242**	**94,452**	**94,452**	**95,281**	**95,281**	**87,090**	**87,090**	**95,130**	**95,130**
**Number of municipalities**	**4,879**	**4,879**	**4,835**	**4,835**	**4,881**	**4,881**	**4,662**	**4,662**	**4,882**	**4,882**

Note: Data are in Rate Ratio (RR) coefficients (95% CI) unless otherwise specified. The confidence intervals are in parentheses. Time shocks are controls for specific years of economic crisis (2008, 2013, and 2015) and for specific years of changes in CCT programs (2003, 2004, and 2014). The symbols '***' , '**' and '*' denote significance at 1%, 5%, and 10% respectively.

© 2023 Cavalcanti DM et al. *JAMA Network Open.*

eTable 20.. Correlation matrix of continuous variables.

	U5MR	CCT target	Poverty	Illiteracy rate	Gini	Water	Sewage	Hospital bed rate	Doctor rate
U5MR	1.00								
CCT target coverage	-0.04	1.00							
Poverty	0.06	-0.31	1.00						
Illiteracy rate	0.05	-0.10	0.76	1.00					
Gini	0.03	-0.31	0.22	0.26	1.00				
Water	-0.02	0.18	-0.41	-0.38	-0.21	1.00			
Sewage	0.01	0.14	0.37	0.22	-0.11	0.01	1.00		
Hospital bed rate	0.00	-0.06	-0.20	-0.10	0.19	0.03	-0.26	1.00	
Doctor rate	-0.02	0.02	-0.30	-0.31	0.08	0.16	-0.31	0.30	1.00

eTable 21.Correlations Matrix of the parameter estimates of the main model.

e(V)	CCT target (Interme-diate)	CCT target (High)	CCT target (Consolida-ted)	Poverty rate	Illiteracy rate	Gini Index	Piped water	Adequate sanitation	Hospital bed rate	Doctor rate	Year 2002	Year 2004	Year 2008	Year 2013	Year 2015
death_0_4															
CCT_target (Intermediate)	**1.00**														
CCT_target (High)	0.32	**1.00**													
CCT_target (Consolidated)	0.27	0.40	**1.00**												
Poverty rate	0.05	0.09	0.31	**1.00**											
Illiteracy rate	0.01	0.03	0.05	-0.23	**1.00**										
Gini Index	0.09	0.08	0.16	0.00	-0.09	**1.00**									
Piped water	0.02	0.02	-0.06	0.00	0.08	0.08	**1.00**								
Adequate sanitation	-0.01	-0.04	-0.22	0.21	0.08	-0.03	-0.08	**1.00**							
Hospital bed rate	0.01	0.03	0.07	0.00	0.00	-0.06	-0.01	0.03	**1.00**						
Doctor rate	0.00	0.00	-0.03	0.00	0.04	0.01	-0.06	-0.02	-0.02	**1.00**					
Year (2002)	0.13	0.17	0.25	-0.02	0.01	-0.07	0.02	0.02	0.00	0.01	**1.00**				
Year (2004)	-0.28	-0.05	0.11	0.04	-0.02	-0.06	0.02	0.03	0.01	0.03	0.09	**1.00**			
Year (2008)	0.00	-0.04	-0.11	0.00	-0.03	0.01	0.02	0.00	-0.01	0.02	0.03	0.04	**1.00**		
Year (2013)	-0.04	-0.03	-0.08	0.02	0.01	0.06	-0.01	0.00	0.01	0.00	0.01	0.04	0.07	**1.00**	
Year (2015)	-0.02	0.01	-0.05	0.02	0.02	0.08	-0.03	-0.04	0.00	-0.03	0.02	0.03	0.07	0.08	**1.00**

eTable 22. Variance Inflation Factor (VIF) of the main model.

Variable	VIF	1/VIF
CCT_target (Intermediate)	1.66	0.60
CCT_target (High)	1.49	0.67
CCT_target (Consolidated)	2.17	0.46
Poverty rate	1.93	0.52
Illiteracy rate	1.68	0.59
Gini Index	1.28	0.78
Piped water	1.15	0.87
Adequate sanitation	1.43	0.70
Hospital bed rate	1.27	0.79
Doctor rate	1.24	0.81
Year (2002)	1.17	0.85

© 2023 Cavalcanti DM et al. *JAMA Network Open.*

Variable	VIF	1/VIF
Year (2004)	1.22	0.82
Year (2008)	1.03	0.97
Year (2013)	1.03	0.97
Year (2015)	1.03	0.97
**Mean VIF**	**1.38**	

eTable 23. Rate Ratios from the fixed effect negative binomial models for the association between under five mortality rates and Conditional Cash Transference (CCT) target coverage using categorical variables, by different causes of deaths (placebo test); from the period 2000-19, in all municipalities in Brazil, Ecuador and Mexico.

	UNDER-FIVE MORTALITY RATE BY SPECIFIC CAUSES OF DEATHS						
	Diarrhoeal diseases	Malnutrition	Tuberculosis	Lower respiratory infections	Malaria	HIV/AIDS	External cause
**CCT target population coverage**							
Low (0 – 29.9%)	1	1	1	1	1	1	1
Intermediate (30 – 69.9%)	0.738***	0.640***	0.921	0.845***	0.911	0.690***	0.994
	[0.707-0.770]	[0.608-0.675]	[0.702-1. 208]	[0.816-0. 875]	[0.685-1.211]	[0.587-0.812]	[0.954-1.036]
High (70 – 99.9%)	0.636***	0.503***	0.661***	0.792***	0.808*	0.599***	1.040
	[0.609-0.664]	[0.475-0.533]	[0.487-0. 897]	[0.764-0.821]	[0.636-1.026]	[0.504-0.713]	[0.982-1.102]
Consolidated (100%)	0.414***	0.327***	0.618***	0.662***	0.764***	0.323***	1.006
	[0.399-0.430]	[0.311-0.345]	[0.484-0. 790]	[0.645-0.680]	[0.630-0.927]	[0.284-0.368]	[0.93901.077]
**Control variables**	Yes	Yes	Yes	Yes	Yes	Yes	Yes
Year binaries	Yes	Yes	Yes	Yes	Yes	Yes	Yes
**Number of observations**	**65,746**	**56,830**	**7,453**	**71,250**	**10,290**	**15,861**	**41,195**
**Number of municipalities**	**3,528**	**3,011**	**380**	**3,947**	**526**	**807**	**2,822**

Note: Data are in Rate Ratio (RR) coefficients (95% CI) unless otherwise specified. The confidence intervals are in parentheses. Time shocks are controls for specific years of economic crisis (2008, 2013, and 2015) and for specific years of changes in CCT programs (2003, 2004, and 2014). The symbols '***' , '**' and '*' denote significance at 1%, 5%, and 10% respectively.

eTable 24. Rate Ratios from the fixed effect negative binomial models by age groups for the association between mortality rates and Conditional Cash Transfers (CCT) coverage, with control variables categorized by quartiles.

	MORTALITY				
	**Neonatal**	**Post-Neonatal**	**Infant**	**Toddler**	**Child**
	0 to 28 days	28 days to 1 year	Under 1 year	1 to 4 years	Under 5 years
**CCT target population coverage**					
Low (0 – 29.9%)	1	1	1	1	1
Intermediate (30 – 69.9%)	0.911***	0.862***	0.940***	0.914***	0.895***
	[0.902 – 0.921]	[0.850 – 0.874]	[0.930 – 0.949]	[0.898 – 0.930]	[0.888 – 0.903]
High (70 – 99.9%)	0.892***	0.809***	0.898***	0.864***	0.863***
	[0.881 – 0.902]	[0.796 – 0.821]	[0.889 – 0.908]	[0.847 – 0.882]	[0.855 – 0.871]
Consolidated (100%)	0.838***	0.735***	0.779***	0.818***	0.806***
	[0.829 – 0.847]	[0.724 – 0.745]	[0.771 – 0.787]	[0.802 – 0.833]	[0.799 – 0.813]
**Control Variables**					
**Poverty rate**					
1st quartile	1	1	1	1	1
2nd quartile	1.048***	0.997	1.049***	1.066***	1.032***
	[1.037 – 1.059]	[0.982 – 1.012]	[1.038 – 1.059]	[1.045 – 1.088]	[1.023 – 1.040]
3rd quartile	1.143***	0.974**	1.131***	1.164***	1.085***
	[1.124 – 1.163]	[0.952 – 0.997]	[1.113 – 1.149]	[1.128 – 1.200]	[1.071 – 1.100]

	MORTALITY				
	**Neonatal**	**Post-Neonatal**	**Infant**	**Toddler**	**Child**
	0 to 28 days	28 days to 1 year	Under 1 year	1 to 4 years	Under 5 years
4th quartile	1.175***	1.184***	1.271***	1.369***	1.189***
	[1.147 – 1.204]	[1.146 – 1.223]	[1.243 – 1.299]	[1.313 – 1.428]	[1.167 – 1.212]
**Illiteracy rate**					
1st quartile	1	1	1	1	1
2nd quartile	1.057***	1.053***	1.106***	1.091***	1.064***
	[1.045 – 1.069]	[1.037 – 1.070]	[1.095 – 1.118]	[1.070 – 1.112]	[1.055 – 1.074]
3rd quartile	1.080***	1.102***	1.162***	1.151***	1.100***
	[1.061 – 1.100]	[1.076 – 1.129]	[1.143 – 1.181]	[1.117 – 1.187]	[1.085 – 1.115]
4th quartile	1.106***	1.174***	1.221***	1.217***	1.144***
	[1.078 – 1.135]	[1.134 – 1.215]	[1.192 – 1.250]	[1.165 – 1.271]	[1.122 – 1.168]
**Gini Index**					
1st quartile	1	1	1	1	1
2nd quartile	1.064***	1.030***	0.973***	1.038***	1.053***
	[1.053 – 1.075]	[1.015 – 1.044]	[0.963 – 0.983]	[1.020 – 1.057]	[1.044 – 1.061]
3rd quartile	1.110***	1.089***	0.952***	1.037***	1.098***
	[1.095 – 1.125]	[1.069 – 1.108]	[0.940 – 0.965]	[1.013 – 1.062]	[1.087 – 1.110]
4th quartile	1.136***	1.135***	0.921***	1.065***	1.131***
	[1.117 – 1.155]	[1.110 – 1.161]	[0.907 – 0.936]	[1.034 – 1.096]	[1.116 – 1.146]
**Piped water**					
1st quartile	1	1	1	1	1
2nd quartile	1.002	0.940***	0.951***	0.993	0.978***
	[0.985 – 1.021]	[0.919 – 0.962]	[0.936 – 0.966]	[0.965 – 1.021]	[0.964 – 0.991]
3rd quartile	1.037***	0.931***	0.959***	1.006	0.995
	[1.015 – 1.059]	[0.906 – 0.957]	[0.941 – 0.977]	[0.972 – 1.040]	[0.979 – 1.011]
4th quartile	1.02	0.910***	0.898***	0.985	0.973***
	[0.996 – 1.045]	[0.882 – 0.939]	[0.879 – 0.917]	[0.947 – 1.024]	[0.956 – 0.991]
**Adequate sanitatiton**					
1st quartile	1	1	1	1	1
2nd quartile	0.941***	0.941***	0.917***	0.942***	0.938***
	[0.931 – 0.952]	[0.926 – 0.956]	[0.908 – 0.927]	[0.923 – 0.962]	[0.929 – 0.946]
3rd quartile	0.878***	0.862***	0.908***	0.885***	0.866***
	[0.863 – 0.892]	[0.843 – 0.882]	[0.894 – 0.921]	[0.860 – 0.911]	[0.855 – 0.877]
4th quartile	0.786***	0.718***	0.848***	0.817***	0.753***
	[0.768 – 0.805]	[0.696 – 0.741]	[0.830 – 0.866]	[0.785 – 0.851]	[0.739 – 0.767]
**Hospital Bed rate**					
1st quartile	1	1	1	1	1
2nd quartile	0.950***	0.991	0.953***	0.989	0.966***
	[0.932 – 0.969]	[0.967 – 1.015]	[0.935 – 0.970]	[0.958 – 1.021]	[0.952 – 0.981]
3rd quartile	0.946***	0.984	0.909***	0.984	0.964***
	[0.923 – 0.969]	[0.954 – 1.016]	[0.890 – 0.930]	[0.944 – 1.024]	[0.946 – 0.982]
4th quartile	0.968**	1.018	0.934***	0.989	0.988
	[0.942 – 0.995]	[0.982 – 1.055]	[0.911 – 0.957]	[0.944 – 1.037]	[0.968 – 1.009]
**Doctor rate**					
1st quartile	1	1	1	1	1
2nd quartile	1	0.99	0.981***	0.996	0.999

© 2023 Cavalcanti DM et al. *JAMA Network Open.*

	MORTALITY				
	**Neonatal**	**Post-Neonatal**	**Infant**	**Toddler**	**Child**
	0 to 28 days	28 days to 1 year	Under 1 year	1 to 4 years	Under 5 years
	[0.987 – 1.013]	[0.973 – 1.006]	[0.969 – 0.992]	[0.975 – 1.017]	[0.989 – 1.009]
3rd quartile	1.021***	0.994	0.968***	0.978*	1.009
	[1.005 – 1.037]	[0.974 – 1.015]	[0.954 – 0.982]	[0.953 – 1.004]	[0.997 – 1.022]
4th quartile	1.012	0.943***	0.886***	0.920***	0.979***
	[0.993 – 1.031]	[0.920 – 0.967]	[0.871 – 0.901]	[0.891 – 0.949]	[0.965 – 0.993]
Year binaries	Yes	Yes	Yes	Yes	Yes
**Number of observations**	**95,241**	**94,452**	**95,281**	**87,090**	**95,130**

Note: Data are in Rate Ratio (RR) coefficients (95% CI) unless otherwise specified. The confidence intervals are in parentheses. Time shocks are controls for specific years of economic crisis (2008, 2013, and 2015) and for specific years of changes in CCT programs (2003, 2004, and 2014). The symbols '***' , '**' and '*' denote significance at 1%, 5%, and 10% respectively.

4.5. Deaths averted by CCT programs during 2000-19

To simulate deaths avoided due to CCT programs in 2000-19 period, we predicted coefficient $E(Y_{it} \mid X)$, here X represents the set of covariates including the interventions, and Y_{it} are the under-5 mortality rate at municipality *i*, in year *t*. Thus, the Monte Carlo methodology was used to get more accurate results compared with conventional methods such as the use of the normal distribution. It can be summarized in the following steps

1. Predict the intervention values for the retrospective period (2000-2019) initially using the same coefficients as the main model (Table 2);
2. Simulate a new Y_{it} from the negative binomial distribution using the estimated parameters from the retrospective study, changing only the CCT coefficient to rescue the baseline (0% coverage) and compared with real deaths, making the difference between them and adding them up over the different years;
3. Get the predictions $E(Y_{it} \mid X)$ using the new simulated variable Y_{it}, here X represents the set of covariates including the interventions.
4. Get back to step 1.

The algorithm ended when the number of desired Monte Carlo simulations M is reached. For each outcome, 10,000 simulations were performed, chosen based on the stabilization of the estimates.

The eTable 25 show this simulation, in which CCT programs avoiding 738,919 (95% CI: 695,641-782,104) child deaths between 2000-19 in the hypothetical case these programs did not exist (0% coverage).

eTable 25. Child death avoided by Conditional Cash Transference (CCT) coverage during 2000-19.

**Averted deaths until 2019**		
Estimate	LI	LS
738,919	695,641	782,104

© 2023 Cavalcanti DM et al. *JAMA Network Open.*

PART III – FORECASTING ANALYSIS

5. Description of the forecasting methodology

The following section provides details of the forecasting process in accordance with standard international modeling reporting guidelines (ISPOR-SMDM). The modeling approach adopted for this study was developed based on two stages.

In the first stage, a synthetic cohort of all Brazilian, Ecuadorian and Mexican municipalities for the period 2020-2030 was created as an extension of a longitudinal dataset of 8,103 municipalities for 2000-2019 obtained from the sources detailed in eTable 1. Simulated municipality-specific trends for poverty rates and the other demographic and socioeconomic variables were obtained according to economic crisis scenarios for the years 2020-2030. CCT coverage were simulated according to social protection policy response scenarios options.

In the second stage, for each year and each municipality, the mortality rate for all the municipalities was estimated as the outcome of the same multivariate fixed effects regressions, using the forecast demographic, socioeconomic and exposure variables (CCT coverage) as input values.

6. Purpose of the forecasting and its applications

The developed model had the overall purpose to simulate the effects of socioeconomic and policy coverage changes on health outcomes in BEM countries using ecologic-level data and - when available - retrospective ecologic datasets. Elements of flexibility have been introduced in the code to allow simulation of different sets of variables and different regression models.

7. Inputs, outputs, and other parameters

7.1. Scenarios of poverty and coverage of CCT programs

In order to develop forecasting, exponential functions were used to simulate the covariates behavior for the next 11 years (2020-2030). Regarding the poverty rate, an increasing scenario was considered for the first years (economic crisis period). This is described by the equation,

$$x_t = x_{2019} + c_1 x_{2019}(1 - \exp(-k_1 t)), \quad (2)$$

for the remaining years, we consider the exponential decay,

$$x_t = x_{2019} - c_2 x_{2019}(1 - \exp(-k_2 t)), \quad (3)$$

where the parameters c_1, k_1, c_2, k_2 were settled according to different available sources.

With respect to the intervention variables (CCT) under the mitigation scenario, they were considered as having the same behavior of the poverty rate (mitigation effect) during the economic crisis[19]. For the post - crisis, the interventions were simulated using the exponential decay in equation (3). The decreasing rate k2 in this case, was settled as half the poverty to simulate the transition period between the crisis and recovery scenarios.

Under the austerity scenarios, the interventions x_t, austerity were considered to follow an exponential decay which shows directly the percentage of decrease per year, this allowed to support the policies simulation according to situations that concerns the BEM countries government expenditure.[20] The equation below describes this dynamic.

$$x_{t,aust} = x_{t,aust}\,(1 - p)^t$$

where p is the percentage of decreasing for each intervention and t refers to the year.

We simulated three economic crisis scenarios using the increase in the poverty rates, which was calculated using the data from 2000 to 2019. Poverty is defined as the percentage of households below the eligibility condition for the CCT program. The magnitude of the economic crisis is represented by the percentage variation of the poverty rate from 2019. Recent reports show that the acute increase of poverty rate from 2020 to 2021 (about 22,7%) is significantly higher compared to the annual poverty increase used as economic crises scenarios in this study.[21]

The economic crisis scenarios considered in this analysis were simulated as follows:

- Shorter Economic Crisis scenario: A milder and shorter economic crisis, with an increase in poverty rates for the first three years (2020 - 2022). This behavior was generated using equation (2). On the other side, For the post-crisis period (2023 -2030), poverty rates were simulated by using equation (3), the parameters describing the poverty by country for this first scenario are described in eTable 26.

eTable 26.. Parameters describing poverty during the first economic crisis scenario.

Parameter	Country	Increasing period	Decreasing period
k	Brazil	0.225	0.2
	Mexico	0.02	0.03
	Ecuador	0.15	0.3
c	Brazil	0.6	0.53
	Mexico	0.025	0.05
	Ecuador	0.2	0.1

- Medium Economic Crisis scenario: A medium economic crisis with a larger increase in the poverty rate for the first 5 years (2020-2024). This behavior was generated using equation (2). As in the first scenario, For the post-crisis period (2025 -2030), the poverty rates were simulated by using equation (3), the parameters describing the poverty by country for this scenario are described in Web-Table 27.

eTable 27.. Parameters describing poverty during the first economic crisis scenario.

Parameter	Country	Increasing period	Decreasing period
k	Brazil	0.45	0.2
	Mexico	0.04	0.03
	Ecuador	0.3	0.3
c	Brazil	1.2	0.53
	Mexico	0.05	0.05
	Ecuador	0.4	0.1

- Longer Economic Crisis scenario: A longer economic crisis was created using similar parameters as the Medium Economic Crisis scenarios, but with an increase sustained over 7 years (from 2015 to 2021).

In response to the economic crisis, three policy responses were considered in the main analysis:

- Mitigation scenario: a mitigation strategy with a proportional behavior of the CCT programs to the poverty scenarios, during the corresponding simulated economic crisis. In this case, these interventions were generated in the same way as poverty rates, considering the same equation and parameters according to each period and scenario.
- Baseline scenario: derived from a validated model - already employed in previous studies[5,6] - that projected the effects of the current fiscal austerity measures due to the *Emenda Constitucional* 95 (EC95) on the coverage of the three interventions. This scenario was simulated according to the equation (4) considering a percentage of decrease of 5%, as in previous studies.[5,6]
- Severe Austerity scenario: based on the reduction of CCT proportional to the reduction of government expenditure on social protection observed from 2014-2019. [24] This scenario was simulated according to the equation (4) considering a percentage of decrease of 9.8%. This

© 2023 Cavalcanti DM et al. *JAMA Network Open.*

percentage was derived from the reduction of government expenditure on social protection (excluding cash transfer programs) observed from 2014-2019.[24]

The eFigure 19 and 20 show the behavior of the poverty scenarios.

eFigure 19. Forecasting poverty scenarios.

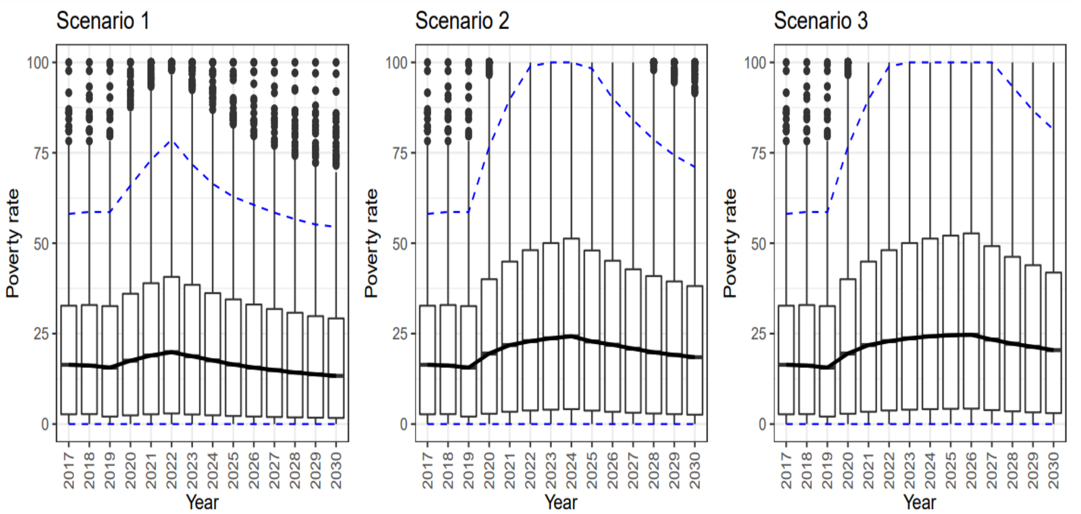

eFigure 20. Potential scenarios of economic crisis and poverty, alternative responses of Conditional Cash Transfer programs coverage, and related child mortality rates predictions for Brazil, Ecuador and Mexico up to 2030.

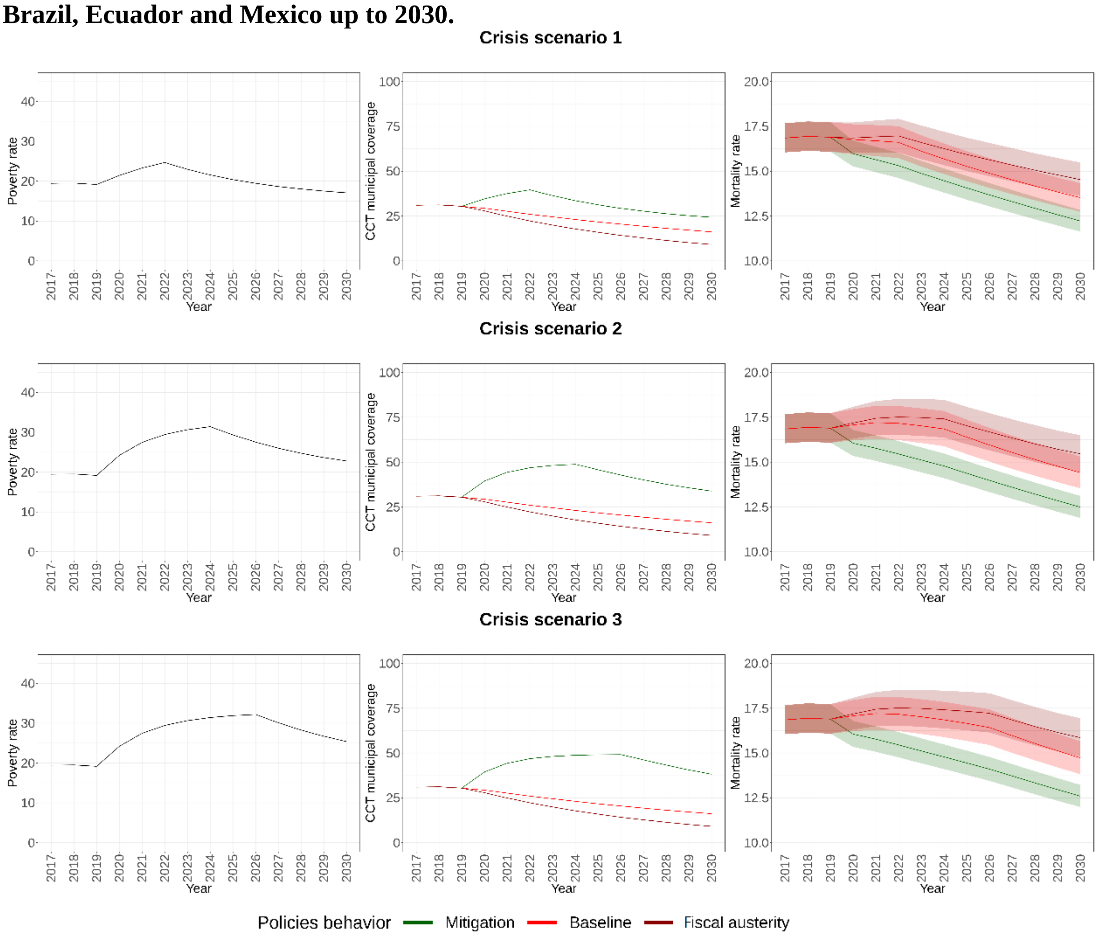

8. Prediction methodology

To generate predictions and confidence intervals for each response Y_{it}, the Monte Carlo methodology was used. This procedure allows to get more accurate results compared with conventional methods such as the use of the normal distribution. It can be summarized in the following steps

5. Simulate the intervention values for the forecasting period (2020 -2030) using the mitigation and austerity scenarios settled in previous sections. Also simulate the control covariates using equations (2) and (3) and following their trend.
6. Simulate a new Y_{it} from the negative binomial distribution using the estimated parameters from the retrospective study and the forecasted covariates.
7. Get the predictions $E(Y_{it} \mid X)$ using the new simulated variable Y_{it}, here X represents the set of covariates including the interventions.
8. Get back to step 1.

The algorithm ended when the number of desired Monte Carlo simulations M is reached. The predictions and confidence interval estimated for Y_{it} will be the mean and the percentiles 2.5% and 97.5% of the M simulations respectively. For each outcome and each scenario, 10,000 simulations were performed, allowing parameter values to vary in each simulation cycle according to their assumed underlying distribution. The number 10,000 was chosen based on the stabilization of the estimates.

8.1. External validation of each model

The external validation of the model was undertaken comparing the overall national mortality rate (computed for each municipality) forecasted using microsimulations, with the official Brazilian mortality estimates (overall) during the years 2010-2019, which are the most up-to-date available, and estimating the linear regression and the correlation coefficients (R2) of predicted vs observed values, as shown in eFigure 21.

eFigure 21. Linear regression and correlation coefficient (R2) of predicted vs observed values, and trend of the simulated overall mortality rate vs the official Brazilian mortality rate estimates for the period 2010-2019.

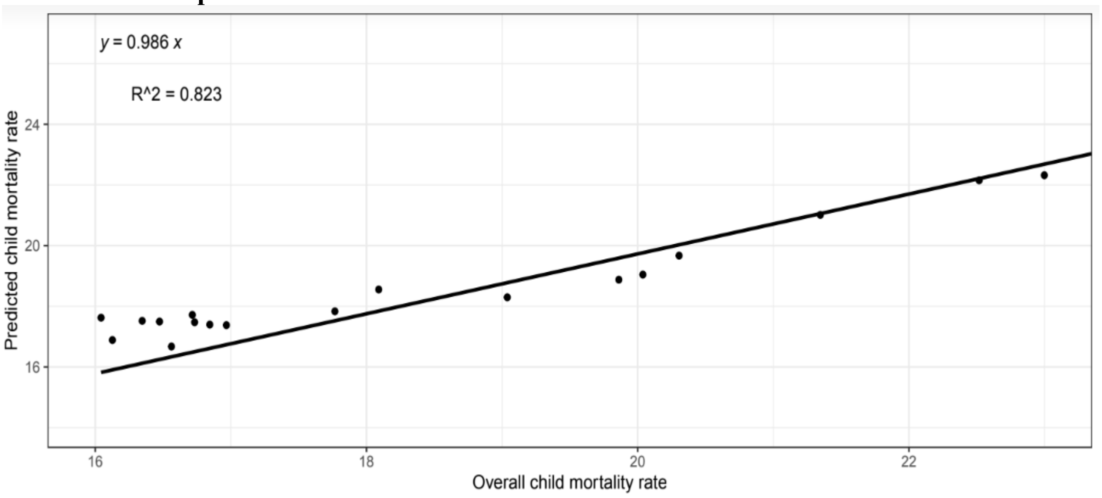

9. Sensitivity analysis

To evaluate how a lengthening of the economic crisis could affect mortality rates we additionally modelled the impact of austerity and social protection mitigation considering two additional poverty scenarios (see eFigures 19 and 20). The eTable 28 and 29 are consistent with the results found for the poverty scenario 2 (see the main manuscript) showing that the averted deaths decrease as the policies conditions improve in terms of increase the coverage of the different social programs

© 2023 Cavalcanti DM et al. *JAMA Network Open.*

eTable 28. Rate ratio (RR) and cumulative difference in under-five deaths over the period 2020-2030 between alternative policy scenarios, according to different the Economic Crisis Scenario.

	Mitigation/Baseline=5%		Mitigation/Austerity=9.8%	
**Year**	**Rate Ratio (RR)**	**Li -Ls**	**Rate Ratio (RR)**	**Li -Ls**
		**Under 5 Mortality Rate**		
		Poverty scenario 1		
2020	0.959	0.949 - 0.970	0.955	0.949 – 1.000
2025	0.932	0.914 - 0.949	0.896	0.875 - 0.910
2030	0.918	0.889 - 0.938	0.859	0.832 - 0.850
Avoidable deaths	83,793	69,376 – 98,610	117,776	97,965 – 137,953
		Poverty scenario 2		
2020	0.949	0.935 - 0.962	0.943	0.934 – 1.000
2025	0.890	0.864 - 0.917	0.860	0.831 - 0.855
2030	0.882	0.855 - 0.909	0.828	0.796 - 0.848
Avoidable deaths	114,513	93,846 – 135,896	153,601	127,441 - 180,600
		Poverty scenario 3		
2020	0.949	0.935 - 0.962	0.943	0.934 - 1.000
2025	0.881	0.854 - 0.910	0.850	0.820 - 0.854
2030	0.870	0.842 - 0.900	0.816	0.782 - 0.845
Avoidable deaths	124,308	101,821 – 147,540	161,572	134,380 - 189,549

Source: Author's data analysis for 8,103 municipalities in Brazil, Ecuador and Mexico (BEM countries).
Note: Data are in Rate Ratio (RR) coefficients (95% CI) unless otherwise specified.

eTable 29. Estimated Overall and averted deaths.

Economic Crisis Scenario	Poverty Scenario	Deaths (Overall)	Averted Deaths	% Averted Deaths
Baseline	SC1	801,847	83,793.03	10.45
	SC2	841,387	11,4512.8	13.61
	SC3	853,178	12,4308.1	14.57
Austerity	SC1	835,882	117,775.71	14.08
	SC2	880,236	153,601.1	17.44
	SC3	890,697	161,572.4	18.13

Source: Author's data analysis for 8,103 municipalities in Brazil, Ecuador and Mexico (BEM countries).

10. Main Limitations

The main limitation of the study is the uncertainty around the future macroeconomic scenarios in Brazil, Ecuador and Mexico due to the current extremely unstable political and economic situation, which creates uncertainty around the forecasting of poverty rates, income, and the other independent variables. For that reason, several scenarios have been simulated in sensitivity analyses which produced comparative findings. Another limitation is that the modelling of austerity measures is focused on CCT programs as there is strong evidence that these policies confer protective effects for overall and childhood morbidity and mortality from previous studies.[22]

Our estimates of the impact of austerity measures on under-5 age groups are probably conservative as they do not reflect constraints in other areas of public spending e.g., education, housing and other welfare programs which have known impacts on poverty and health. Moreover, austerity measures recently enshrined in the constitution of BEM countries means that public spending will only increase in line with inflation, which will not account for the demographic growth of the population, its ageing processes, and growing costs associated with new healthcare treatments and technologies.[23–25] Another limitation of the study is that we do not model the impact of the increased coverage of CCT on poverty rate dynamics, assuming that poverty rates influence CCT coverage and not the contrary.

© 2023 Cavalcanti DM et al. *JAMA Network Open.*

This is mainly due to the assumption that WB simulations of poverty increase during economic crisis already account for CCT effects, and because reliable parameters were not available at the moment of writing.

© 2023 Cavalcanti DM et al. *JAMA Network Open.*

REFERENCES

1 Rasella D, Aquino R, Santos CAT, Paes-Sousa R, Barreto ML. Effect of a conditional cash transfer programme on childhood mortality: A nationwide analysis of Brazilian municipalities. *The Lancet* 2013; **382**: 57–64.

2 Ramos D, da Silva NB, Ichihara MY, *et al.* Conditional cash transfer program and child mortality: A cross-sectional analysis nested within the 100 Million Brazilian Cohort. *PLoS Med* 2021; **18**: e1003509.

3 Rasella D, Basu S, Hone T, Paes-Sousa R, Ocké-Reis CO, Millett C. Child morbidity and mortality associated with alternative policy responses to the economic crisis in Brazil: A nationwide microsimulation study. *PLoS Med* 2018; **15**: e1002570.

4 Hone T, Mirelman AJ, Rasella D, *et al.* Effect of economic recession and impact of health and social protection expenditures on adult mortality: a longitudinal analysis of 5565 Brazilian municipalities. *Lancet Glob Health* 2019; **7**: e1575–83.

5 Moncayo AL, Granizo G, Grijalva MJ, Rasella D. Strong effect of Ecuador's conditional cash transfer program on childhood mortality from poverty-related diseases: A nationwide analysis. *BMC Public Health* 2019; **19**. DOI:10.1186/s12889-019-7457-y.

6 Fernald LC, Gertler PJ, Neufeld LM. Role of cash in conditional cash transfer programmes for child health, growth, and development: an analysis of Mexico's Oportunidades. *The Lancet* 2008; **371**: 828–37.

7 Levy S. Progress against poverty: sustaining Mexico's Progresa-Oportunidades program. Brookings Institution Press, 2007 https://books.google.com/books?hl=en&lr=&id=31zD_hBNs4YC&oi=fnd&pg=PP1&dq=Progress+Against+Poverty:+Sustaining+Mexico%E2%80%99s+Progresa-Oportunidades+Program&ots=FE7IvgRtov&sig=jX5abZqFabhHhwowta5yK9TcBbc (accessed Nov 14, 2022).

8 Dávila Lárraga LG. How Does Prospera Work? 2016 https://publications.iadb.org/publications/english/viewer/How-does-Prospera-Work-Best-Practices-in-the-Implementation-of-Conditional-Cash-Transfer-Programs-in-Latin-America-and-the-Caribbean.pdf (accessed Jan 12, 2023).

9 Rasella D, Alves FJO, Rebouças P, *et al.* Long-term impact of a conditional cash transfer programme on maternal mortality: a nationwide analysis of Brazilian longitudinal data. *BMC Med* 2021; **19**. DOI:10.1186/s12916-021-01994-7.

10 Riumallo-Herl C, Aguila E. The effect of old-age pensions on health care utilization patterns and insurance uptake in Mexico. *BMJ Glob Health* 2019; **4**. DOI:10.1136/bmjgh-2019-001771.

11 Hernández Licona G et al., de la Garza T, Zamudio J, Yaschine I. El Progresa-Oportunidades-Prospera, a veinte años de su creación, 1st edn. Consejo Nacional de Evaluación de la Política de Desarrollo Social, 2019 https://www.coneval.org.mx/Evaluacion/IEPSM/Documents/Libro_POP_20.pdf (accessed Nov 16, 2022).

© 2023 Cavalcanti DM et al. *JAMA Network Open.*

12 Martínez D, Borja T, Medellín N, Cueva P. ¿Cómo funciona el Bono de Desarrollo Humano? 2017 https://www.academia.edu/download/72227652/be4f0990662c39d0f1b9ef9988a9e7f224b5.pdf (accessed Nov 16, 2022).

13 de Souza PH, Osorio RG, Paiva LH, Soares S. The effects of the Bolsa Família Program on poverty and inequality: a review of the first fifteen years. 2019 https://www.econstor.eu/handle/10419/211450 (accessed Nov 16, 2022).

14 AbouZahr C, de Savigny D, Mikkelsen L, *et al.* Civil registration and vital statistics: progress in the data revolution for counting and accountability. *The Lancet* 2015; **386**: 1373–85.

15 Mikkelsen L, Phillips DE, AbouZahr C, *et al.* A global assessment of civil registration and vital statistics systems: monitoring data quality and progress. *The Lancet* 2015; **386**: 1395–406.

16 Lourenço Tavares de Andrade C, Landmann Szwarcwald C. Desigualdades sócio-espaciais da adequação das informações de nascimentos e óbitos do Ministério da Saúde, Brasil, 2000-2002 Socio-spatial inequalities in the adequacy of Ministry of Health data on births and deaths at the municipal level in Brazil. mai, 2007 http://www.datasus.

17 Rasella D, Aquino R, Barreto ML. Impact of the Family Health Program on the quality of vital information and reduction of child unattended deaths in Brazil: an ecological longitudinal study. 2010 http://www.biomedcentral.com/1471-2458/10/380.

18 Lawlor DA, Tilling K, Davey Smith G. Triangulation in aetiological epidemiology. *Int J Epidemiol* 2017; : dyw314.

19 Rasella D, Basu S, Hone T, Paes-Sousa R, Ocké-Reis CO, Millett C. Child morbidity and mortality associated with alternative policy responses to the economic crisis in Brazil: A nationwide microsimulation study. *PLoS Med* 2018; **15**. DOI:10.1371/journal.pmed.1002570.

20 Mariani, C. B. Gomes, E. C. Cenci, D. R. Queiroz RF de. Financiamento da Assistência Social no Brasil Nota Técnica de Monitoramento (2019). 2019.

21 Salata ARicardo, Ribeiro MGomes. Boletim Desigualdade nas Metrópoles (n. 09). Observatório das Metrópoles. Porto Alegre/RS, 2022.

22 Rasella D, Aquino R, Santos CAT, Paes-Sousa R, Barreto ML. Effect of a conditional cash transfer programme on childhood mortality: A nationwide analysis of Brazilian municipalities. *The Lancet* 2013; **382**. DOI:10.1016/S0140-6736(13)60715-1.

23 Paiva AB, Mesquita ACS, Jaccoud L, Passos L. [The new tax regime and its implications for social assistance policy in Brazil.] [Portuguese]. *Instituto de Pesquisa Econômica Aplicada* 2016; **27**.

24 Vieira, F. S. Benevides RPDS. [The new tax regime and its implications for social assistance policy in Brazil.] [Portuguese]. Technical Note No.27. 2016.

25 Rossi P, Dweck E. Impacts of the New Fiscal Regime on health and education Impactos. *Cad Saude Publica* 2016; **32**.

© 2023 Cavalcanti DM et al. *JAMA Network Open.*
