# Supplementary material for: Evaluation and Forecasting Analysis of the Association of Conditional Cash Transfer With Child Mortality in Latin America, 2000-2030
Source: JAMA Netw Open. 2023 Jul 14;6(7):e2323489. doi: 10.1001/jamanetworkopen.2023.23489 (PMC10349336; doi:10.1001/jamanetworkopen.2023.23489)
Supplement: Supplement 2. — Data Sharing Statement [file jamanetwopen-e2323489-s002.pdf]

Data Sharing Statement

Cavalcanti. Evaluation and Forecasting Analysis of the Association of Conditional Cash Transfer With Child Mortality in Latin America, 2000-2030. *JAMA Netw Open*. Published July 14, 2023. doi:10.1001/jamanetworkopen.2023.23489

Data
Data available: No
